# Muscle‐Derived Small Extracellular Vesicles Mediate Exercise‐Induced Cognitive Protection in Chronic Cerebral Hypoperfusion

**DOI:** 10.1002/advs.202410209

**Published:** 2025-04-24

**Authors:** Huawei Lin, Lianhua Yin, Weilin Liu, Rui Li, Tao Jiang, Minguang Yang, Yajun Cao, Sinuo Wang, Yan Yu, Cong Chen, Xiaoqin Guo, Wenju Wang, Huanhuan Liu, Yaling Dai, Jiamin Yan, Yanting Lin, Yanyi Ding, Chendong Ruan, Lei Yang, Tiecheng Wu, Jing Tao, Lidian Chen

**Affiliations:** ^1^ College of Rehabilitation Medicine Fujian University of Traditional Chinese Medicine Fuzhou Fujian 350122 China; ^2^ The Institution of Rehabilitation Industry Fujian University of Traditional Chinese Medicine Fuzhou Fujian 350122 China; ^3^ The Second Affiliated Hospital of Fujian University of Traditional Chinese Medicine Fuzhou Fujian 350003 China; ^4^ National‐Local Joint Engineering Research Center of Rehabilitation Medicine Technology Fujian University of Traditional Chinese Medicine Fuzhou Fujian 350122 China; ^5^ Provincial and Ministerial Co‐founded Collaborative Innovation Center of Rehabilitation Technology Fujian University of Traditional Chinese Medicine Fuzhou Fujian 350122 China; ^6^ Key Laboratory of Cognitive Rehabilitation of Fujian Province Affiliated Rehabilitation Hospital of Fujian University of Traditional Chinese Medicine Fuzhou Fujian 350001 China; ^7^ Traditional Chinese Medicine Rehabilitation Research Center of State Administration of Traditional Chinese Medicine Fujian University of Traditional Chinese Medicine Fuzhou Fujian 350122 China

**Keywords:** exercise, extracellular vesicles, miRNAs, synaptic plasticity, vascular cognitive impairment

## Abstract

Physical exercise protects against cognitive impairment caused by chronic cerebral hypoperfusion (CCH). However, the mechanisms through which exercise sends signals from the periphery to the central nervous system remain incompletely understood. This study demonstrated that exercise promotes the secretion of muscle‐derived small extracellular vesicles (sEVs), which facilitate interorgan communication between the muscle and the brain. Systematic delivery of muscle‐derived sEVs enhances synaptic plasticity and alleviated cognitive impairment in CCH. Notably, miRNA sequencing reveal miR‐17/20a‐5p as key cargos in sEVs involved in the exercise‐induced muscle‐brain crosstalk. Muscle‐derived sEVs are also identified as the primary source of swimming‐induced miR‐17/20a‐5p in circulating sEVs. Mechanistically, miR‐17/20a‐5p binds to the DEP‐domain containing mTOR‐interacting protein (DEPTOR) and activates the mammalian target of rapamycin (mTOR) pathway in the hippocampus. Depletion of miR‐17/20a‐5p from muscle‐derived sEVs impairs the exercise‐induced enhancement of synaptic plasticity and cognitive function. Moreover, overexpression of DEPTOR in the hippocampus attenuates the cognitive benefits of exercise. Conversely, hippocampus‐specific activation of mTOR reverses these effects, highlighting the crucial role of mTOR in mediating the positive effects of exercise. Collectively, these findings identify miR‐17/20a‐5p in muscle‐derived sEVs as the exercise‐induced myokine with potent effects on the brain, emphasizing the therapeutic potential of exercise in managing cognitive impairment.

## Graphical abstract

sEVs have a critical role in orchestrating interorgan crosstalk and mediating exercise‐induced therapeutic effects. Lin et al. demonstrates that sEVs miR‐17/20a‐5p mediates the muscle‐brain crosstalk and emphasizes the central role of mTOR signaling in executing molecular programs that can protect brain health in response to exercise.

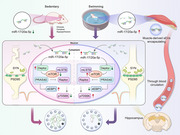

## 1 Introduction

Chronic cerebral hypoperfusion (CCH) is characterized by a prolonged insufficiency of cerebral blood flow, affecting either the entire brain or specific regions. CCH leads to an imbalance in cerebral metabolism and structural damage, resulting in vascular cognitive impairment.^[^ ^1^ ^]^ Currently, no satisfactory treatments are available for CCH. Exercise induces profound physiological responses across multiple tissues^[^ ^2^, ^3^ ^]^ and is widely regarded as a promising non‐pharmacological strategy for maintaining and improving brain function.^[^ ^4^, ^5^ ^]^ Meta‐analyses have demonstrated that exercise has several beneficial effects on cognitive function in stroke survivors.^[^ ^6^ ^]^ Specifically, exercise improves memory and executive function in patients with vascular cognitive impairment (VCI).^[^ ^7^ ^]^ The hippocampus, a brain region essential for learning and memory, is particularly responsive to exercise.^[^ ^8^, ^9^ ^]^ Research shows that exercise significantly increases hippocampal volume and enhances memory.^[^ ^10^ ^]^ It also promotes hippocampal‐related cognition by stimulating neurogenesis^[^ ^11^ ^]^ and improving synaptic plasticity.^[^ ^12^, ^13^ ^]^ In a previous study, we showed that long‐term aerobic exercise alleviated spatial memory impairment in mice with chronic cerebral hypoperfusion by increasing dendritic spine density and enhancing long‐term potentiation (LTP).^[^ ^14^ ^]^ Despite these findings, the mechanisms through which exercise exerts its beneficial effects on neural and synaptic function remain poorly understood.

Exerkines are signaling molecules released from various organs and tissues in response to exercise. They play a critical role in facilitating cell‐to‐cell communication and mediating the therapeutic effects of exercise.^[^ ^15^ ^]^ Cells can package exerkines into small extracellular vesicles (sEVs), which enter the circulation and travel from the periphery to the central nervous system.^[^ ^16^, ^17^ ^]^ Exercise has been shown to promote the rapid release of sEVs into circulation.^[^ ^18^, ^19^ ^]^ These sEVs, measuring 50–150 nm in diameter, possess the ability to cross the blood–brain barrier.^[^ ^20^ ^]^ Studies consistently support the therapeutic potential of sEVs secreted by specific cell types or carrying specific cargos in enhancing functional recovery after cerebral ischemic diseases.^[^ ^21^, ^22^, ^23^, ^24^ ^]^ Furthermore, sEVs are increasingly recognized as promising drug delivery vehicles in various disease contexts.^[^ ^25^, ^26^, ^27^, ^28^ ^]^

Myokines, such as interleukin‐6 (IL‐6), irisin, and apelin, are exerkines secreted by skeletal muscles and have been extensively studied.^[^ ^19^ ^]^ Additionally, growing evidence indicates that microRNAs (miRNAs), a class of small noncoding RNA molecules, regulate gene expression in response to physiological and pathological stress^[^ ^29^, ^30^ ^]^ and serve as critical mediators of exercise‐induced signaling.^[^ ^31^ ^]^ Notably, exercise alters the miRNA composition in sEVs released by organs including skeletal muscle, heart, liver, and brain. However, the underlying mechanisms of their exercise‐driven secretion remain unclear. These miRNA‐enriched sEVs can modulate downstream signaling pathways in distant tissues and organs.^[^ ^32^, ^33^, ^34^, ^35^ ^]^ Identifying novel exerkines, therefore, offers a valuable opportunity to deepen our understanding of the benefits of exercise and develop innovative strategies to treat human diseases.

In this study, we demonstrated that muscle‐derived sEVs play a pivotal role in exercise‐induced synaptic remodeling and cognitive protection in a rat model of CCH. Systemic administration of muscle‐derived sEVs from exercised CCH rats to unexercised CCH rats partially transferred the physiological benefits of exercise to the brain. We further identified miR‐17/20a‐5p, enriched in muscle‐derived sEVs from exercised CCH rats, as a key mediator of these benefits. miR‐17/20a‐5p promoted activation of the mechanistic target of rapamycin (mTOR) pathway, enhanced synaptic plasticity, and mitigated CCH‐induced cognitive impairment. Our findings also highlight the mTOR pathway as a common target of exerkines in the brain and demonstrate that mTOR activation can alleviate the cognitive and neurological deficits associated with CCH. Collectively, this study identifies miR‐17/20a‐5p as a novel myokine and reveals a mechanism through which muscle‐derived sEVs mediate exercise‐induced cognitive protection. By establishing muscle‐brain crosstalk, we provide a mechanistic explanation for the systemic and endogenous benefits of exercise. These findings offer valuable insights for developing clinical treatments and rehabilitation strategies based on non‐pharmacological approaches.

## 2 Results

### 2.1 sEVs Contribute to Swimming‐Induced Improvements in Synaptic Plasticity and Cognition in CCH Rats

**Figure 1:**
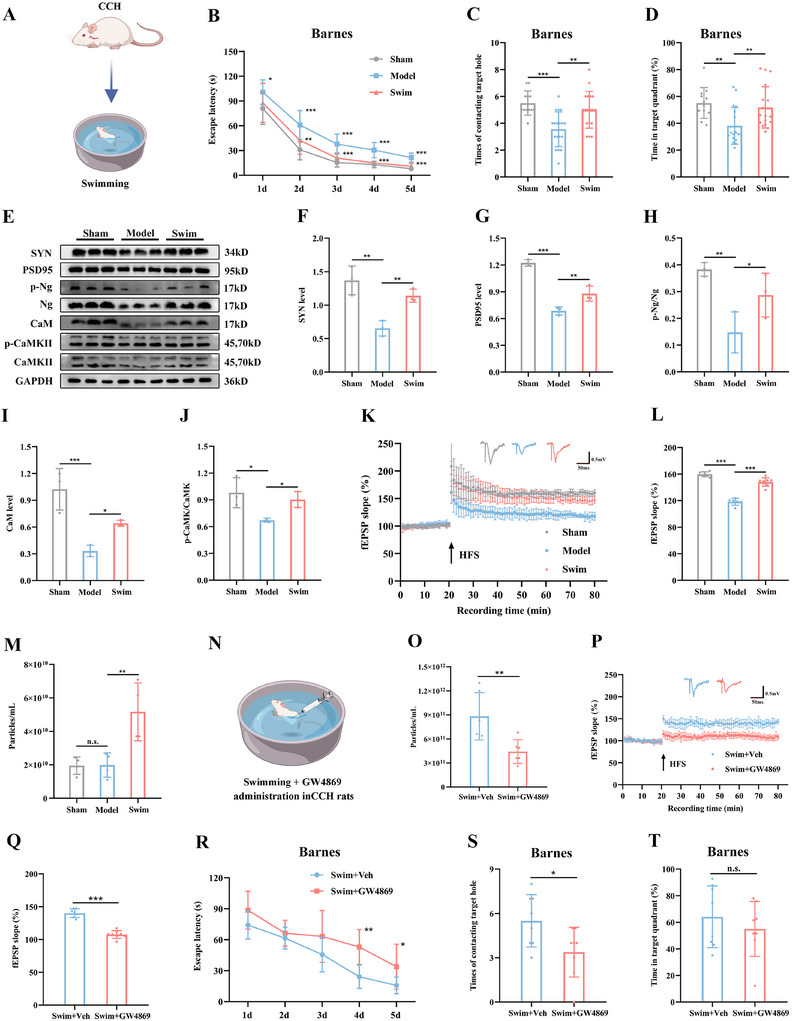
Swimming training enhances synaptic plasticity and alleviates cognitive deficits via sEVs in CCH rats. A) Schematic diagram illustrating the swimming training protocol for rats subjected to bilateral common carotid artery operation. Rats underwent swimming training for 5 d every week over a period of 4 weeks. B) Latency to reach the target hole during the learning stage of the Barnes maze after swimming training. C,D) Quantification of the number of contacts with the target hole (C) and the duration percentage in the target quadrant (D) during the probe trial following swimming training. E) Representative western blots depicting synapse‐associated proteins in rat hippocampi after swimming training. F–J) Quantitative analysis of SYN (F), PSD95 (G), p‐Ng/Ng (H), CaM (I), and p‐CaMKII/CaMKII (J) in the hippocampus. (K) Time‐plot of normalized fEPSP slopes recorded from hippocampal slices after swimming training. Representative traces of fEPSP from the Sham, Model, and Swim groups are shown. L) Histogram illustrating the average normalized fEPSP slope during the last 10 min of recording following HFS, represented as a percentage of the baselines for each group. M) Average concentration of serum sEVs in rats after swimming training. N) Schematic diagram depicting CCH rats receiving swimming training and GW4869 administration (1.25 mg k^−1^g, 3 times per week for 4 weeks). O) Average concentration of serum sEVs in rats after GW4869 treatment. P) Time‐plot of normalized fEPSP slopes recorded from hippocampal slices after GW4869 treatment. Representative traces of fEPSP from the Swim+Veh and Swim+GW4869 groups. Q) Histogram presenting the average normalized fEPSPs slope during the last 10 min of recording following HFS, represented as a percentage of the baselines for each group. R) Latency to reach the target hole during the learning stage of the Barnes maze after GW4869 treatment. S,T) Quantification of the number of contacts with the target hole (S) and the duration percentage in the target quadrant (T) during the probe trial after vehicle or GW4869 treatment. Data are presented as the mean ± SD. Sham group *n* = 12, Model and Swim groups *n* = 18 (B‐D). *n* = 3 per group (E–J). Sham and Model group *n* = 6, Swim groups *n* = 8 (K and L). *n* = 4 per group (M). *n* = 6 per group (O). Swim+NC group *n* = 7, Swim+GW4869 group *n* = 8 (P and Q). *n* = 8 per group (R–T). ^*^ *p* < 0.05, ^**^ *p* < 0.01, ^***^ *p* < 0.001. n.s., not significant. Statistical analysis was performed using two‐way ANOVA for repeated measures (B and R), one‐way ANOVA with Bonferroni post‐hoc comparisons (C, D, F–J, L, and M), and unpaired two‐tailed Student's *t*‐test (O, Q, S, and T). ANOVA, Analysis of variance; CCH, Chronic cerebral ischemia; fEPSP, Field excitatory post‐synaptic potential; HFS, High frequency stimulation; sEVs, Small extracellular vesicles; Swim, CCH rats undergoing swimming training.

In this study, we developed a preclinical model of CCH by performing bilateral common carotid artery ligation in SD rats (referred to as CCH rats) and subjected them to a 4‐week swimming training regimen (**Figure** **1**A). To assess blood flow and cognitive function in CCH rats, we conducted 3D‐TOF imaging and the Morris water maze (MWM) test following surgery. These assessments confirmed significant occlusion of the bilateral common carotid arteries (Figure S1A, Supporting Information) and marked cognitive impairments in the rats postsurgery (Figure S1B–D, Supporting Information), indicating successful model establishment. We also verified that swimming training did not induce anxiety‐like behaviors in CCH rats through the open field test (OFT) (Figure S1E–G, Supporting Information).

We next investigated the impact of exercise on cognitive function in CCH rats. In the Barnes maze, swimming training significantly reduced the latency to find the escape box, increased the number of contacts with the target hole, and elevated the proportion of time spent in the target quadrant (Figure 1B–D). Furthermore, there was no significant difference in movement speed between the three groups during the test (Figure S1H, Supporting Information). Behavioral phenotyping revealed that swimming‐trained CCH rats exhibited a substantial increase in the autonomous alternation rate and novel object preference compared to their untrained counterparts (Figure S1I,J, Supporting Information). Synaptic plasticity, which underlies learning and memory processes, was also evaluated in this context.^[^ ^47^ ^]^ Normal behavior relies on the proper functioning of synapses, and disruptions in synaptic activity have been implicated in various diseases.^[^ ^48^ ^]^ We observed that swimming training upregulated the presynaptic marker synapsin (SYN) and the postsynaptic marker postsynaptic density protein 95 (PSD95), activated the Neurogranin (Ng)‐calcium (CaM)‐CaM‐dependent protein kinase II (CaMKII) pathway (Figure 1E–J), and enhanced hippocampal LTP (Figure 1K,L) in CCH rats. These findings demonstrate that swimming training provides significant protection against memory impairments induced by CCH.

Emerging evidence suggests that sEVs play a critical role in mediating the beneficial effects of exercise.^[^ ^49^ ^]^ We isolated sEVs from the serum of rats at the end of the 4‐week training period and characterized them using transmission electron microscopy (TEM), nanoparticle tracking analysis (NTA), and western blotting (WB) (Figure S1K, Supporting Information). NTA results revealed that swimming training increased the number of sEV particles (Figure 1M). To determine whether sEVs contributed to the cognitive improvements observed, we intraperitoneally administered GW4869, a well‐established inhibitor of sEVs biogenesis, or vehicle control to CCH rats 1 h before each swimming session, three times a week for 4 weeks (Figure 1N). GW4869 significantly reduced sEV levels after swimming (Figure 1O) and attenuated the positive effects of swimming on hippocampal LTP (Figure 1P,Q). Consistent with these findings, GW4869 treatment also impaired performance in the maze task (Figure 1R–T; Figure S1L,M, Supporting Information), supporting the notion that sEVs mediate the exercise‐induced cognitive improvements in CCH rats.

### 2.2 Delivery of Circulating sEVs from Exercised CCH Rats Improves Synaptic Plasticity and Cognition in CCH Rats

**Figure 2:**
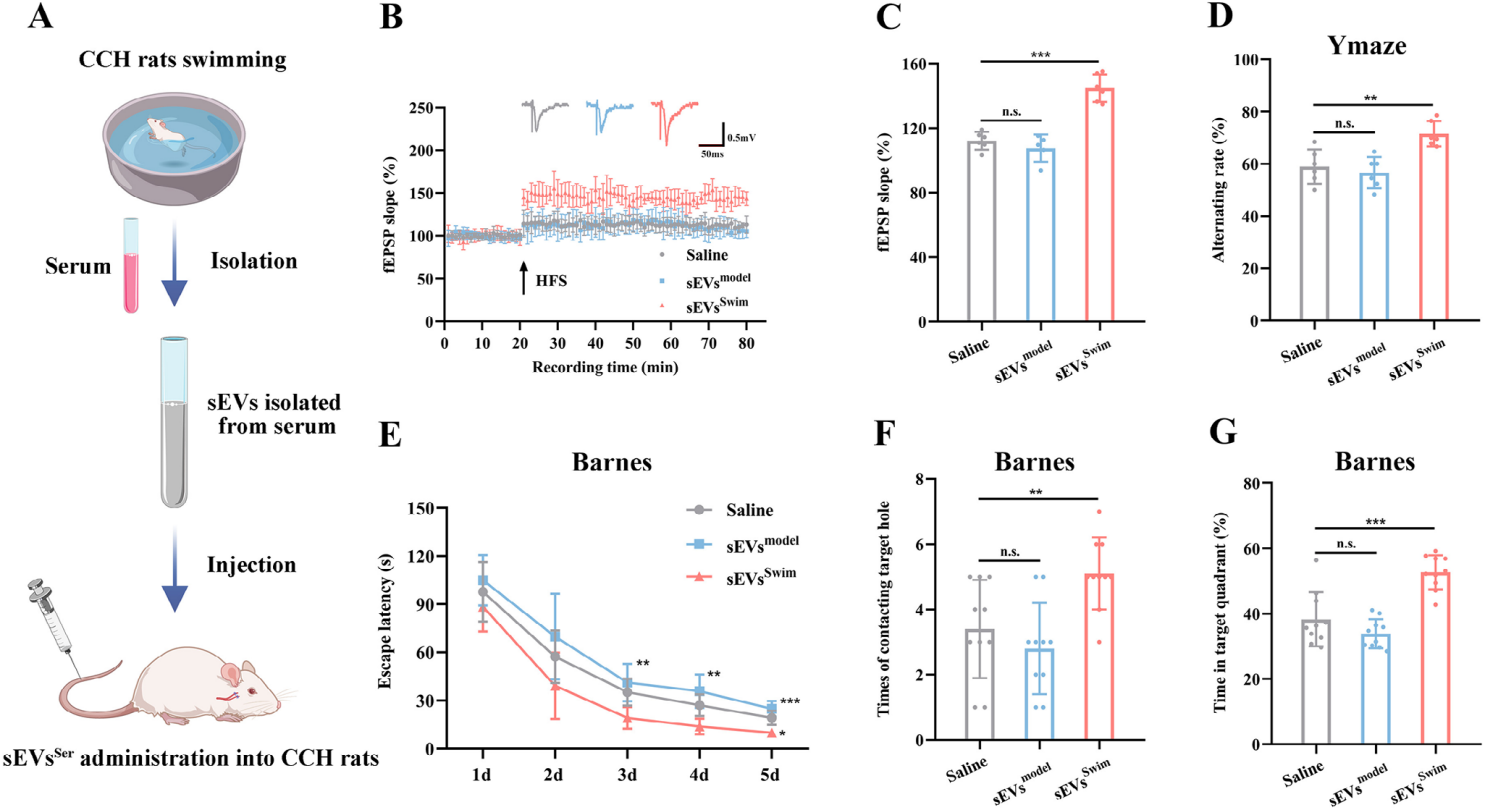
Treatment of swimming‐derived serum sEVs mimics the exercise‐induced positive effect for synaptic plasticity and cognitive impairment in CCH rats. A) Schematic diagram illustrating CCH rats receiving administration of swimming‐derived serum sEVs (100 µg sEVs per administration). B) Time‐plot of normalized fEPSP slopes recorded from hippocampal slices after delivery of swimming‐derived serum sEVs. Representative traces of fEPSP from the Saline, sEVs^Model^, and sEVs^Swim^ groups. C) Histogram representing the average normalized fEPSP slopes during the last 10 min of recording following HFS, represented as a percentage of the baselines for each group. D) Spontaneous alternation rate in the Y maze after delivery of swimming‐derived serum sEVs. E) Latency to reach the target hole during the learning stage of the Barnes maze after delivery of swimming‐derived serum sEVs. F,G) Quantification of the number of contacts with the target hole (F) and the duration percentage in the target quadrant (G) during the probe trial after delivery of swimming‐derived serum sEVs. Data are presented as the mean ± SD. Saline and sEVs^Swim^ groups *n* = 6, sEVs^Model^ group *n* = 5 (B and C). *n* = 6 per group (D). *n* = 10 per group (E–G). ^*^ *p* < 0.05, ^**^ *p* < 0.01, ^***^ *p* < 0.001. Statistical analysis was performed using one‐way ANOVA with Bonferroni post‐hoc comparisons (C, D, F, and G) and two‐way ANOVA for repeated measures (E). ANOVA, Analysis of variance; CCH, Chronic cerebral hypoperfusion; fEPSP, Field excitatory post‐synaptic potential; HFS, High frequency stimulation; sEVs, Small extracellular vesicles. sEVs^Ser^, sEVs isolated from serum; sEVs^Model^, serum sEVs from sedentary CCH rats; sEVs^Swim^, serum sEVs from swimming CCH rats.

Building on the observed cognitive benefits of exercise and the critical role of sEVs in mediating these effects, we next investigated whether the advantages of swimming could be transferred to unexercised animals through the administration of circulating sEVs derived from exercised animals. We collected blood samples and isolated serum sEVs from both unexercised CCH rats and those that had undergone swimming training. An independent cohort of CCH rats then received weekly intravenous injections of either saline or PKH26‐labeled sEVs for 4 weeks (**Figure** **2**A). PKH26 was detected in the hippocampus following sEVs administration and the PKH26 was not detected with injecting the mixture of PBS and PKH26, confirming that the exogenously injected sEVs crossed the blood‐brain barrier (Figure S2A,B Supporting Information). In addition, we used an experimental protocol based on orthogonal biological reactions to achieve surface modification of sEVs to further confirm their blood‐brain barrier permeability, which is different from labeling sEVs with lipophilic dyes. ‌Fluorescein Isothiocyanate (FITC)^+^ sEVs can also be detected in the hippocampus (Figure S2C, Supporting Information). Therefore, these results demonstrated that sEVs derived from rat serum can effectively cross the blood‐brain barrier. To determine the cell types that took up the sEVs in the hippocampus, the brain sections were processed for immunofluorescence using markers for neurons (NeuN^+^), astrocytes (GFAP^+^), and microglia (Iba‐1^+^). In the brain, red‐colored PKH26^+^ particles were mostly localized in the neurons. We also detected labeled sEVs within Iba‐1^+^ microglial cells, but there were little sEVs took up in GFAP^+^ astrocytes (Figure S2D–F, Supporting Information). To further assess whether the delivery of exercise‐derived circulating sEVs could replicate the cognitive protective effects of swimming, we recorded field potentials and evaluated performance in the Y maze and Barnes maze. Compared to rats treated with saline or sEVs from unexercised CCH rats, the slope of the excitatory postsynaptic potential (fEPSP) in the hippocampus was significantly increased in rats treated with sEVs from exercised CCH rats (Figure 2B,C). In the Y maze, rats treated with sEVs from exercised animals exhibited higher autonomous alternation rate (Figure 2D). In the Barnes maze, rats receiving sEVs from swimming‐trained CCH rats exhibited a significantly shorter escape latency, contacted the target hole more frequently and spent more time in the target quadrant compared to the other groups compared to those receiving sEVs from unexercised rats or saline (Figure 2E–G). Together, these findings suggest that circulating sEVs from exercised CCH rats can transfer the benefits of exercise, enhancing hippocampal synaptic plasticity and alleviating memory impairments in unexercised CCH rats.

### 2.3 miR‐17/20a‐5p in Circulating sEVs Mediates Swimming‐Induced Cognitive Protection by Targeting DEPTOR

**Figure 3:**
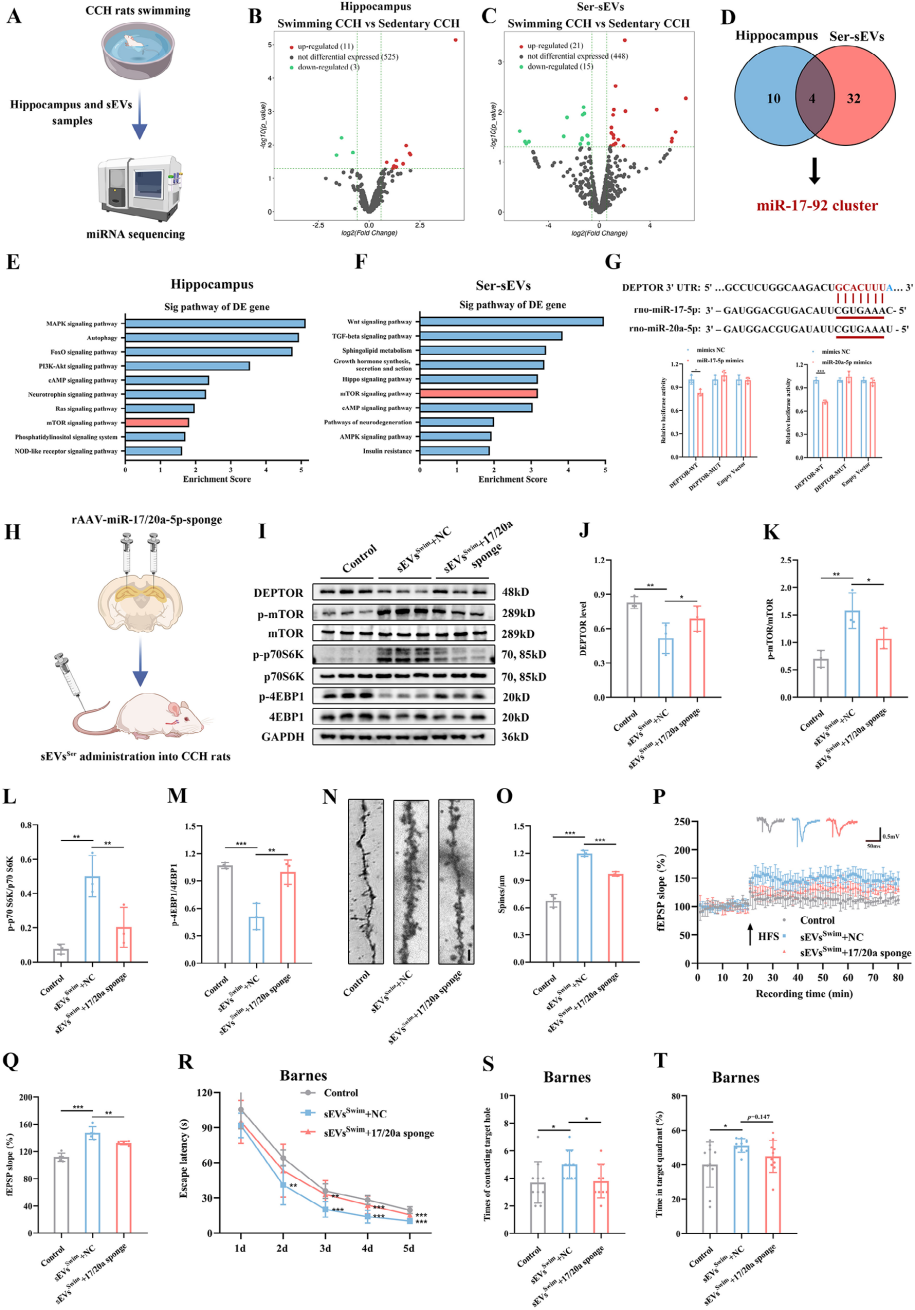
miR‐17/20a‐5p facilitate cognitive protection provided by swimming‐derived serum sEVs in CCH rats. A) Schematic diagram illustrating miRNA sequencing in the hippocampus and serum sEVs after swimming training. B,C) Volcano map presenting the relative differences in miRNA expression between sedentary CCH rats and exercised CCH rats in the hippocampus (B) and serum sEVs (C). D) Venn diagram illustrating unique and overlapping differential miRNAs in the hippocampus and serum sEVs after swimming training. E,F) Signaling pathway enrichment analysis of differential miRNAs in the hippocampus (E) and serum sEVs (F). G) Predicted binding sequences of miR‐17/20a‐5p in the 3′UTR of DEPTOR. Luciferase reporter assays for miR‐17/20a‐5p and the DEPTOR 3′UTR with native or mutant binding sites are shown. H) Schematic diagram depicting rats receiving rAAV injection in the hippocampus followed by serum sEVs treatment (100 µg sEVs per administration). I) Representative western blots showing putative target genes and downstream mTOR pathway in the hippocampus treated with rAAV‐miR‐17/20a‐5p‐sponge. J–M) Quantitative analysis of DEPTOR (J), p‐mTOR/mTOR (K), p‐p70S6K/p70S6K (L), and p‐4EBP1/4EBP1 (M) in the hippocampus. N) Representative images of Golgi staining in the hippocampus after rAAV injection in the hippocampus. Scale bar: 2 µm. O) Quantitative analysis of dendritic spine density determined by Golgi staining. P) Time‐plot of normalized fEPSP slopes recorded from hippocampal slices after rAAV injection in the hippocampus. Representative traces of fEPSP from theControl, sEVs^Swim^+NC, and sEVs^Swim^+17/20a sponge groups. Q) Histogram representing the average normalized fEPSP slopes during the last 10 min of recording following HFS, represented as a percentage of the baselines for each group. R) Latency to reach the target hole during the learning stage of the Barnes maze after rAAV injection in the hippocampus. S,T) Quantification of the number of contacts with the target hole (S) and the duration percentage in the target quadrant (T) during the probe trial after rAAV injection in the hippocampus. Data are presented as the mean ± SD. *n* = 3 per group (B, C, G, I–M, and O). Control group *n* = 5, sEVs^Swim^+NC group *n* = 5, sEVs^Swim^+17/20a sponge group *n* = 6 (P and Q). *n* = 10 per group (R–T). ^*^ *p* < 0.05, ^**^ *p* < 0.01, ^***^ *p* < 0.001. Statistical analysis was performed using one‐way ANOVA with Bonferroni post‐hoc comparisons (J–M, Q, S, and T), two‐way ANOVA (G) and two‐way ANOVA for repeated measures (R). 3′UTR, 3′Untranslated region; ANOVA, Analysis of variance; DEPTOR, DEP‐domain containing mTOR‐interacting protein; fEPSP, Field excitatory postsynaptic potential; HFS, High frequency stimulation; mTOR, Mechanistic target of rapamycin; rAAV, Recombination adeno‐associated virus; SD, Standard deviation; sEVs, Small extracellular vesicles; sEVs^Swim^, serum sEVs from swimming CCH rats.

miRNA cargos within sEVs play a pivotal role in regulating exercise‐induced synaptic remodeling and cognitive protection.^[^ ^50^, ^51^, ^52^ ^]^ To investigate whether miRNAs in sEVs from exercised rats contribute to the observed protection against CCH‐induced cognitive deficits, we conducted miRNA sequencing (miRNA‐seq) on hippocampal tissue and peripheral sEVs, comparing miRNA expression profiles between exercised and unexercised CCH rats (**Figure** **3**A). In the hippocampus, we identified 14 differentially expressed miRNAs (fold change >1.5; *P*<0.05; Figure 3B), with 11 miRNAs upregulated and 3 downregulated in exercised compared to unexercised CCH rats. In the sEVs, 36 miRNAs exhibited differential expression (fold change >1.5; *P*<0.05; Figure 3C), including 21 miRNAs that were upregulated and 15 that were downregulated in exercised versus unexercised CCH rats. The miRNAs showing the most substantial differential expression were miR‐17‐5p and miR‐20a‐5p in the hippocampus, and miR‐17‐1‐3p and miR‐20b‐3p in circulating sEVs, suggesting that the miR‐17‐92 cluster may play a central role in mediating the cognitive benefits of swimming training (Figure 3D).

To further explore this, we measured the expression levels of the miR‐17‐92 cluster in hippocampal tissue and serum sEVs via qPCR. This analysis confirmed a significant increase in miR‐17‐5p and miR‐20a‐5p expression in CCH rats following swimming training (Figure S3A,B, Supporting Information), reflecting consistent trends across both the hippocampus and sEVs. To determine whether the exercise‐induced upregulation of miR‐17/20a‐5p affects multiple brain regions or is localized to specific sites, we examined miR‐17/20a‐5p levels in the prefrontal cortex (PFC), a key region involved in cognitive function. We found no differences between the sham and CCH groups, but swimming training significantly elevated miR‐17/20a‐5p levels in the PFC, indicating that the exercise‐induced increase in miR‐17/20a‐5p specifically enhances cognition in a brain region‐ and CCH‐dependent manner (Figure S3C, Supporting Information).

Further Kyoto Encyclopedia of Genes and Genomes (KEGG) enrichment analysis revealed that mTOR signaling was significantly enriched in both the hippocampus and sEVs of swimming‐trained CCH rats (Figure 3E,F). miRNAs exert their biological effects by binding to specific seed sequences within the 3′ untranslated regions (UTRs) of target mRNAs, thereby inhibiting translation or promoting mRNA degradation. To identify potential target genes for miR‐17‐5p and miR‐20a‐5p, we conducted a bioinformatics analysis using miRDB and TargetScan. Among the predicted target genes, three were involved in the pathways identified in the KEGG enrichment analysis: phosphatase and tensin homolog deleted on chromosome ten (PTEN), Tre2‐Bub2‐Cdc16‐1 domain family member 7 (TBC1D7), and DEP‐domain containing mTOR‐interacting protein (DEPTOR). To validate whether miR‐17‐5p and miR‐20a‐5p directly target these genes, we constructed luciferase reporter plasmids for each of the three genes, fused to either their native 3′ UTR or a mutated 3′ UTR. Co‐transfection of miR‐17/20a‐5p mimics with these plasmids resulted in significantly reduced luciferase activity only when DEPTOR's wild‐type 3′ UTR was present. No significant change in luciferase activity was observed with any of the other wild‐type or mutated constructs. These results suggest that miR‐17‐5p and miR‐20a‐5p specifically bind to the 3′ UTR of DEPTOR, but not to those of PTEN or TBC1D7 (Figure 3G; Figure S3D–G, Supporting Information).

To assess whether inhibiting miR‐17/20a‐5p could negate the protective effects of sEVs derived from trained rats on CCH‐induced cognitive impairment, we injected rAAV‐miR‐17/20a‐5p‐sponge or a vector control into the hippocampus of CCH rats. The viral delivery and expression were confirmed through fluorescence imaging and qPCR analysis of hippocampal tissue (Figure 3H; Figure S3H,I, Supporting Information). Inhibition of miR‐17/20a‐5p led to increased DEPTOR expression, which in turn inactivated the mTOR pathway by preventing 70 kDa ribosomal protein S6 kinase (p70S6K) phosphorylation and enhancing eIF4E‐binding protein 1 (4EBP1) phosphorylation in the hippocampus of CCH rats treated with sEVs (Figure 3I–M). Immunohistochemistry, Golgi staining, and electrophysiological recordings demonstrated that miR‐17/20a‐5p inhibition downregulated synaptic markers SYN and PSD95 (Figure S3J,K, Supporting Information), reduced dendritic spine density (Figure 3N,O), and diminished the slope of fEPSP in the hippocampus of CCH rats administered with sEVs (Figure 3P,Q). These changes reversed the positive effects of sEVs being isolated from exercised rats. Behavioral tests also reflected this reversal. Rats treated with AAV‐miR‐17/20a‐5p sponge exhibited significantly longer escape latencies in the Barnes maze compared to those injected with the control vector (Figure 3R). Furthermore, in the probe test, inhibition of miR‐17/20a‐5p resulted in fewer contacts with the target holes and reduced time spent in the target quadrant by rats treated with exercise‐derived sEVs (Figure 3S,T). Collectively, these results underscore the crucial role of miR‐17/20a‐5p in mediating the cognitive protective effects of exercise in CCH rats.

**Figure 4:**
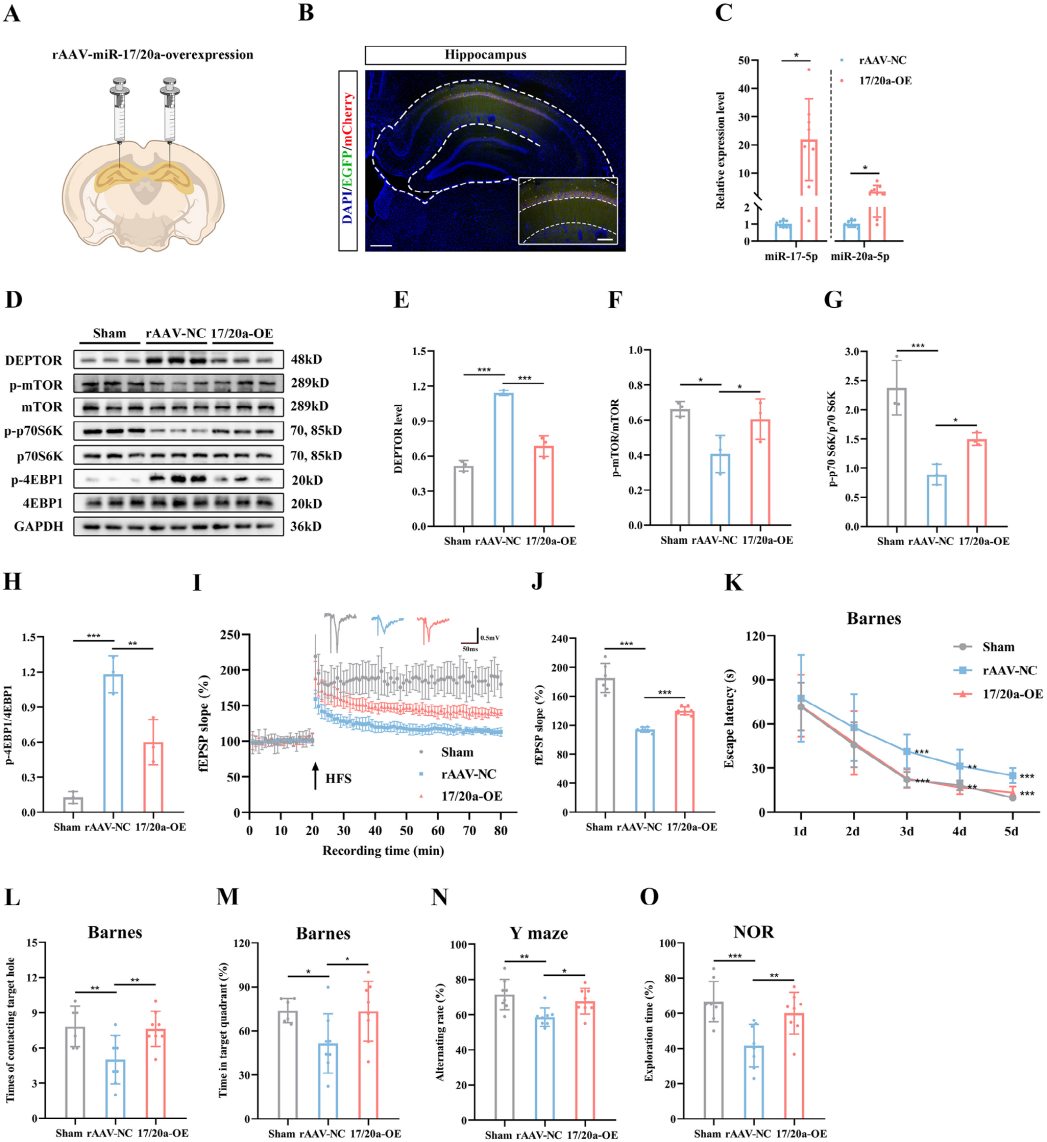
Hippocampal miR‐17/20a‐5p overexpression rescues cognitive deficits in CCH rats. A) Schematic diagram of rats receiving rAAV injection in the hippocampus. B) Representative immunofluorescent images showing the site of viral transfection. Scale bar, 500 µm in panoramic views and 200 µm in enlarged images. C) Levels of miR‐17/20a‐5p in the hippocampus after AAV injection. D) Representative western blots of DEPTOR and of the downstream mTOR pathway in hippocampi treated with rAAV‐miR‐17/20a‐overexpression. E–H) Quantitative analysis of DEPTOR (E), p‐mTOR/mTOR (F), p‐p70S6K/p70S6K (G), and p‐4EBP1/4EBP1 (H) in the hippocampus. I) Time‐plot of normalized fEPSP slopes recorded from hippocampal slices after rAAV injection in the hippocampus. Representative traces of fEPSP in the Sham, rAAV‐NC, and 17/20a‐OE groups. J) Histogram representing the average normalized fEPSP slopes during the last 10 min of recording following HFS, represented as a percentage of the baselines for each group. K,H) Latency to reach the target hole in the learning stage of the Barnes maze after rAAV injection in the hippocampus. L,M) Number of contacts with the target hole (L) and duration percentage in the target quadrant (M) during the probe trial after rAAV injection in the hippocampus. N) Spontaneous alternation rate in the Y maze test after rAAV injection in the hippocampus. O) Discrimination index detected 1 h after the learning stage in the NOR test after rAAV injection in the hippocampus. Data are presented as the mean ± SD. *n* = 8 per group (C, N, and O). *n* = 3 per group (D–H). Sham group *n* = 6, rAAV‐NC group *n* = 6, 17/20a‐OE group *n* = 8 (I and J). Sham group *n* = 6, rAAV‐NC group *n* = 8, 17/20a‐OE group *n* = 8 (K–M). ^*^ *p* < 0.05, ^**^ *p* < 0.01, ^***^ *p* < 0.001. Statistical analysis was performed using unpaired two‐tailed Student's *t*‐test (C), one‐way ANOVA with Bonferroni post‐hoc comparisons (E–H, J, and L–O) and two‐way ANOVA for repeated measures (K). ANOVA, Analysis of variance; fEPSP, Field excitatory postsynaptic potential; HFS, High frequency stimulation; mTOR, Mechanistic target of rapamycin; NOR, Novel object recognition; rAAV, Recombination adeno‐associated virus; SD, Standard deviation.

To explore whether targeting miR‐17/20a‐5p could provide therapeutic benefits for CCH‐induced cognitive impairment, we injected rAAV vectors expressing miR‐17/20a‐5p or a control rAAV‐NC construct into the hippocampus of CCH rats (**Figure** **4**A–C). Overexpression of miR‐17/20a‐5p, compared to the control group, reduced DEPTOR levels and activated the mTOR pathway (Figure 4D–H). Moreover, rats with miR‐17/20a‐5p overexpression showed enhanced hippocampal synaptic plasticity (Figure 4I,J) and exhibited improved performance in behavioral tests (Figure 4K–O). These results indicate that circulating sEVs from swimming‐trained rats deliver miR‐17/20a‐5p to target tissues, activating the mTOR pathway and offering protection against CCH‐induced cognitive decline.

### 2.4 Engineered RVG‐sEVs Enriched in miR‐17/20a‐5p Improve Synaptic Plasticity and Cognition in CCH Rats

**Figure 5:**
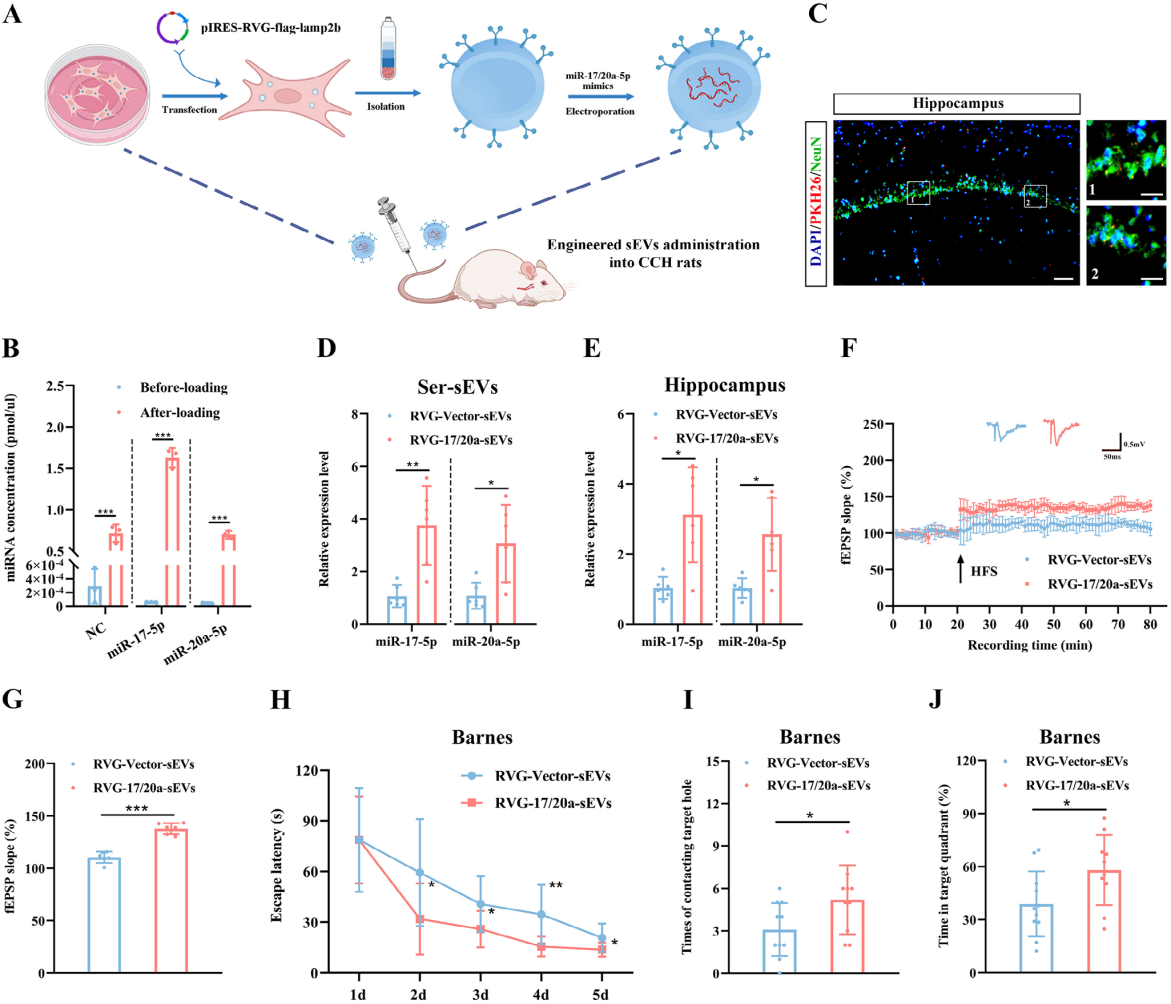
Administration of miR‐17/20a‐5p‐enriched RVG‐sEVs improves synaptic plasticity and cognitive function in CCH rats. A) Schematic diagram illustrating the production and harvest of engineered RVG‐sEVs for targeted miR‐17/20a‐5p delivery. Rats were injected via the tail vein once a week for 2 weeks, with each administration containing 100 µg of sEVs. B) Absolute qPCR analysis of negative control sequence levels in the RVG‐Vector‐sEVs and miR‐17/20a‐5p levels in RVG‐17/20a‐sEVs. C) Representative fluorescent image of the distribution of PKH26‐labeled sEVs in neurons of hippocampus after intravenous injection. Scale bar: 50 µm in left views and 10 µm in right enlarged images. D,E) Quantification of miR‐17/20a‐5p levels in serum sEVs (D) and hippocampi (E) after administration of engineered RVG‐sEVs. F) Time‐plot of normalized fEPSP slopes recorded from hippocampal slices after administration of engineered RVG‐sEVs. Representative traces of fEPSPs from the RVG‐Vector‐sEVs and RVG‐17/20a‐sEVs groups. G) Histogram representing the average normalized fEPSP slopes during the last 10 min of recording following HFS, represented as a percentage of the baselines for each group. H) Latency to reach the target hole during the learning stage of the Barnes maze after administration of engineered RVG‐sEVs. I,J) Quantification of the number of contacts with the target hole (I) and the duration percentage in the target quadrant (J) during the probe trial after administration of engineered RVG‐sEVs. Data are presented as the mean ± SD. *n* = 3 per group (B). *n* = 6 per group (D–G). RVG‐Vector‐sEVs group *n* = 11, RVG‐17/20a‐sEVs group *n* = 10 (H–J). ^*^ *p* < 0.05, ^**^ *p* < 0.01, ^***^ *p* < 0.001. Statistical analysis was performed using unpaired two‐tailed Student's *t*‐test (B, D, E G, I, and J), and two‐way ANOVA for repeated measures (H). ANOVA, Analysis of variance; fEPSP, Field excitatory post‐synaptic potential; HFS, High frequency stimulation; qPCR, Quantitative real‐time polymerase chain reaction; RVG‐sEVs, Rabies virus glycoprotein‐ Small extracellular vesicles; SD, Standard deviation; sEVs, Small extracellular vesicles.

From a translational standpoint, sEVs offer significant advantages for therapeutic applications in neurological diseases due to their low immunogenicity, biodegradability, and ability to encapsulate endogenous bioactive molecules while crossing the blood‐brain barrier.^[^ ^16^, ^53^ ^]^ To investigate whether sEVs enriched with miR‐17/20a‐5p can replicate the cognitive benefits of exercise, we transfected miRNA mimics into sEVs via electroporation to overexpress miR‐17/20a, leading to the secretion of additional miRNAs. To improve the targeting efficiency of this delivery system to the brain, we fused the lysosome‐associated membrane glycoprotein 2 (LAMP2), a membrane protein of sEVs, with the neuron‐specific RVG peptide, creating RVG‐sEVs^[^ ^54^ ^]^ (**Figure** **5**A). We used TEM, NTA, and WB to characterize RVG‐sEVs before and after loading the therapeutic cargo. These analyses confirmed that the cargo loading process did not alter the size, morphology, or membrane composition of the RVG‐sEVs (Figure S4A–E, Supporting Information). Additionally, we validated the successful loading of miR‐17/20a‐5p into RVG‐sEVs through quantitative PCR (Figure 5B).

We intravenously administered RVG‐sEVs containing miR‐17/20a‐5p mimics or a negative control (NC) to CCH rats once a week for two weeks. The expression of RVG‐sEVs in the hippocampus was confirmed via immunofluorescence and labeled sEVs were mostly localized in the neurons (Figure 5C). As anticipated, the engineered sEVs secreted additional miRNAs and successfully increased the levels of miR‐17/20a‐5p in the hippocampus (Figure 5D,E). Furthermore, treatment with RVG‐sEVs loaded with miR‐17/20a‐5p enhanced the slope of the fEPSP in the hippocampus of CCH rats, compared to those treated with NC‐loaded RVG‐sEVs (Figure 5F,G). In behavioral tests, CCH rats treated with miR‐17/20a‐5p‐enriched RVG‐sEVs exhibited a shorter escape latency during the learning phase (Figure 5H), increased contact with target holes, and spent more time in the target quadrant in the probe test, compared to the control group (Figure 5I,J). Additionally, these rats demonstrated a significantly higher autonomous alternation rate and greater preference for novel objects compared to the controls (Figure S4F,G, Supporting Information). These results suggest that targeted delivery of RVG‐sEVs enriched with miR‐17/20a‐5p to the brain can effectively improve synaptic plasticity and cognitive function in CCH rats, mimicking the effects of exercise.

### 2.5 Muscle‐Derived sEVs are Main Contributors to Swimming‐Induced miR‐17/20a‐5p Upregulation in Circulating sEVs of CCH Rats

To investigate how swimming—an exercise that primarily engages peripheral muscles—affects the central nervous system, we examined the cellular origin of miR‐17/20a‐5p in circulating sEVs. We quantified miR‐17/20a‐5p levels in various tissues, including the heart, liver, spleen, lung, kidney, and skeletal muscle, of CCH rats after swimming training and in unexercised rats, using qPCR. Swimming training significantly elevated miR‐17/20a‐5p levels in the liver and skeletal muscle compared to unexercised rats (Figure S5A,B, Supporting Information). Interestingly, while CCH rats showed reduced miR‐17/20a‐5p levels in the liver compared to sham controls, CCH induction did not notably alter miR‐17/20a‐5p levels in skeletal muscle (Figure S5C,D, Supporting Information). Additionally, we assessed the levels of miR‐17/20a‐5p precursors and found that swimming training increased pre‐miR‐17/20a‐5p levels in skeletal muscle but not in the liver or hippocampus (Figure S5E–G, Supporting Information).

In skeletal muscle, the expression levels of miR‐17/20a‐5p were higher in exercise‐trained rats compared to sham and CCH rats. This unexpected variation in miR‐17/20a‐5p expression across the three groups suggests that CCH may not induce significant muscle dysfunction. Rather, the observed changes in miR‐17/20a‐5p likely represent adaptive responses to exercise training. To further investigate this, we included a cohort of healthy SD rats and subjected them to 4 weeks of swimming training. Following the training, we measured miR‐17/20a‐5p expression in both skeletal muscle and hippocampus. Our results revealed that swimming training significantly increased the expression of miR‐17/20a‐5p in both tissues (Figure S5H,I, Supporting Information). Additionally, previous studies have demonstrated that exercise can enhance memory and executive function in healthy individuals.^[^ ^55^ ^]^ Therefore, we assessed the cognitive performance of these healthy, trained rats using behavioral tests and analyzed the correlation with miR‐17/20a‐5p expression levels. The findings showed that swimming training improved cognitive performance, as indicated by increased alternation rates in the Y‐maze and higher recognition indices in the novel object recognition (NOR) test (Figure S5J,K, Supporting Information). Moreover, we observed a significant correlation between elevated miR‐17‐5p levels in both muscle and hippocampus and improved cognitive performance in the Y‐maze and NOR tasks (Figure S5L–O, Supporting Information). These results identify miR‐17/20a‐5p as an exercise‐induced factor with potential cognitive benefits in rats.

**Figure 6:**
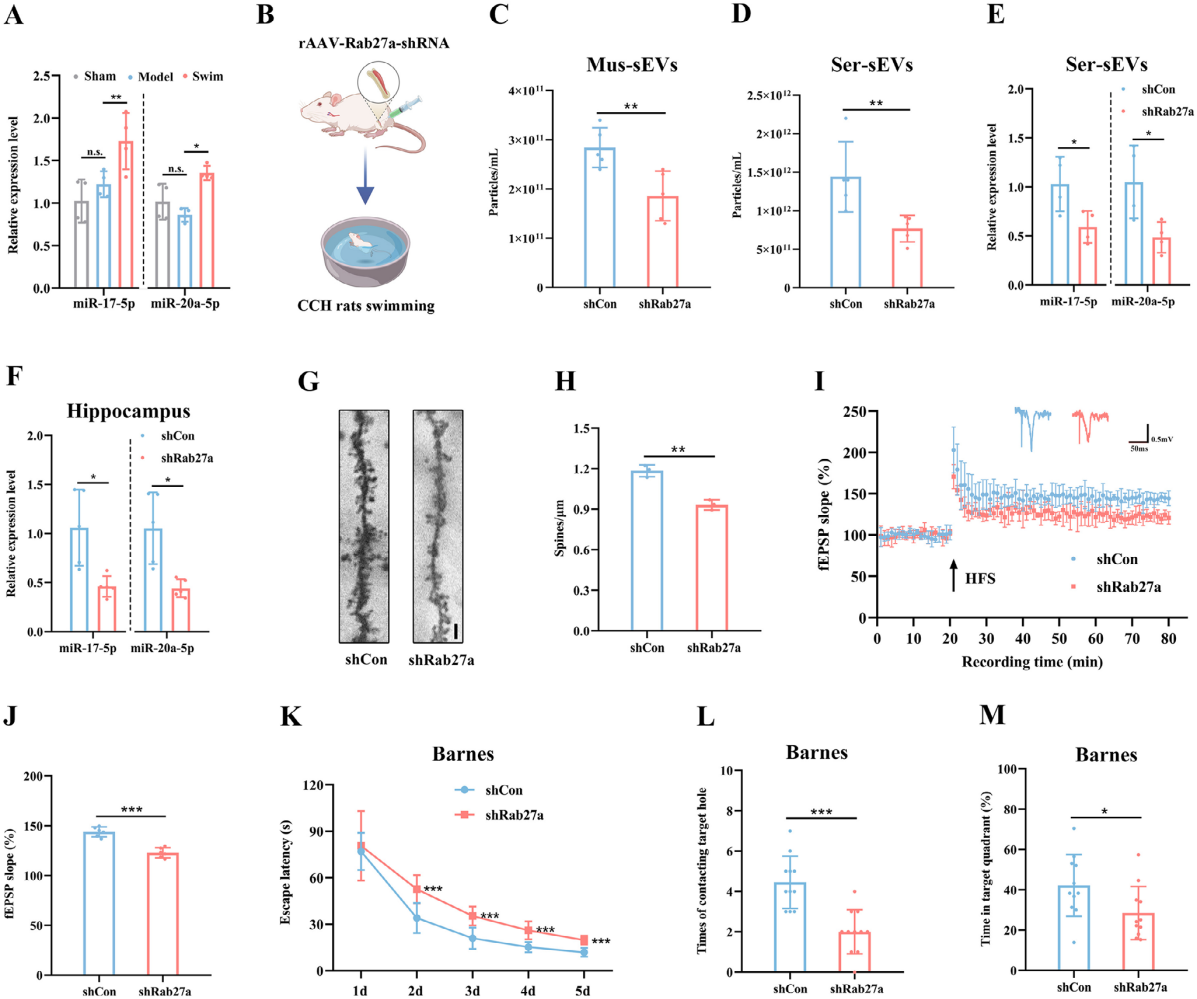
Muscle‐derived sEVs are the main source of the swimming‐induced miR‐17/20a‐5p increase in circulating sEVs of CCH rats. A) Levels of miR‐17/20a‐5p in the muscle‐derived sEVs of CCH rats after swimming training. B) Schematic diagram illustrating rats receiving in situ intramuscular injection of rAAV‐Rab27a‐shRNA followed by swimming training. C,D) Average concentration of muscle sEVs (C) and serum sEVs (D) in CCH rats after rAAV injection in the muscle. E,F) Levels of miR‐17/20a‐5p in the serum sEVs (E) and hippocampus (F) after rAAV injection in the muscle. G) Representative images of Golgi staining in the hippocampus after rAAV injection in the muscle. Scale bar: 2 µm. H) Quantitative analysis of dendritic spine density determined by Golgi staining. I) Time‐plot of normalized fEPSP slopes recorded from hippocampal slices after rAAV injection in the muscle. Representative traces of fEPSP from the shCon and shRab27a groups. J) Histogram presenting the average normalized fEPSP slopes during the last 10 min of recording following HFS, represented as a percentage of the baselines for each group. K) Latency to reach the target hole during the learning stage of the Barnes maze after rAAV injection in the muscle. L,M) Quantification of the number of contacts with the target hole (S) and the duration percentage in the target quadrant (T) during the probe trial after rAAV injection in the muscle. Data are presented as the mean ± SD. *n* = 4 per group (A and E). *n* = 5 per group (C, D, and F). *n* = 3 per group (H). *n* = 6 per group (I and J). *n* = 11 per group (K–M). ^*^ *p* < 0.05, ^**^ *p* < 0.01, ^***^ *p* < 0.001. n.s., not significant. Statistical analysis was performed using one‐way ANOVA with Bonferroni post‐hoc comparisons (A), unpaired two‐tailed Student's *t*‐test (C–F, H, J, L, and M) and two‐way ANOVA for repeated measures (K). ANOVA, Analysis of variance; CCH, Chronic cerebral ischemia; fEPSP, Field excitatory post‐synaptic potential; HFS, High frequency stimulation; sEVs, Small extracellular vesicles; Mus‐sEVs, sEVs isolated from skeletal muscle; Ser‐sEVs, sEVs isolated from serum.

Our results suggest that swimming training induces adaptive changes in skeletal muscle, which may help alleviate CCH‐induced cognitive dysfunction by promoting the release of additional miR‐17/20a‐5p into circulating sEVs. To investigate the role of muscle‐derived sEVs in this process, we isolated sEVs from the skeletal muscle of CCH rats following swimming training, adapting a recently described isolation strategy.^[^ ^40^, ^41^ ^]^ TEM, NTA, and WB analyses confirmed the successful isolation of muscle‐derived sEVs (Figure S6A–C, Supporting Information). Next, we performed miRNA sequencing on the sEVs isolated from skeletal muscle and compared their profiles across different experimental groups. The miRNA profiles of the sham and model groups were similar (Figure S6D, Supporting Information). However, swimming training significantly altered the miRNA expression profile, with miR‐17‐5p being a common gene upregulated in both comparisons (Figure S6E–G, Supporting Information). To quantify these changes, we measured miR‐17/20a‐5p expression in the isolated muscle sEVs using qPCR. Our results revealed a significant increase in miR‐17/20a‐5p levels in the sEVs from the swimming group, compared to both the model and sham groups, reflecting similar changes observed in the skeletal muscle (**Figure** **6**A).

To determine whether skeletal muscle secretes sEVs and if these vesicles can enter the brain in vivo, we developed rAAV vectors that encode the sEV marker CD63 fused to the enhanced green fluorescent protein (EGFP) reporter. This construct was driven by the muscle‐specific MHCK7 promoter to ensure expression solely in muscle cells.^[^ ^41^ ^]^ We locally injected the virus into the bilateral skeletal muscle (Figure S7A, Supporting Information). After 4 weeks, we observed high EGFP expression in the muscle fibers at the injection site (Figure S7B, Supporting Information). Furthermore, we detected fluorescence signals in the hippocampus, as well as EGFP^+^ sEVs in the serum (Figure S7C–F, Supporting Information). Quantification revealed a significant increase in EGFP^+^ EVs in the serum of rats receiving MHCK7‐CD63‐EGFP‐AAV injections following swimming training, compared to unexercised sham and model rats (Figure S7G, Supporting Information). These findings suggest that exercise enhances the secretion of muscle‐derived sEVs, which are subsequently transferred to the brain via the bloodstream.

To assess the role of muscle‐derived sEVs in cognitive protection in CCH rats, we developed an adenoviral vector system encoding Rab27a shRNA to silence Rab27a expression, a known regulator of EV biogenesis and secretion.^[^ ^56^, ^57^ ^]^ CCH rats received bilateral intramuscular injections of AAV‐Rab27a‐shRNA to inhibit muscle‐derived sEV secretion (Figure 6B), and successful infection of muscle cells was confirmed (Figure S7H, Supporting Information). Inhibition of Rab27a expression significantly reduced the number of sEVs in both skeletal muscle and serum (Figure 6C,D), accompanied by decreased levels of miR‐17/20a‐5p in sEVs from serum and hippocampus (Figure 6E,F). Interestingly, Rab27a knockdown led to increased DEPTOR expression, inactivation of the mTOR pathway (Figure S7I–M, Supporting Information), and enhanced dendritic spine density (Figure 6G,H), as well as an increased slope of fEPSP (Figure 6I,J). Behavioral tests revealed that compared to control rats receiving regular swimming training, rats with Rab27a knockdown exhibited impaired cognitive performance in the MWM, Y maze, and NOR tests (Figure 6K–M; Figure S7N,O, Supporting Information). These findings confirm that muscle‐derived sEVs are critical for exercise‐induced cognitive protection and that the increase in miR‐17/20a‐5p in circulating sEVs is primarily attributed to muscle‐derived sEVs secretion.

### 2.6 miR‐17/20a‐5p in the Muscle‐Derived sEVs Mediate Exercise‐Induced Synaptic Enhancement and Cognitive Protection in CCH Rats

**Figure 7:**
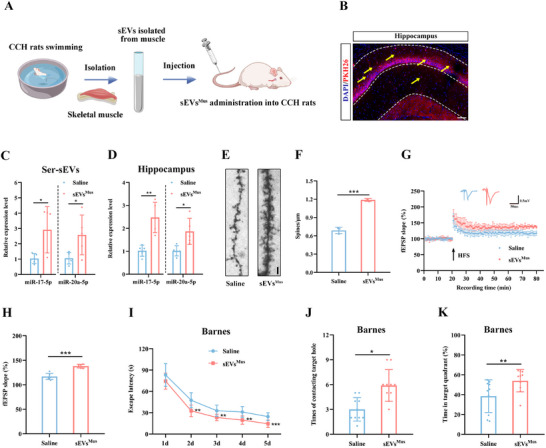
Treatment of swimming‐derived muscle sEVs mimics the exercise‐induced positive effect for synaptic plasticity and cognitive impairment in CCH rats. A) Schematic diagram illustrating CCH rats receiving administration of muscle‐derived sEVs from swimming CCH rats (100 µg sEVs per administration). B) Representative fluorescent image of sEVs in the hippocampus of rats injected with PKH26‐labeled sEVs. Scale bar: 100 µm. C,D) Levels of miR‐17/20a‐5p in the serum sEVs (C) and hippocampus (D) after treatment with muscle‐derived sEVs from swimming CCH rats. E) Representative images of Golgi staining in the hippocampus after delivery of swimming‐derived muscle sEVs. Scale bar: 2 µm. F) Quantitative analysis of dendritic spine density determined by Golgi staining. G) Time‐plot of normalized fEPSP slopes recorded from hippocampal slices after delivery of swimming‐derived muscle sEVs. Representative traces of fEPSP from the Saline and sEVs^Mus^ groups. (Hpostsynaptic density protein 95) Histogram representing the average normalized fEPSP slopes during the last 10 min of recording following HFS, represented as a percentage of the baselines for each group. I) Latency to reach the target hole during the learning stage of the Barnes maze after delivery of swimming‐derived muscle sEVs. J,K) Quantification of the number of contacts with the target hole (J) and the duration percentage in the target quadrant (K) during the probe trial after delivery of swimming‐derived muscle sEVs. Data are presented as the mean ± SD. *n* = 5 per group (C and D). *n* = 3 per group (F). *n* = 6 per group (G and H). *n* = 10 per group (I–K). ^*^ *p* < 0.05, ^**^ *p* < 0.01, ^***^ *p* < 0.001. Statistical analysis was performed using unpaired two‐tailed Student's *t*‐test (C, D, F, H, J, and K), and two‐way ANOVA for repeated measures (I). ANOVA, Analysis of variance; CCH, Chronic cerebral hypoperfusion; fEPSP, Field excitatory post‐synaptic potential; HFS, High frequency stimulation; SD, Standard deviation; sEVs, Small extracellular vesicles. sEVs^Mus^, muscle sEVs from swimming CCH rats.

To explore whether muscle‐derived sEVs can mimic the cognitive benefits of exercise, we isolated sEVs from the skeletal muscle of exercised rats. CCH rats were then intravenously injected once a week for four weeks with either saline or PKH26‐labeled sEVs derived from trained muscle (**Figure** **7**A). Fluorescence imaging revealed PKH26 signals in the hippocampus, indicating that the exogenously injected sEVs successfully crossed the blood–brain barrier (Figure 7B). Compared to the saline‐injected group, rats treated with muscle‐derived sEVs showed a significant increase in miR‐17/20a‐5p levels in both serum sEVs and the hippocampus (Figure 7C,D). Additionally, administration of muscle‐derived sEVs from exercised rats resulted in a reduction in DEPTOR levels and activation of the mTOR pathway (Figure S8A–E, Supporting Information). These molecular changes were accompanied by enhanced synaptic plasticity, as evidenced by an increase in dendritic spine density and the slope of fEPSP (Figure 7E–H). Behavioral assessments also revealed cognitive improvements, including a reduced escape latency, more frequent contact with the target hole, and greater time spent in the target quadrant in the Barnes maze (Figure 7I–K). Moreover, rats treated with muscle‐derived sEVs exhibited significantly improved performance in the autonomous alternation rate and novel object preference tests compared to controls (Figure S8F,G, Supporting Information). Together, these findings suggest that sEVs released from trained skeletal muscle play a key role in enhancing synaptic plasticity and protecting cognitive function in CCH rats.

The results of the previous experiments suggest that miR‐17/20a‐5p in skeletal muscle‐derived sEVs plays a key role in regulating synaptic plasticity. These sEVs serve as carriers that deliver bioactive components to the brain. Notably, the expression pattern of miR‐17/20a‐5p in muscle‐derived sEVs mirrors its expression in muscle tissue. Based on this observation, we hypothesized that targeting miR‐17/20a‐5p in muscle may be a critical mechanism for regulating exercise‐induced cognitive function in CCH rats.

**Figure 8:**
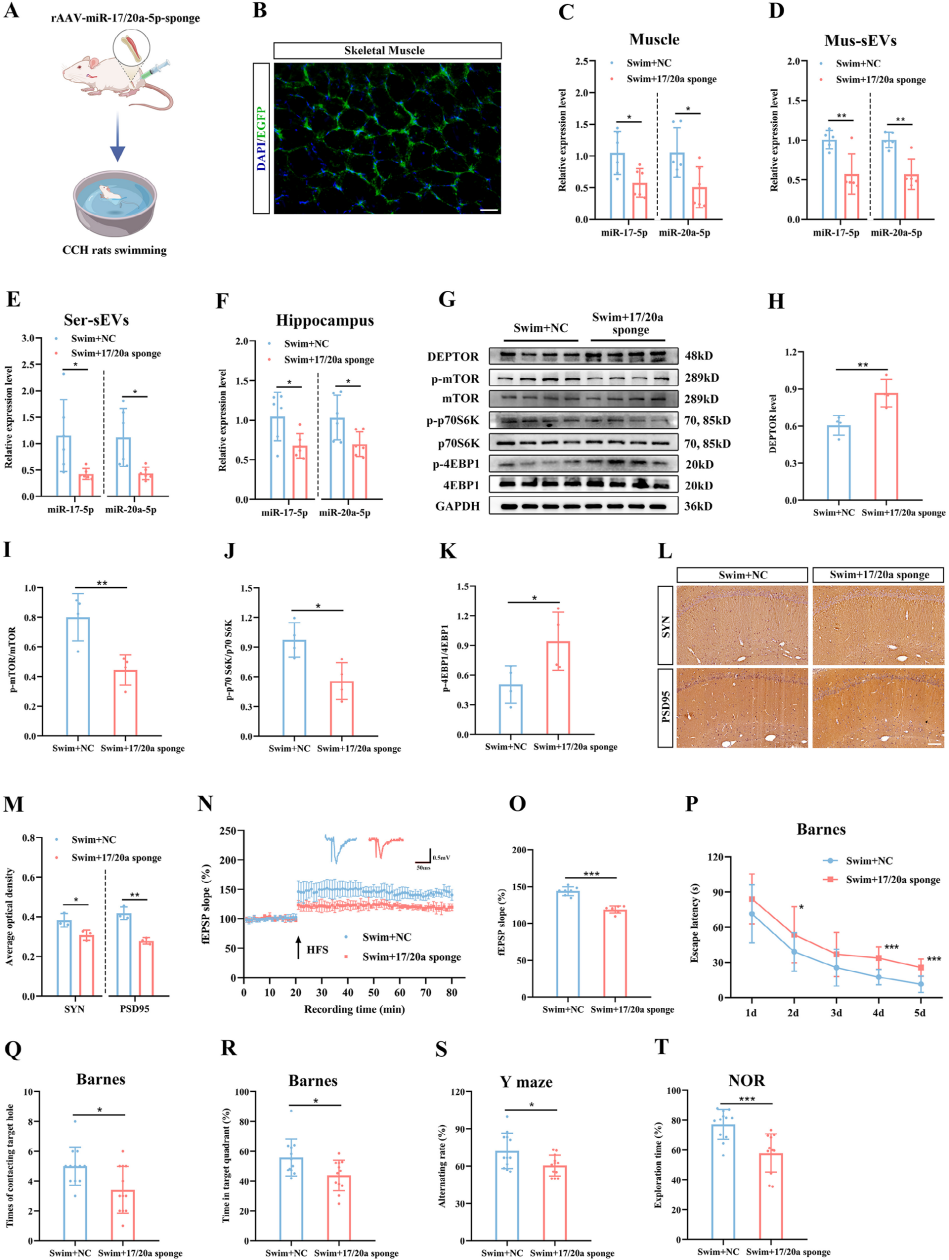
Inhibition of miR‐17/20a‐5p in exercised muscle blunts exercise‐induced cognitive protection in CCH rats. A) Schematic diagram illustrating rats receiving in situ intramuscular injection of rAAV‐miR‐17/20a‐5p‐sponge followed by swimming training. B) Representative immunofluorescent images showing the site of viral transfection. Scale bar: 50 µm. C–F) Levels of miR‐17/20a‐5p in the muscle (C), muscle sEVs (D), serum sEVs (E), and hippocampus (F) after rAAV injection in the muscle. G) Representative western blots of DEPTOR and of the downstream mTOR pathways in the hippocampus after rAAV injection in the muscle. H–K) Quantitative analysis of DEPTOR (H), p‐mTOR/mTOR (I), p‐p70S6K/p70S6K (J), and p‐4EBP1/4EBP1 (K) in the hippocampus. L) Representative immunohistochemical staining images showing the expression levels of SYN and PSD95 in the hippocampus after rAAV injection in the muscle. M) Quantitative analysis of SYN and PSD95 in the hippocampus. Scale bar, 100 µm. N) Time‐plot of normalized fEPSP slopes recorded from hippocampal slices after rAAV injection in the muscle. Representative traces of fEPSPs in the Swim+NC and Swim+17/20a sponge groups. O) Histogram representing the average normalized fEPSP slopes during the last 10 min of recording following HFS, represented as a percentage of the baselines for each group. P) Latency to reach the target hole during the learning stage of the Barnes maze after rAAV‐sponge infection in the muscle. Q,R) Number of contacts with the target hole (Q) and duration percentage in the target quadrant (R) during the probe trial after rAAV‐sponge infection in the muscle. S) Spontaneous alternation rate in the Y maze test after rAAV injection in the muscle. T) Discrimination index detected 1 hour after the learning stage in the NOR test after rAAV injection in the muscle. Data are presented as the mean ± SD. *n* = 6 per group (C, E, and F). *n* = 4 per group (G–K). *n* = 3 per group (M). *n* = 7 per group (N and O). *n* = 12 per group (P–T). ^*^ *p* < 0.05, ^**^ *p* < 0.01, ^***^ *p* < 0.001. Statistical analysis was performed using unpaired two‐tailed Student's *t*‐test (C–F, H–K, M, O, and Q–T) and two‐way ANOVA for repeated measures (N). ANOVA, Analysis of variance; fEPSP, Field excitatory postsynaptic potential; HFS, High frequency stimulation; mTOR, Mechanistic target of rapamycin; NOR, Novel object recognition; PSD95, Postsynaptic density protein 95; rAAV, Recombination adeno‐associated virus; sEVs, Small extracellular vesicles; SYN, Synapsin.

To test this hypothesis, we performed skeletal muscle‐specific knockdown of miR‐17/20a‐5p in CCH rats using intramuscular injection of rAAV‐miR‐17/20a‐5p‐sponge or a control vector (**Figure** **8**A,B). Treatment with rAAV‐miR‐17/20a‐5p‐sponge prevented the upregulation of miR‐17/20a‐5p in skeletal muscle, muscle‐derived sEVs, circulating sEVs, and the hippocampus of CCH rats following swimming training (Figure 8C–F). The knockdown of miR‐17/20a‐5p in the hippocampus resulted in increased DEPTOR levels and reduced mTOR activation (Figure 8G–K). Moreover, muscle‐specific knockdown of miR‐17/20a‐5p led to decreased expression of SYN and PSD95 (Figure 8L,M) and diminished the slope of fEPSP (Figure 8N,O) following swimming training, indicating that this treatment effectively blocked the beneficial effects of exercise on synaptic plasticity in CCH rats. Behavioral testing further confirmed this, as CCH rats treated with rAAV‐miR‐17/20a‐5p‐sponge displayed prolonged escape latencies in the Barnes maze (Figure 8P), fewer contacts with the target holes, and reduced time spent in the target quadrant (Figure 8Q,R) compared to control‐treated rats. Additionally, these rats showed a decreased autonomous alternation rate and reduced novel object preference following swimming training (Figure 8S,T). Together, these findings support the notion that miR‐17/20a‐5p is a pivotal bioactive cargo in muscle‐derived sEVs, mediating the muscle‐brain communication necessary for exercise‐induced cognitive protection.

To investigate whether various forms of aerobic exercise influence the central nervous system by regulating the same miRNAs identified in swimming‐trained rats, we examined the effects of treadmill exercise in CCH rats. Surprisingly, treadmill exercise did not increase the levels of miR‐17/20a‐5p (Figure S9A, Supporting Information). To explore this further, we performed high‐throughput sequencing to identify differentially expressed miRNAs in the hippocampus of CCH rats following treadmill exercise (Figure S9B, Supporting Information). Compared to sham controls, 11 miRNAs were upregulated and 4 were downregulated in the hippocampus of CCH rats (fold‐change ≥1.5, *P* ≤ 0.05, Figure S9C, Supporting Information). After treadmill exercise, 7 miRNAs were upregulated and 5 were downregulated in the hippocampus of CCH rats (fold‐change ≥1.5, *P* ≤ 0.05, Figure S9D, Supporting Information), with these changes validated by qPCR analysis (Figure S9E, Supporting Information). A comparison of the datasets revealed that miR‐144‐3p and miR‐130b‐3p were differentially regulated by both CCH induction and treadmill exercise (Figure S9F, Supporting Information). KEGG pathway enrichment analysis indicated that these differentially expressed miRNAs are involved in the mTOR signaling pathway (Figure S9G,H, Supporting Information). In conclusion, while treadmill and swimming training appear to regulate distinct sets of miRNAs in the hippocampus, the mTOR pathway emerges as a common target of both forms of aerobic exercise in CCH rats.

Brain structure and function analysis revealed that CCH induction reduced dendritic spine density and the slope of fEPSP in the hippocampus. In contrast, treadmill exercise increased dendritic spine density and enhanced the slope of fEPSP, suggesting that treadmill exercise, similar to swimming, improves synaptic plasticity in CCH rats (Figure S9I–L, Supporting Information). Additionally, treadmill exercise significantly improved cognitive performance in CCH rats, as evidenced by the MWM test (Figure S9M–O, Supporting Information). Importantly, treadmill training did not significantly affect swimming speed during the MWM test (Figure S9P, Supporting Information).

We focused on miR‐144‐3p as a potential exerkine and used online bioinformatics tools, including miRDB and TargetScan, to identify its putative target genes (Figure S10A, Supporting Information). Among the candidate targets, we selected PTEN, a gene involved in regulating the mTOR signaling pathway, for further investigation. Using a dual‐luciferase reporter assay, we identified the binding sites of miR‐144‐3p on the PTEN locus. Our results showed that miR‐144‐3p mimics significantly reduced the luciferase activity of vectors expressing PTEN fused with the wild‐type 3′ UTR, while fusion to a mutant 3′ UTR did not affect luciferase activity (Figure S10A, Supporting Information). These findings suggest that miR‐144‐3p can directly bind to PTEN. To assess whether miR‐144‐3p regulates the downstream effects of PTEN, CCH rats were injected with either rAAV‐miR‐144‐3p‐sponge or rAAV‐NC into the hippocampus (Figure S10B–D, Supporting Information).

Treadmill exercise in CCH rats reduced PTEN levels in the hippocampus and subsequently increased the activity of the PI3K‐AKT‐mTOR signaling pathway, compared to unexercised CCH rats. Treatment with rAAV‐miR‐144‐3p‐sponge, but not with rAAV‐NC, attenuated these effects (Figure S10E–I, Supporting Information), suggesting that exercise‐induced miR‐144‐3p plays a critical role in regulating the activation of the PI3K‐AKT‐mTOR pathway by modulating PTEN expression in the hippocampus of CCH rats. To further validate this, we repeated the synaptic anatomical, functional tests, and behavioral assessments. These results showed that treatment with rAAV‐miR‐144‐3p‐sponge in treadmill‐exercised CCH rats led to a decrease in dendritic spine density and a reduction in the slope of fEPSP in the hippocampus, compared to rAAV‐NC treatment (Figure S10J–M, Supporting Information). Correspondingly, behavioral tests revealed that rAAV‐miR‐144‐3p‐sponge treatment reduced the cognitive benefits of treadmill exercise, as evidenced by prolonged escape latency, increased crossing times on the escape platform, and reduced time spent in the target quadrant in the MWM (Figure S10N–P, Supporting Information). These findings suggest that although treadmill exercise regulates different miRNAs compared to swimming, both aerobic exercises effectively enhance synaptic plasticity and alleviate memory impairment in CCH rats by targeting key miRNAs in the hippocampus via the mTOR pathway. However, further investigation is required to clarify the specific mechanisms and transfer routes involved.

Given the critical role of the mTOR pathway in mediating both swimming‐ and treadmill‐induced cognitive improvements, we sought to determine whether activation of this pathway is necessary for exercise‐induced cognitive benefits. To assess this, we injected CCH rats with rapamycin or a vehicle control intraperitoneally before subjecting them to swimming training to inhibit mTOR activation (Figure S11A, Supporting Information). Western blot analysis showed reduced phosphorylation of key mTOR complex proteins, including mTOR and p70S6K, alongside increased phosphorylation of 4EBP1 in the hippocampus of rapamycin‐treated rats compared to controls (Figure S11B–E, Supporting Information). In addition, measurements of hippocampal field potential revealed a reduced slope of fEPSP in rapamycin‐treated CCH rats relative to controls (Figure S11F,G, Supporting Information). Behaviorally, rapamycin treatment led to impaired learning and memory in the Barnes maze, as evidenced by prolonged escape latency during the learning phase (Figure S11H, Supporting Information), fewer target hole contacts, and reduced time spent in the target quadrant during the test phase (Figure S11I,J, Supporting Information).

**Figure 9:**
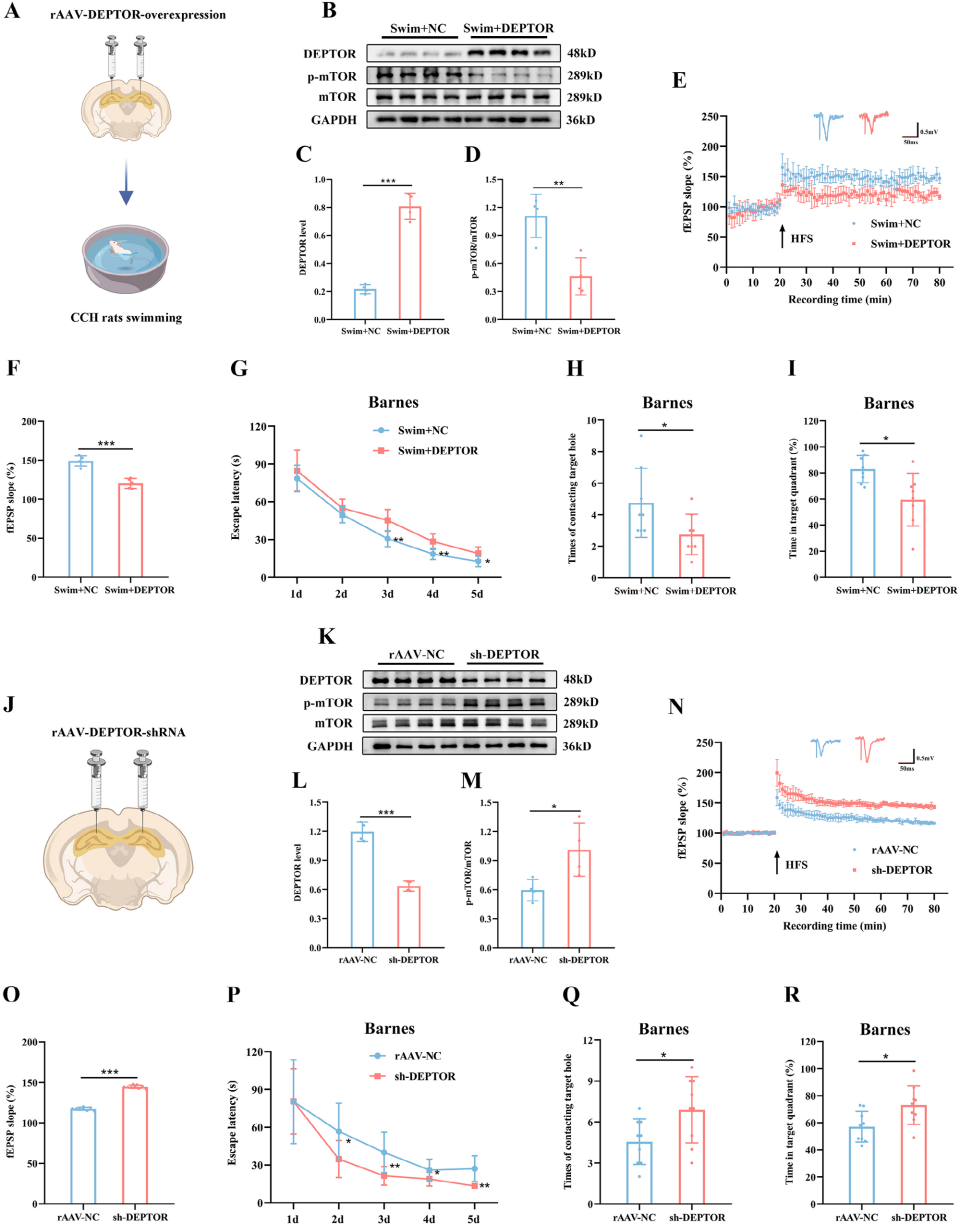
mTOR pathway plays a critical role in the cognitive improvement in CCH rats. A) Schematic diagram depicting rats receiving rAAV injection in the hippocampus and swimming training. B) Representative western blots of DEPTOR and mTOR in the hippocampus treated with rAAV‐DEPTOR‐overexpression. C,D) Quantitative analysis of DEPTOR (C) and p‐mTOR/mTOR (D) in the hippocampus. E) Time‐plot of normalized fEPSP slopes recorded from hippocampal slices after rAAV injection in the hippocampus. Representative traces of fEPSPs in the Swim+NC and Swim+DEPTOR groups. F) Histogram representing the average normalized fEPSP slope during the last 10 min of recording following HFS, represented as a percentage of the baselines for each group. G) Latency to reach the target hole in the learning stage of the Barnes maze after rAAV injection in the hippocampus. H,I) Number of contacts with the target hole (H) and duration percentage in the target quadrant (I) during the probe trial after rAAV injection in the hippocampus. J) Schematic diagram depicting rats receiving rAAV injection in the hippocampus. K) Representative western blots of DEPTOR and mTOR in the hippocampus treated with rAAV‐shRNA‐DEPTOR. L,M) Quantitative analysis of DEPTOR (L) and p‐mTOR/mTOR (M) in the hippocampus. N) Time‐plot of normalized fEPSP slopes recorded from hippocampal slices after rAAV injection in the hippocampus. Representative traces of fEPSPs in the rAAV‐NC and sh‐DEPTOR groups. O) Histogram representing the average normalized fEPSP slope during the last 10 min of recording following HFS, represented as a percentage of the baselines for each group. P) Latency to reach the target hole in the learning stage of the Barnes maze after rAAV injection in the hippocampus. Q,R) Number of contacts with the target hole (Q) and duration percentage in the target quadrant (R) during the probe trial after rAAV injection in the hippocampus. Data are presented as the mean ± SD. *n* = 4 per group (B–D and K–M). *n* = 5 per group (E and F). *n* = 8 per group (G–I). rAAV‐NC group *n* = 8, sh‐DEPTOR group *n* = 9 (N and O). *n* = 9 per group (P–R). ^*^ *p* < 0.05, ^**^ *p* < 0.01, ^***^ *p* < 0.001. Statistical analysis was performed using unpaired two‐tailed Student's *t*‐test (C, D, F, H, I, L, M, O, Q, and R), and two‐way ANOVA for repeated measures (G and P). ANOVA, Analysis of variance; DEPTOR, DEP‐domain containing mTOR‐interacting protein; fEPSP, Field excitatory postsynaptic potential; HFS, High frequency stimulation; mTOR, Mechanistic target of rapamycin; rAAV, Recombination adeno‐associated virus; SD, Standard deviation.

To further explore the role of hippocampus‐specific mTOR regulation in exercise‐induced cognitive improvement, we employed genetic knock‐in of DEPTOR, a potent upstream inhibitor of the mTOR pathway^[^ ^58^ ^]^ (**Figure** **9**A; Figure S11K, Supporting Information). Overexpression of DEPTOR significantly inhibited mTOR phosphorylation in exercise‐trained rats (Figure 9B–D). Consistent with this, measurements of field potential in the hippocampus revealed a decreased slope of fEPSP in CCH rats overexpressing DEPTOR (Figure 9E,F). In behavioral assessments, hippocampus‐specific mTOR inhibition also impaired cognitive function in exercise‐trained rats (Figure 9G–I; Figure S11L,M, Supporting Information). Conversely, to investigate the effects of genetic knockdown of DEPTOR, we injected shRNA into the hippocampus (Figure 9J; Figure S11N, Supporting Information). This intervention significantly induced mTOR phosphorylation in the hippocampus (Figure 9K–M), which in turn enhanced synaptic plasticity (Figure 9N,O) and alleviated cognitive dysfunction (Figure 9P–R; Figure S11O,P, Supporting Information) in CCH rats, mimicking the cognitive benefits seen with swimming training. Together, these results suggest that exercise‐induced brain mTOR activation is essential for enhancing synaptic plasticity and cognitive function in CCH rats.

**Figure 10:**
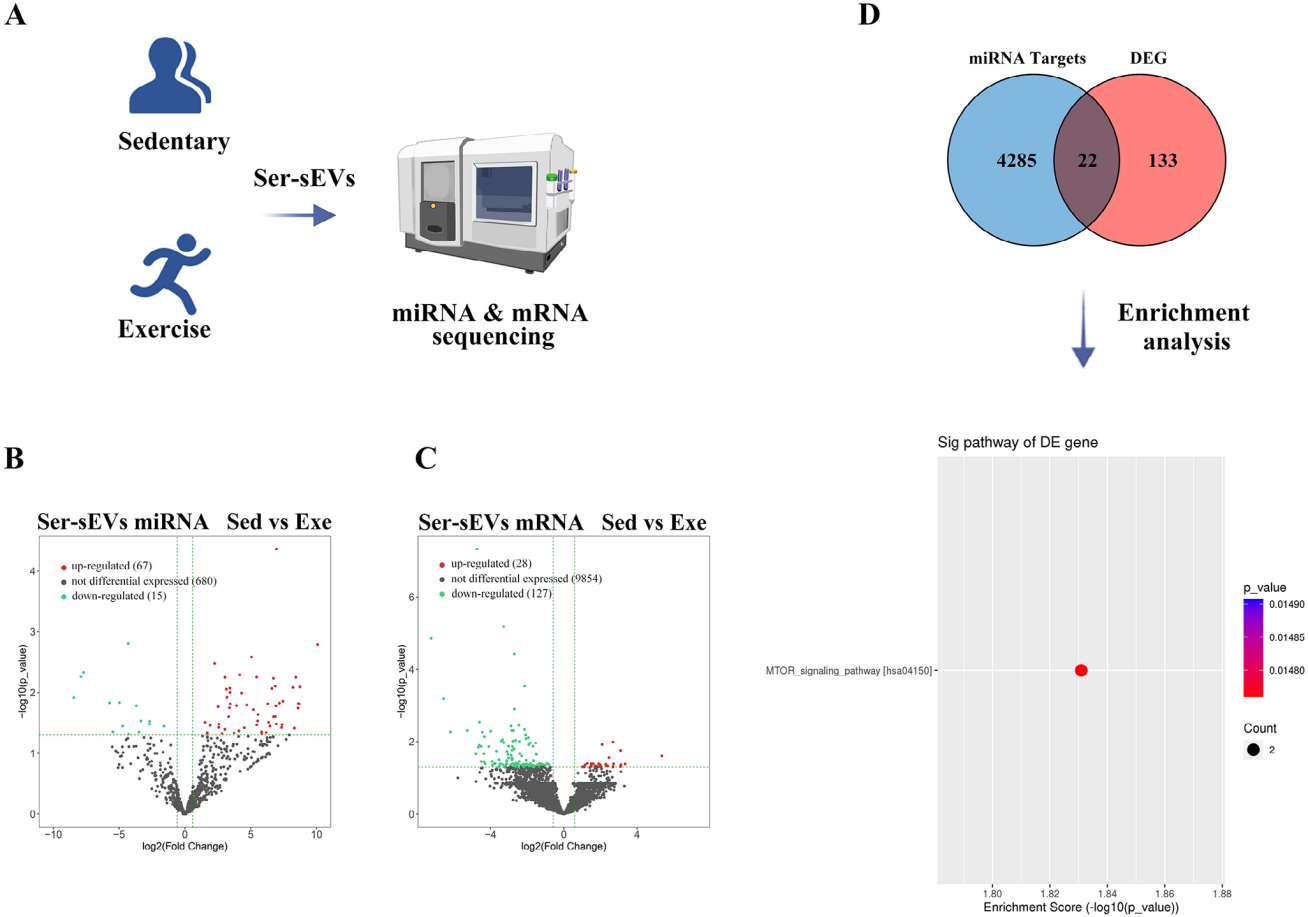
mTOR pathway participates in adaptive changes in response to long‐term regular exercise in humans. A) Schematic diagram for a small‐cohort clinical study of sEVs sequencing in sedentary and long‐term exercise individuals. B) Volcano map presentation of relative miRNA differences in sEVs of sedentary and long‐term exercise individuals. C) Volcano map presentation of relative mRNA differences in sEVs of sedentary and long‐term exercise individuals. D) Venn diagram of target genes of differentially expressed miRNAs and differentially expressed mRNAs between sedentary and long‐term exercise individuals. Signaling pathway enrichment analysis of the overlap miRNAs‐mRNA in the sEVs is shown. *n* = 5 per group (5 mixed samples from 25 subjects) (B and C). DEG, Differentially expressed genes; mTOR, Mechanistic target of rapamycin; sEVs, Small extracellular vesicles.

To assess whether these findings are applicable to humans, we recruited 25 individuals with a history of long‐term regular exercise and 25 age‐ and sex‐matched controls who did not engage in regular exercise (Table S4, Supporting Information). We isolated peripheral sEVs from both groups and conducted high‐throughput sequencing to analyze miRNAs and mRNAs (**Figure** **10**A). This analysis revealed 82 differentially expressed miRNAs between the exercise and control groups (fold change >1.5; *P*<0.05; Figure 10B), with 67 miRNAs upregulated and 15 downregulated in the exercise group. To confirm these findings, we conducted qPCR on eight differentially expressed miRNAs in the isolated sEVs, which validated the results from high‐throughput sequencing (Figure S11Q and Table S5, Supporting Information). Additionally, we identified 155 differentially expressed genes between the exercise and control groups (fold change >1.5; *P*<0.05; Figure 10C). Among these, 28 mRNAs were upregulated, while 127 were downregulated in the exercise group. By integrating the miRNA and mRNA data, we identified miR‐130a‐3p–CAP‐Gly domain‐containing linker protein 1 (CLIP1) and miR‐1908‐5p–serine/threonine‐protein kinase 11 (STK11) as potential miRNA–mRNA regulatory pairs. Both miR‐130a‐3p–CLIP1 and miR‐1908‐5p–STK11 are known to influence the mTOR pathway (Figure 10D), which aligns with our observations in rodent models. Collectively, these results highlight the mTOR pathway as a key target of exerkines, suggesting its role in mediating the protective effects of exercise on brain structure, function, and cognition.

## 3 Discussion

Our study demonstrates that swimming training triggers the secretion of signaling molecules from muscle‐derived sEVs, which travel to the brain and enhance cognition by improving synaptic plasticity in a CCH rodent model. Notably, sEVs isolated from rats undergoing long‐term swimming training replicated the cognitive benefits of exercise in sedentary CCH rats. Mechanistically, we identified miR‐17/20a‐5p as an exercise‐induced exerkine that is upregulated in response to swimming. This miRNA, carried by circulating sEVs, reaches the hippocampus, where it suppresses DEPTOR expression, thereby activating the mTOR pathway. This activation mitigates synaptic dysfunction and rescues cognitive impairments caused by CCH. Our findings also confirmed that muscle‐derived sEVs are the primary source of miR‐17/20a‐5p in circulating sEVs after swimming, revealing a distinct molecular pathway through which peripheral muscle activity directly influences brain structure, function, and cognition. Further studies in CCH rats subjected to treadmill exercise revealed different miRNA cargos in sEVs, which also regulated mTOR activation in the hippocampus. These results suggest that mTOR pathway regulation in the brain represents a shared mechanism by which exerkines mediate their cognitive benefits.

VCI, a common cognitive disorder in older adults, is predominantly caused by cerebrovascular diseases and vascular risk factors. VCI encompasses a spectrum, ranging from subjective cognitive decline and mild cognitive impairment to dementia.^[^ ^59^ ^]^ Although disease‐modifying pharmacological treatments for VCI remain limited, lifestyle modifications, particularly physical exercise, are increasingly recognized as promising strategies for its treatment and prevention. Exercise has consistently been associated with improved memory and executive function^[^ ^6^, ^60^ ^]^ and is emerging as a powerful non‐pharmacological approach to maintaining and enhancing brain function.^[^ ^61^ ^]^

Exercise stimulates the upregulation of brain‐derived neurotrophic factor (BDNF) in the hippocampus, a key molecule essential for adult hippocampal neurogenesis and synaptic plasticity.^[^ ^11^ ^]^ Substantial evidence also supports the idea that exercise exerts systemic benefits, with peripheral factors mediating its effects on the brain.^[^ ^62^ ^]^ Numerous molecules released from various tissues during exercise enter the circulation and reach the brain. These include peroxisome proliferator‐activated receptor gamma coactivator 1‐alpha (PGC‐1α),^[^ ^63^ ^]^ cathepsin B (CTSB),^[^ ^64^ ^]^ irisin,^[^ ^13^ ^]^ and lactate from muscle;^[^ ^65^ ^]^ glycosylphosphatidylinositol‐specific phospholipase D1 (GPLD1) from the liver^[^ ^12^ ^]^; adiponectin from adipocytes;^[^ ^66^ ^]^ and platelet factor 4,^[^ ^67^ ^]^ clusterin,^[^ ^68^ ^]^ and selenoprotein P  from blood.^[^ ^69^ ^]^ In the brain, these secretory factors play critical roles in promoting synaptic plasticity, enhancing neurogenesis, and modulating neuroinflammation, which collectively regulate memory and mood. These bioactive molecules are collectively referred to as exerkines, a term encompassing all humoral factors (including miRNA, mRNA, and proteins) secreted into circulation by tissues in response to exercise. Exerkines are often packaged into extracellular vesicles, which facilitate their systemic actions.^[^ ^70^ ^]^

sEVs are membrane‐derived particles released by various cell types into the extracellular space, playing critical roles in organ‐to‐organ communication. One study found that exercise triggers a rapid release of sEVs into the circulation, with this release initiated during the aerobic phase of exercise.^[^ ^18^ ^]^ In contrast, other research demonstrated that a single session of high‐intensity interval exercise does not alter endothelial or platelet microparticle levels.^[^ ^71^ ^]^ This controversy extends to the effects of chronic exercise on sEVs as well, with conflicting findings reported in multiple studies.^[^ ^32^, ^33^, ^72^ ^]^ One study indicated that exercise regulates the cargo of sEVs, and that exercise‐induced sEVs exhibit distinct distribution patterns and tissue‐specific propensities. This suggests that exercise exerts systemic biological effects through inter‐tissue signaling proteins.^[^ ^19^ ^]^ Similarly, another study showed that while 4 weeks of swimming training in rats did not significantly affect the concentration of peripheral sEVs, it did alter the composition of the sEV cargos.^[^ ^33^ ^]^ In our study, we demonstrated that swimming training significantly promoted the secretion of muscle‐derived sEVs into the circulation, which then directly targeted the brain to enhance synaptic function and alleviate cognitive deficits. Systemic treatment with an inhibitor of sEV biogenesis and muscle‐specific inhibition of sEV release both reduced these effects, underscoring the crucial role of muscle‐derived sEVs in mediating exercise‐induced brain protection. These findings align with previous research.^[^ ^73^, ^74^ ^]^

sEVs are increasingly recognized as promising drug delivery vehicles, particularly for neurological applications, due to their ability to cross the blood–brain barrier and deliver therapeutics. In our study, we demonstrated that sEVs isolated from the serum and skeletal muscle of exercised rats replicated the cognitive benefits of exercise in unexercised rats. These benefits included enhanced synaptic plasticity and the reversal of cognitive impairments caused by CCH. Similarly, another study showed that brain‐derived sEVs from exercised rats promoted synaptic growth and corticospinal tract integrity in unexercised animals, facilitating neurological recovery and improved gait in an acute cerebral ischemia model.^[^ ^75^ ^]^

Additionally, sEVs are thought to serve as carriers for exerkines, traveling through the body to deliver their cargo to distant tissues. A growing body of evidence shows that various cell‐derived miRNAs play therapeutic roles in cerebral ischemia.^[^ ^76^ ^]^ For example, neuro‐derived miR‐132,^[^ ^77^ ^]^ microglia‐derived miR‐124,^[^ ^78^ ^]^ brain endothelial cell‐derived miR‐126,^[^ ^79^ ^]^ and mesenchymal stem cell (MSC)‐derived miR‐133b^[^ ^80^ ^]^ have all been implicated in neuroprotection. Furthermore, miR‐126‐enriched endothelial progenitor cell‐derived sEVs were shown to mediate the protective effects of exercise on ischemic injury.^[^ ^35^ ^]^ In our study, we identified miR‐17/20a‐5p as an enriched exerkine in sEVs and the hippocampus of rats following swimming training. We demonstrated that inhibiting miR‐17/20a‐5p reduced the beneficial effects of swimming on synaptic plasticity and cognitive function. Both miR‐17‐5p and miR‐20a‐5p belong to the miR‐17‐92 cluster, which is located on the same chromosome (chromosome 13) and shares an identical seed sequence. This cluster has been extensively studied and shown to regulate developmental neurogenesis,^[^ ^81^ ^]^ adult neurogenesis,^[^ ^82^, ^83^ ^]^ and angiogenesis.^[^ ^84^, ^85^ ^]^ Recently, MSC‐derived sEVs enriched in the miR‐17‐92 cluster were reported to enhance neuroplasticity by promoting axon‐myelin remodeling and motor electrophysiological recovery in rats with acute brain ischemia.^[^ ^24^, ^86^ ^]^ In our study, we demonstrated that among the miRNAs in this cluster, miR‐17/20a‐5p specifically mediated the swimming‐induced improvements in synaptic plasticity and cognition in CCH rats.

KEGG analysis of differentially expressed miRNAs following swimming training identified the mTOR pathway as a likely target of miR‐17/20a‐5p, which was upregulated in sEVs. Further investigation revealed that miR‐17/20a‐5p directly interacts with DEPTOR, a well‐known upstream negative regulator of mTOR complexes that plays critical roles in cell proliferation and survival.^[^ ^58^ ^]^ Inhibiting miR‐17/20a‐5p increased DEPTOR levels in the hippocampus, which led to reduced mTOR activation, enhanced p70S6K activity, and decreased 4EBP1 activity. These findings support a model in which sEVs enriched in miR‐17/20a‐5p alleviate CCH‐induced synaptic impairment and cognitive dysfunction by activating the mTOR pathway through DEPTOR suppression. Consistently, hippocampus‐specific overexpression of miR‐17/20a‐5p was sufficient to rescue the synaptic and cognitive deficits caused by CCH.

To enhance targeted delivery of sEV cargos to the brain in vivo, we engineered sEVs by fusing the neuron‐specific RVG peptide to LAMP2B, an sEV membrane protein, as previously described.^[^ ^54^ ^]^ We then loaded these RVG‐sEVs with miR‐17/20a‐5p and assessed the effects of intravenous delivery on synaptic plasticity and cognitive outcomes in CCH rats. Systemic administration of miR‐17/20a‐5p‐enriched RVG‐sEVs resulted in efficient hippocampal delivery of the cargo. Furthermore, the delivered miRNAs demonstrated biological activity, as shown by improvements in synaptic plasticity and cognition in treated CCH rats. These findings highlight the utility of engineered sEVs as a drug delivery platform for the brain and underscore the therapeutic potential of miR‐17/20a‐5p for treating CCH‐induced cognitive impairments.

We found that exercise significantly upregulated miR‐17/20a‐5p expression in the liver, muscle, and hippocampus. However, pre‐miR‐17/20a‐5p levels increased only in muscle, mirroring the pathway through which exercise promotes the release of irisin, the cleaved and N‐terminal portion of fibronectin type III domain containing 5 (FNDC5), from muscle to the brain.^[^ ^87^ ^]^ Our analysis of miRNA expression profiles in muscle‐derived sEVs revealed that they closely resembled those in skeletal muscle tissue, suggesting that sEVs serve as effective carriers of exerkines. This was further supported by our observation that muscle‐specific inhibition of sEV release attenuated the cognitive benefits induced by exercise.

Intramuscular administration of rAAV‐miR‐17/20a‐5p‐sponge in exercised CCH rats confirmed that muscle‐derived sEVs are the primary source of exercise‐induced miR‐17/20a‐5p in the hippocampus. This intervention led to decreased levels of miR‐17/20a‐5p in muscle‐derived sEVs, circulating sEVs, and the hippocampus. It also impaired synaptic plasticity and worsened behavioral test performance compared to control‐treated animals. Moreover, sEVs isolated from the skeletal muscle of exercised rats successfully mimicked the cognitive protective effects of exercise in CCH rats. These findings align with growing evidence that myokines secreted by muscle regulate brain function. For example, prior studies have demonstrated that myokines such as CTSB^[^ ^64^ ^]^ and irisin^[^ ^87^ ^]^ can cross the blood‐brain barrier to enhance brain‐derived neurotrophic factor production, thereby promoting neurogenesis. Studies have shown that IL‐6 suppresses central mechanisms of feeding,^[^ ^30^, ^88^ ^]^ while increases in PGC1α‐dependent muscular expression of kynurenine aminotransferase enzymes alleviate stress‐induced depression.^[^ ^63^ ^]^ In addition, many studies referred to miR‐1, miR‐206 and miR‐133 as “myomiRs”, noting their high expression levels in skeletal muscle.^[^ ^89^ ^]^ However, there is limited research on the mechanisms by which exercise induces muscle‐derived miRNAs to regulate the brain. We have expanded this understanding and identified novel muscle‐derived miRNAs that are secreted in an exercise‐driven manner.

Together, these findings highlight the significant role of exocrine signaling from muscle to brain in modulating brain function, with muscle‐derived sEVs serving as key mediators of muscle‐brain communication. Our study further demonstrated that inhibiting miR‐17/20a‐5p partially diminished the cognitive benefits of exercise. However, CCH rats receiving virus injections still exhibited better cognitive performance than untreated control CCH rats. Similarly, supplementation with miR‐17/20a‐5p improved cognitive impairment caused by CCH but did not fully restore it to levels observed in the sham group. These findings suggest that while miR‐17/20a‐5p contributes to exercise‐induced cognitive improvements, other exerkines, such as lactate,^[^ ^65^ ^]^ irisin,^[^ ^13^ ^]^ and CTSB^[^ ^64^ ^]^ also play crucial roles. Moreover, miR‐17/20a‐5p may indirectly influence some of these factors, indicating that the muscle‐brain axis involves complex interactions among multiple signaling molecules. No single factor appears sufficient to account for the comprehensive neuroprotective and cognitive benefits induced by exercise. In conclusion, miR‐17/20a‐5p likely serves as an important regulatory molecule within the broader, multifactorial processes of the muscle‐brain axis. However, it is only one component of a complex network of exerkines driving exercise‐induced neuroprotection and cognitive enhancement.

Interestingly, CCH did not induce significant changes in miR‐17/20a‐5p levels in muscle, whereas exercise increased miR‐17/20a‐5p levels compared to unexercised rats. This observation led us to further explore the relationship between miR‐17/20a‐5p and exercise in healthy rats to better understand the effects of CCH on muscle miRNA profiles. To this end, we included an independent cohort of healthy SD rats that underwent 4 weeks of swimming training. We measured miR‐17/20a‐5p levels in both the skeletal muscle and hippocampus. Compared to the sedentary group, swimming training significantly upregulated miR‐17/20a‐5p levels in both tissues. Correlation analysis revealed a positive association between miR‐17‐5p levels in muscle and hippocampus with cognitive performance, as assessed by the Y maze and novel object recognition (NOR) tests. These results suggest that CCH has minimal impact on the miRNA expression profile in muscle‐derived sEVs, while physiological adaptations in muscle in response to exercise training play a key role in improving cognitive function, independent of brain injury.

We also investigated the effects of treadmill exercise in the CCH model and observed increased dendritic spine density and enhanced LTP, consistent with previous studies.^[^ ^90^, ^91^, ^92^ ^]^ However, we found that treadmill training did not increase miR‐17/20a‐5p levels in the hippocampus. Instead, we detected elevated levels of miR‐144 and identified PTEN as its likely molecular target.^[^ ^93^ ^]^ When we inhibited miR‐144‐3p by injecting AAV‐miR‐144‐3p‐sponge into the hippocampus of exercised rats, we observed a decrease in PTEN levels and activation of mTOR signaling. Notably, inhibiting miR‐144‐3p eliminated the treadmill exercise‐induced benefits on synaptic plasticity and cognitive function in CCH rats.

Our results suggest that mTOR serves as a common target for exerkines mediating neuroprotection, consistent with previous findings demonstrating that mTOR signaling is responsive to exercise.^[^ ^94^ ^]^ A prior study showed that exercise enhanced motor skill learning through selective activation of mTOR in the mouse motor cortex, which correlated with increased oligodendrogenesis and axonal myelination.^[^ ^95^ ^]^ mTOR is increasingly recognized as a key mediator of exercise‐induced brain protection. Specifically, exercise activates mTOR‐pS6 to restore synaptic function following cocaine exposure^[^ ^96^ ^]^ and induces FMRP‐mTOR signaling to support cortical neural activity, thereby improving stress resilience.^[^ ^97^ ^]^ In agreement with these findings, we observed that systemic rapamycin treatment abolished the exercise‐induced improvements in synaptic plasticity and cognitive function. Furthermore, hippocampus‐specific overexpression of DEPTOR similarly impaired the beneficial effect of exercise and hippocampus‐specific activation of the mTOR pathway alone was sufficient to mimic the effects of exercise on brain structure, function, and cognition. Previous studies have also shown that mTOR activation promotes synaptic protein synthesis^[^ ^98^ ^]^ and enhances synaptic transmission.^[^ ^99^ ^]^ Notably, when comparing sEVs isolated from individuals after long‐term exercise with those from sedentary controls, we identified differential expression of two miRNA–mRNA pairs that regulate mTOR activation. These findings highlight specific miRNAs, regulated by exercise in both rodents and humans, that target the mTOR pathway. Functional studies in rodents further demonstrate that these miRNAs act as exocrine signals, conveying exercise‐induced benefits from muscle to brain.

Our study has several limitations that should be considered when interpreting the findings. First, we did not investigate the spatial expression patterns of miRNAs in the brain. Additionally, although our results suggest that muscle‐derived sEVs are a key—likely primary—source of miR‐17/20a‐5p‐enriched sEVs following exercise, we did not comprehensively examine the potential effects of circulating sEVs from other organs on the brain. Another limitation is that we only evaluated two types of aerobic exercise; we did not assess the effects of other aerobic modalities or resistance training. Previous studies have shown correlations between the miRNA content in sEVs and exercise intensity, suggesting that intensity may influence the packaging and release of sEVs.^[^ ^100^ ^]^ Moreover, variability in the release of miRNAs into circulation has been observed when participants engaged in different types of resistance training.^[^ ^101^ ^]^ These findings indicate that both exercise mode and intensity likely play significant roles in the release of sEVs into circulation. Therefore, further research is needed to explore the peripheral‐central mechanisms underlying the role of muscle‐derived sEVs in treadmill‐induced cognitive protection. Additionally, including voluntary exercise in future studies could enhance the generalizability of our findings to broader physical activity contexts. Finally, our study did not address the significance of modulating mTOR activity in response to different exercise regimens, nor did it explore how mTOR regulation may function in pathological conditions beyond CCH.

Our results reveal a novel mechanism of muscle‐to‐brain communication that mediates exercise‐induced cognitive improvements in CCH. We demonstrate that miR‐17/20a‐5p, encapsulated in muscle‐derived sEVs, can transfer to the brain to activate mTOR signaling. This mechanism enhances our understanding of how exercise induces exocrine signaling to promote beneficial effects on the central nervous system, highlighting the therapeutic potential of myokines in treating cognitive impairment. Cross‐species analysis underscores the central role of mTOR signaling in activating molecular programs that protect brain health in response to exercise.

## 4 Experimental Section

### Animals

Sprague‐Dawley (SD) rats, weighing 280±20 g, were obtained from Slack Laboratory Animal (Shanghai, China) and used for this study. The rats were bred at the Experimental Animal Center of Fujian University of Traditional Chinese Medicine (Permit number: SCXK (Min) 2020‐0002). They were housed in a temperature‐controlled environment (22–26 °C) with a 12‐h light/dark cycle and a humidity level of 40–50%. The Animal Experiment Ethics Committee of Fujian University of Traditional Chinese Medicine approved all experimental procedures involving animals (Ethics number: FJTCM IACUC 2020100).

### Participants

For circulating serum sEVs miRNA and RNA sequencing analysis, we randomly recruited 50 healthy male participants from the Second People's Hospital Affiliated to Fujian University of Traditional Chinese Medicine. Eligible participants were 28–49 years old individuals with BMI<30 and cognitively normal by report. Exclusion criteria included any history of significant general medical disease, neurological disease of the central nervous system, psychiatric disorder (other than mild depression), type 2 diabetes and hypoglycemia. Participants were fully informed about the procedures and risks, and written informed consent was provided prior to any study procedures. The screening visit included a history of physical activity, weight, height, blood pressure, smoking and drinking and a blood draw for sEVs isolation. The cohort included 25 individuals who had engaged in regular exercise for an extended period and 25 sedentary, age‐ and sex‐matched controls. Serum samples were collected using BD Vacutainer tubes and stored at −80 °C in cryopreservation tubes until analysis. Due to the limited serum volume per individual, samples were pooled into groups of five, resulting in five samples per group for subsequent analysis. Sedentary control subjects were defined as individuals with no regular physical activity for more than five years. Long‐term regular exercise was defined as consistent physical activity for at least five years, with a frequency of three or more sessions per week, each lasting 30–90 min. To validate the miRNA sequencing results, we recruited an additional cohort of 46 healthy men aged 30–50 years, comprising 22 sedentary controls and 24 long‐term regular exercise participants. The research adhered to the principles of the Declaration of Helsinki and received approval from the Ethics Committee of the Second People's Hospital Affiliated to Fujian University of Traditional Chinese Medicine (Ethics number: SPHF JP‐K2019056‐01). The study was registered with the Chinese Clinical Trial Registry (ChiCTR) (Registration number: ChiCTR2000032660).

### CCH Model Establishment

The CCH model was established, which induced vascular cognitive impairment, using the bilateral common carotid artery ligation method described previously.^[^ ^36^ ^]^ Rats were anesthetized with 3% isoflurane and placed on a surgical insulation pad. After exposing the trachea through a dissection of the subcutaneous tissue, the common carotid arteries were carefully separated on both sides of the trachea using micro forceps. The right common carotid artery was ligated first, followed by the left artery after a 5‐min interval. Control rats underwent the same procedure, except their bilateral common carotid arteries were isolated without ligation. The surgical wound was then sutured and cleaned. Lidocaine gel was applied to the wound for pain management. Rats were placed on a heating pad until awakening. The postoperative survival rate is 95%. After surgery, the blood flow and cognitive function of rats were tested, and rats with significant blood flow decline and cognitive deficits were used for subsequent experiments.

### Exercise Protocol

Swimming Training Protocol—Rats were trained to swim in a water environment following a progressively increasing schedule. The training began with 15 min per day during the first week, then increased to 20, 35, 40 min, and finally 60 min per day. The animals underwent 60 min of swimming training per day, 5 d per week, for a total of 3 weeks.

Treadmill Training Protocol—Rats were acclimated to the treadmill over 3 consecutive days before starting the exercise regimen. The training duration was gradually increased from 20 to 40 min, and eventually to 60 min per session. The animals were trained at a constant speed of 15 m min^−1^ on a 0° incline for 60 min per day, 5 d per week, over 4 weeks.

### Magnetic Resonance Angiography (MRA)

3D time‐of‐flight magnetic resonance angiography (3D‐TOF MRA) was performed using a 7.0T small animal MRI system (Bruker Biospec 70/20 USR, Germany). Rats were anesthetized with 3% isoflurane, and their heads were secured using a specialized dental and ear rod in the prone position. During the scanning process, anesthesia was maintained with 1.5% isoflurane, and body temperature was maintained at 36.5–37.5 °C using a water circuit. The scanning parameters were as follows: repetition time (TR) = 15 ms, echo time (TE) = 2.7 ms, field of view (FOV) = 30 × 30 × 24 mm, averages = 1, slices = 1, slice thickness = 24 mm, and total acquisition time = 5 min 5.280 s.

### Behavioral Testing

Barnes Maze Test—The Barnes maze was used to evaluate spatial learning and memory in rats.^[^ ^37^ ^]^ The apparatus consisted of a black circular platform, 150 cm in diameter, with 20 evenly spaced openings along the perimeter, each 15 cm in diameter. An escape box made of black organic material was attached to one of these openings, designated as the target hole. The test was conducted in two stages, as described below

Learning Stage: On the day prior to testing, rats were acclimated to the escape box, start box, and black platform for 2 min. During the learning stage, each rat was initially placed in the start box at the center of the platform for 15 s. Afterward, a sound stimulation device was activated, and the start box was removed, allowing the rat to explore freely for 120 s. If the rat located and entered the escape box, the trial was stopped. Each rat completed two learning trials per day over 5 consecutive days. Between trials, the maze and target hole were cleaned with 75% ethanol to prevent olfactory cues. Probe Phase: On the 6th day, the escape box was removed, and rats were allowed to explore the platform freely for 120 s. The SuperMaze system (Xinruan, Shanghai, China) tracked the rats' movements and recorded the following parameters: escape latency, number of contacts with the target hole, and the percentage of time spent in the target quadrant.

Morris Water Maze (MWM)**—**The Morris water maze was used to assess spatial learning and memory in rats.^[^ ^38^ ^]^ The maze consisted of a black circular pool with a radius of 80 cm and a depth of 50 cm, divided into four quadrants. A transparent circular escape platform was placed in the third quadrant. The experimental procedure included two phases

Orientation Navigation Experiment: On the day before testing, rats were acclimated to the water environment. During the orientation navigation phase, each rat was placed into the pool and allowed to freely navigate. The time taken to locate the escape platform was recorded as escape latency. If a rat failed to find the platform within 90 s, it was gently guided to the platform and allowed a 15‐s learning period before being dried with towels. This procedure was repeated four times per day for four consecutive days, with the rats being placed in different quadrants each time. Spatial Exploration Experiment: On the fifth day, the escape platform was removed. Rats were placed in the water from the quadrant opposite to the original platform location. Their movements were tracked for 90 s. The SuperMaze system (Xinruan, Shanghai, China) recorded swimming trajectories, escape latency, the number of platform crossings, and the time spent in the quadrant where the platform had previously been located.

Y Maze Test**—**The Y‐maze spontaneous alternation task was conducted to assess the working memory of rats.^[^ ^36^ ^]^ The Y‐maze consisted of three arms (labeled A, B, and C), each measuring 50 cm × 10 cm × 20 cm, with a 120° angle between them. At the beginning of the experiment, rats were placed in the maze and allowed to explore freely for 8 min. Each time a rat fully entered an arm, it was recorded as an entry. The sequence of arm entries was recorded during this period, and consecutive entries into all three arms were considered correct spontaneous alternations. The SuperMaze software (Xinruan, Shanghai, China) was used to track the order of arm entries and the total number of entries. The rate of correct spontaneous alternations was calculated as the number of correct alternations divided by the total number of entries minus two, then multiplied by 100%.

Novel Object Recognition (NOR)**—**The NOR test was conducted to evaluate novel object discrimination in rats.^[^ ^36^ ^]^ The experimental setup included a black square box measuring 70 × 70 × 70 cm and three solid objects: “A” and “a,” which were identical, and “B,” a novel object. The protocol consisted of three distinct phases: adaptation, learning, and testing.

Adaptation Phase: On the first day, rats were introduced to the empty box without objects and allowed to explore freely for 5 minutes. Learning Phase: Two identical objects (“A” and “a”) were placed in opposite corners of the box. The rats, positioned facing away from the objects at the start, were given 10 min to explore. Testing Phase: One hour after the learning phase, object “a” was replaced with the novel object “B.” The rats were reintroduced into the box to assess memory retrieval and allowed to explore for 5 min. SuperMaze software (Xinruan, Shanghai, China) was used to track the rats' movements and measure the time spent exploring each object. The recognition index, a measure of novel object discrimination, was calculated as the ratio of TimeB to the sum of TimeA and TimeB. This index quantified the rats' ability to distinguish the novel object.

Open Field Test (OFT)—The OFT was performed to assess anxiety‐related behavior in rats.^[^ ^39^ ^]^ The testing apparatus consisted of a square box (70 × 70 × 70 cm) with its floor divided into 16 equal squares by infrared beams. The central zone was defined by four smaller squares in the middle of the floor. At the start of the test, each rat was placed in the center of the chamber and allowed to explore freely for 5 min. A camera recorded their movement trajectories, and SuperMaze software (Xinruan, Shanghai, China) was used to analyze the data.

### Small Extracellular Vesicles Isolation

Serum sEVs—Blood samples were collected from both rats and humans for analysis. These samples were subjected to sequential centrifugation steps: first at 1900×g for 10 min at 4 °C, followed by 3000×g for 15 min at 4 °C to obtain serum. To remove cellular debris, gradient centrifugation was performed at 500×g for 10 min, 2000×g for 30 min, and finally 10 000×g for 30 min. The supernatant was then passed through a 0.22 µm filter and ultracentrifuged at 120 000×g for 105 min at 4 °C using the Sorvall WX+ Ultracentrifuge Series (Thermo Scientific, USA). This process isolated sEVs, which were subsequently resuspended in 100–200 µl of phosphate‐buffered saline (PBS) for further experimentation.

Muscle sEVs—The gastrocnemius muscle was dissected from the hindlimbs of rats and chopped it into small segments for processing. The muscle fragments were incubated with digestive enzymes, and the resulting mixture was filtered through a 70 µm membrane at 4 °C to remove debris. The filtered solution was diluted with PBS and subjected to sequential centrifugation steps: 300×g for 10 min at 4 °C, 2000×g for 10 min at 4 °C, and 10 000×g for 20 min at 4 °C. The supernatant was filtered through a 0.22 µm membrane and further centrifuged at 150 000×g for 120 min at 4 °C. The resulting pellet was resuspended in PBS and loaded into a size‐exclusion chromatography column (Echo Biotech, Beijing, China). Based on the stable column performance and precise pore size distribution, SEC can achieve separation of particles of different sizes. Once the liquid level reached the upper mesh plate, PBS was added incrementally while collecting the corresponding fractions into a 100 kDa ultrafiltration tube. The solution was concentrated by centrifuging at 4000×g for 1 min at 4 °C. Finally, the pellet was resuspended in 200 µL of PBS for further experimentation.^[^ ^40^, ^41^ ^]^

### sEVs Identification and Characterization

Transmission Electron Microscopy (TEM)—The isolated sEVs suspension was thoroughly mixed and diluted to appropriate concentrations. A small volume was applied to a carbon‐coated copper grid and incubated at room temperature for 5 min. After rinsing the grids with sterile‐filtered PBS, the samples were stained with 3% uranyl acetate (UA) solution (GZ02625‐5, RXSV‐CHEM, China) for 1 min. Excess UA was carefully removed, and the grids were air‐dried for 15 min. The morphology of the sEVs was then examined and imaged using a TEM (H‐7650, Hitachi, Japan).

Nanoparticle Tracking Analysis (NTA)— The sEVs suspension was mixed thoroughly and diluted to suitable concentrations. The particle size distribution and concentration were analyzed using the ZetaView PMX 110 (Particle Metrix, Germany).

Western blotting (WB)—The protein concentration of the sEVs suspension was determined using a bicinchoninic acid (BCA) kit. Samples containing 30 µg of protein were separated by sodium dodecyl sulfate‐polyacrylamide gel electrophoresis (SDS‐PAGE) and transferred onto 0.22 µm polyvinylidene fluoride (PVDF) membranes. The membranes were blocked with 5% bovine serum albumin (BSA) and incubated overnight at 4 °C with primary antibodies. After washing with tris‐buffered saline containing Tween 20 (TBST), the membranes were incubated with secondary antibodies at room temperature for 1 h. The signals were visualized using an enhanced chemiluminescence (ECL) substrate and detected with a chemiluminescence imaging system (ChemiDoc MP, Bio‐Rad, USA). A complete list of antibodies used is provided in Table S1 in the Supporting Information.

Nano‐Flow Cytometer (NanoFCM)—The percentage was analyzed of muscle‐derived sEVs labeled with EGFP using a NanoFCM. The sEVs were diluted in PBS, and measurements were performed with a NanoAnalyzer equipped with dual laser sources (488 and 638 nm) and three single‐photon counting modules (SPCM) capable of simultaneous three‐channel detection. Samples and blanks were analyzed for 1 minute under a constant pressure of 1 kPa. Data were processed using NanoFCM Professional Suite v1.8 software.

### sEVs Trafficking Detection

PKH26—The PKH26 Fluorescent Cell Linker Kit (Sigma‐Aldrich, USA) was used to label the isolated sEVs. The sEVs was diluted in 2 mL of Diluent C and added 4 µL of PKH26 dye. After incubating the mixture at room temperature for 3–5 min, staining was halted by adding an equal volume of 0.5% BSA. The mixture was ultracentrifuged at 120 000×g for 70 min at 4 °C, and the resulting pellet was resuspended in 100–200 µL of PBS.

FITC**—**The ExoCoupl exosomes surface modification bioorthogonal coupling assay kit (Echo Biotech, China) was used to label the isolated sEVs. We diluted the sEVs in 500 µL of PBS and added 15 µL of Bioorthogonal Chemistry Reaction Solution. The sample was added to the column and ultracentrifuged at 80 rpm for 30 min at room temperature. Then, the column was placed in a new 2 mL EP tube and centrifuged at 500×g for 30 s to collect the effluent. 30 µL of N3‐FITC control was added to the purified effluent, and the mixture was incubated at room temperature in the dark for 2 h. Finally, the mixture was added to a new column for purification to remove free N3‐FITC control.

The labeled sEVs were injected via the tail vein, DAPI was used for counterstaining of nuclei (blue), and fluorescent signal in the brain was visualized using a fluorescence microscope (Leica DMi8, Germany) and a confocal microscope (Zeiss LSM 980, Germany).

### Engineered sEVs Preparation

Plasmid Construction and Transfection—The rabies virus glycoprotein (RVG)‐flag‐lamp2b sequence was synthesized by Echo Biotech (Beijing, China) and cloned into the pIRES‐zeocin‐cmv vector, generating the plasmid pIRES‐zeocin‐cmv‐RVG‐flag‐lamp2b (pIRES‐RVG‐flag‐lamp2b). HEK293F cells were cultured at 37 °C until achieving 95% viability. The cells were then co‐transfected with the pIRES‐RVG‐flag‐lamp2b plasmid using 4D‐Nucleofector transfection reagents and equipment (DP‐HZR‐001, Lonza, Switzerland). Post‐transfection, the cells were selected using zeocin and further cultured. The supernatant from these cultures was collected for sEVs isolation.

Loading of miRNA Mimics into sEVs—The miRNA mimics were loaded in sEVs through electroporation, a process carried out by Echo biotech (Beijing, China). Following sEVs isolation, the sEVs were combined with miRNA mimics in an electroporation buffer. Electroporation was performed using the Gene Pulser Xcell (Bio‐Rad, USA) at 250 V and 125 µF, with the process repeated ten times at 2‐s intervals. After electroporation, mixtures were concentrated into a final volume of 250 µL using Amicon Ultra‐15 Centrifugal Filter Units and subsequently washed three times with PBS to remove unincorporated material. The efficiency of miRNA loading was evaluated by absolute quantitative real‐time polymerase chain reaction. Briefly, a standard curve was generated using plasmid DNA at varying concentrations, different concentrations of the plasmid were used to plot, enabling for precise calculation of cycle threshold (*C*_t_) values. This linear relationship was used to estimate the copy number of miRNA in the engineered RVG‐sEVs, providing quantitative insights into the overexpression achieved.

### Stereotactic Injection of Virus

Plasmids and AAV vectors were provided by BrianVTA, Brain Case, and Obio Technology. First, pAAV MCS transfer plasmids containing ITR were constructed, which was driven by the strong promoter CMV, expressing EGFP and miRNA‐sponge. Then, miRNA‐sponge with 4–8 tandem complementary binding sites to the target miRNA were designed, and the AAV transfer plasmid and miRNA‐sponge insert were digested with selected restriction enzymes. Colony PCR or restriction digest were performed to confirm insertion. The AAV was prepared using triple‐plasmid transfection of HEK293 cells. 293T cells were cotransfected with the AAV transfer plasmids (containing miRNA‐sponge construct), pAAV helper plasmid and pAAV Rep/Cap plasmid. The cells were replenished with fresh medium 6 h post‐transfection and were collected 72 h post‐transfection to isolate AAV vectors.

Rats were anesthetized with 3% isoflurane and secured in a stereotactic frame (RWD, Shenzhen, China) for injections as previously described.^[^ ^42^ ^]^ After making a scalp incision and sterilizing the area, the periosteum was removed. A small hole was drilled above the hippocampus at the following coordinates: Anterior‐Posterior (AP): −3.5 mm, Medial‐Lateral (ML): ±2.0 mm, Dorsal‐Ventral (DV): −2.8 mm. A viral solution, with a titer of approximately 5 × 10¹^2^ vector genomes per mL, was injected at each site using a microsyringe pump at a rate of 50 nL min^−1^. A volume of 500 nL was delivered per site, and the micropipette was left in place for 10 min before retraction to minimize reflux. Control rats received injections of an empty adenovirus vector. The complete list of viral vectors used is detailed in Table S2 in the Supporting Information.

### sEVs Treatment

Rats subjected to CCH received weekly tail vein injections of swimming‐derived serum or muscle sEVs, each dissolved in 200 µL of saline (100 µg of total sEV protein per rat), starting 14 days after the induction of CCH and continuing for 4 weeks.^[^ ^24^ ^]^ Control rats were administered an equivalent volume of saline. Additionally, engineered sEVs (100 µg dissolved in 200 µL of saline) were injected via the tail vein once a week for 2 weeks.

### In Situ Intramuscular Injection

To label muscle‐derived sEVs, inhibit their secretion, and knock down skeletal muscle miR‐17/20a‐5p expression, we used AAV9 vectors driven by the synthetic muscle‐specific MHCK7 promoter and the CMV promoter. Rats were anesthetized with 3% isoflurane and positioned securely in a fixed apparatus. The bilateral gastrocnemius muscles were sterilized with alcohol, and the skin was fully exposed. Recombinant adeno‐associated virus (rAAV) was injected into the gastrocnemius muscle at each site, with a total dose of 1.25 × 10¹¹ vector genomes per site. The viral solution, prepared at a titer of ≈5 × 10¹^2^ vector genomes per mL, was diluted in PBS, and 25 µL was delivered at multiple injection points using a microsyringe. Control rats were injected with a negative control vector. A detailed list of the viral vectors used is provided in Table S2 in the Supporting Information.

### Rapamycin Administration

Rapamycin (HY‐10219, MCE, USA) was dissolved in ethanol and stored at −20 °C. Before injection, the stock solution was diluted with a mixture of polyethylene glycol 300 (PEG300) and Tween 80 in water. Rats received intraperitoneal injections of rapamycin at a dose of 2.0 mg k^−1^g,^[^ ^43^, ^44^ ^]^ administered one hour prior to the exercise intervention. This treatment was repeated five times per week for four weeks. Control animals were injected with an equal volume of the drug vehicle.

### GW4869 Administration

GW4869 (HY‐19363, MCE, USA) was dissolved in dimethyl sulfoxide (DMSO) and stored at −80 °C. Before administration, the stock solution was diluted with saline. Rats were intraperitoneally injected with GW4869 at a dose of 1.25 mg k^−1^g,^[^ ^45^ ^]^ one hour before the exercise intervention. This treatment was repeated three times per week for four weeks.^[^ ^46^ ^]^ Control animals received intraperitoneal injections of an equivalent volume of saline.

### miRNA Sequencing and Analysis

Rats were anesthetized with 3% isoflurane and euthanized via cervical dislocation. The brains were rapidly removed without PFA perfusion, hippocampus was collected and total RNA was isolated using TRIzol reagent (15596026, Invitrogen, USA). RNA concentrations were measured using the NanoDrop One spectrophotometer (Thermo Scientific, USA). Total RNA was also extracted from rat and human serum sEVs as well as skeletal muscle sEVs using Qiazol Lysis Solution (217184, Qiagen, Germany). The miRNA sequencing and analysis were performed by AKSomics Biological (Shanghai, China). Libraries for sequencing were prepared using the Stranded miRNA‐Seq Library Prep Kit (KAPA, USA), and their quality was evaluated with the Agilent 2100 Bioanalyzer. Deep sequencing was conducted using the Illumina NovaSeq 6000 platform. Sequencing reads were mapped to the Rattus norvegicus and human genomes. Differentially expressed genes and transcripts were identified using a negative binomial distribution model, with a fold change threshold greater than 1.5 and a *P*‐value less than 0.05.

### Dual‐Luciferase Reporter Assay

To investigate the direct interaction between miRNAs and their target genes, full‐length overlap primers were designed using the PCR‐based Accurate Synthesis (PAS) method. The wild‐type and mutant target gene sequences were synthesized, which were subsequently cloned into the psicheck2.0 vector, generating recombinant vectors. In vitro‐synthesized mimics of miR‐17‐5p, miR‐20a‐5p, and miR‐144‐3p were cotransfected with the recombinant vectors into 293T cells using the Lipo3000 lipofection method. The activities were then measured of both firefly and Renilla luciferases using the Duo‐LiteTM Luciferase Assay System (DD1205, Vazyme, Nanjing, China) to assess the regulation of target genes by these miRNAs.

### Quantitative Real‐Time Polymerase Chain Reaction (qPCR)

Tissues—Rats were anesthetized with 3% isoflurane and euthanized by cervical dislocation. The brains, heart, liver, spleen, lungs, kidneys, and gastrocnemius muscles were quickly collected to isolate total RNA using TRIzol reagent (15596026, Invitrogen, USA). RNA concentration was determined using the NanoDrop One (Thermo Scientific, USA). We reverse transcribed the isolated RNA into cDNA using the Mir‐X miRNA First‐Strand Synthesis Kit (638315, Takara, Japan) according to the manufacturer's instructions. miRNA expression was quantified with the Mir‐X miRNA qPCR TB Green Kit (638316, Takara, Japan) on a CFX Opus System (Bio‐Rad, USA). The miRNA levels were normalized to the endogenous control U6 and calculated the relative expression using the 2−ΔΔCt method. Primer details for all genes are listed in Table S3 in the Supporting Information. Raw sequencing data are available in the Gene Expression Omnibus (Accession Number: GSE205537).

:—Blood samples were collected from both rats and humans, and skeletal muscle tissues were harvested from rats. We isolated sEVs from rat serum and gastrocnemius muscle samples. Total RNA was extracted using Qiazol Lysis Solution (217184, Qiagen, Germany). The isolated RNA was reverse transcribed into cDNA using the miRCURY LNA RT Kit (339 340, Qiagen, Germany). miRNA expression in sEVs was quantified using the miRCURY LNA SYBR Green PCR Kit (339345, Qiagen, Germany) on a CFX Opus System (Bio‐Rad, USA). The miRNA levels were normalized to the exogenous control UniSp6 (339306‐YP00203954, Qiagen, Germany) and quantified using the 2−ΔΔCt method. Primer details for all genes are listed in Table S3.

### Immunohistochemical Staining

Rats were anesthetized with 2% pentobarbital sodium. Following the infusion of 0.9% saline and 4% paraformaldehyde (PFA), the brain tissues were fixed in 4% PFA for 24 hours and then embedded in paraffin. We obtained 5 µm‐thick coronal sections of the brain for further analysis. Immunohistochemical staining was performed using the instructions provided with the immunohistochemistry kit (MX Biotechnologies, Fuzhou, China). The tissue sections were deparaffinized with xylene and a gradient alcohol series, followed by heat‐induced antigen retrieval. After a brief rinse in PBS, the sections were blocked. Primary antibodies were applied overnight at 4 °C, followed by incubation with secondary antibodies. Diaminobenzidine (DAB) was used for chromogenic detection, and the average optical density values were quantified using ImageJ V1.8.0 (NIH, USA). A full list of the antibodies used is provided in Table S1 in the Supporting Information.

### Immunofluorescence Staining

Briefly, the brain sections were washed 2 × 15 min with PBS and were permeabilized for 15 min with 0.1% Triton X‐100, followed by 60 min blocking with 5% BSA. The brain slices were then incubated at 4 °C with the following primary antibody overnight: NeuN, GFAP and Iba‐1. Afterward, the brain slices were washed 3 × 5 min with PBS and incubated with appropriate secondary antibodies (Alexa Fluor 488/594 donkey anti‐rabbit IgG) for 1 h at room temperature. After 3 × 10 min with PBS, the brain slices were coverslipped DAPI and sealed with glycerol. A full list of the antibodies used is provided in Table S1 in the Supporting Information.

### Western Blotting

Proteins were extracted from rat hippocampi and serum‐derived sEVs by lysing samples in radioimmunoprecipitation assay (RIPA) buffer containing protease and phosphatase inhibitors. The protein concentration was determined using the BCA kit (Thermo Scientific, USA). Equal amounts of protein were loaded onto SDS‐PAGE gels and transferred to PVDF membranes. After washing with TBST, the membranes were blocked with 5% milk and incubated overnight with primary antibodies at 4 °C. The following day, the membranes were probed with secondary antibodies at room temperature. Protein bands were visualized using an imaging system (ChemiDoc MP, Bio‐Rad, Hercules, USA) and an ECL detection kit (S6009 M, US Everbright, USA). The gray intensity of the protein bands was quantified using ImageJ software. A complete list of the antibodies used is shown in Table S1 in the Supporting Information.

### Golgi Staining

Golgi staining was performed following the protocol outlined in the FD Rapid GolgiStain Kit (PK401, FD NeuroTechnologies, USA). Brain tissue was swiftly extracted and sectioned into 5–10 mm thick slices, which were then immersed in a mixture of solutions A and B for 14 d at room temperature, protected from light. After this incubation, the tissue was transferred to solution C and kept in the dark at room temperature for an additional 5 d. 100 µm thick frozen sections from the brain tissue were prepared and mounted them on slides coated with solution C. To stain the tissue, sections were immersed in a working solution composed of solutions D, E, and distilled water. After two rinses in distilled water, the sections were dehydrated through a graded ethanol series (50%, 75%, 95%, and 100%) and cleared using xylene. Finally, the sections were sealed with neutral resin. Tissue observation and imaging were conducted using an intelligent scanning system (Aperio VERSA, Leica, Germany).

### Electrophysiology

Rats were anesthetized with 3% isoflurane and euthanized through cervical dislocation. The brains were quickly removed and placed in artificial cerebrospinal fluid (ACSF) saturated with 95% oxygen and 5% carbon dioxide. The brain was then sectioned into 400 µm slices and incubated them at 37 °C for 30 min. These slices were transferred to the recording chamber, where a stimulating electrode was positioned on the fiber pathway from the Cornu Ammonis 3 (CA3) to the CA1 region, and the recording electrode was placed in the stratum radiatum of the CA1 region. Once the electrodes were in place, electrical stimulation, set to 30% of the maximum response amplitude, was applied to establish the baseline potential, which was recorded for 20 min. Following the baseline recording, two rounds of high‐frequency stimulation (100 Hz) with a 30‐s interval were delivered to induce LTP, and the potential was monitored for an additional 60 min.

### Statistical Analysis

All experimental data were analyzed using SPSS 25.0 software. Results are presented as mean ± standard deviation. To compare two groups, we applied an unpaired Student's *t*‐test. For comparisons across multiple groups, one‐way ANOVA was used, with pairwise comparisons conducted via the Bonferroni test. Additionally, two‐way repeated measures ANOVA were employed to assess data from multiple time points across groups. A *P*‐value of less than 0.05 was considered statistically significant.

## Conflict of Interest

The authors declare no competing interests.

## Author Contributions

H.L., L.Y., and W.L. contributed equally to this work. H.L., W.L., and L.C. conceived the study. H.L. and W.L. designed the animal experiments, conducted most of the experiments, analyzed data, and wrote the manuscript. H.L. and L.Y. were responsible for the collection and analysis of human samples. R.L., T.J., M.Y., S.W., Y.Y., C.C., H.L., and Y.D. assisted with exercise training, behavioral tests, tissue collection, tail vein injections, morphological experiments, and the isolation and characterization of small extracellular vesicles. X.G., W.W., and Y.D. conducted electrophysiological experiments. Y.C., S.W., J.Y., and Y.L. assisted with western blot analysis. H.L., L.Y., W.L., and L.C. supervised the project. C.R., L.Y., T.W., and J.T. reviewed and edited the manuscript. All authors approved the final manuscript for submission.

## Supporting information



Supporting Information

## Data Availability

The data that support the findings of this study are available from the corresponding author upon reasonable request.
