# Supplementary material for: Muscle‐Derived Small Extracellular Vesicles Mediate Exercise‐Induced Cognitive Protection in Chronic Cerebral Hypoperfusion
Source: Adv Sci (Weinh). 2025 Apr 24;12(27):2410209. doi: 10.1002/advs.202410209 (PMC12279231; doi:10.1002/advs.202410209)
Supplement: Supplementary file 1 — Supporting Information [file ADVS-12-2410209-s001.docx]

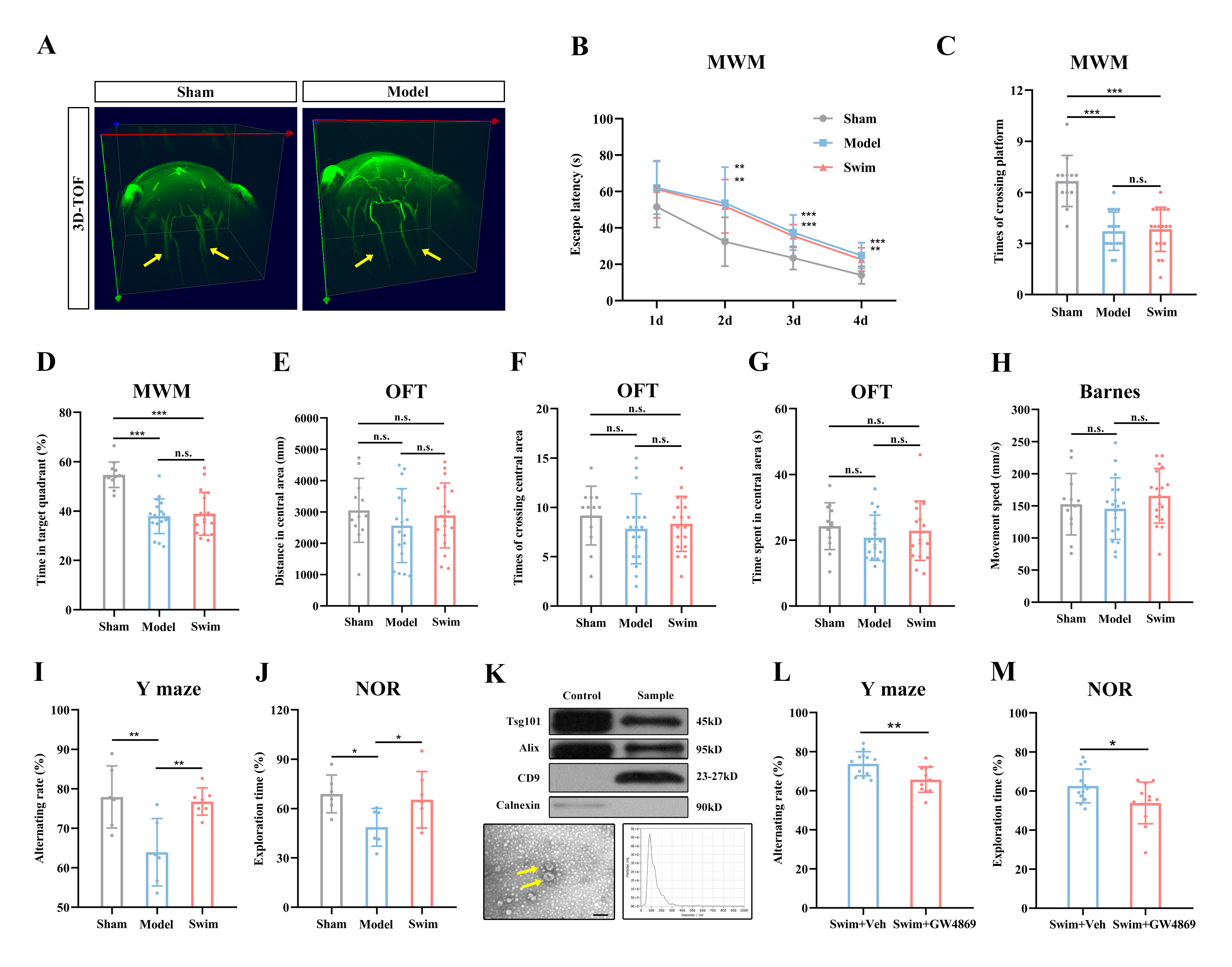


**Figure S1: Bilateral common carotid artery occlusion simulates vascular cognitive impairment induced by chronic cerebral hypoperfusion. (A)** Representative images of blood vessels detected by 3D-TOF after bilateral common carotid artery occlusion. **(B)** Latency to reach the escape platform in the learning stage of the MWM after the operation. **(C-D)** Number of crossings of the escape platform (C) and duration percentage in the target quadrant (D) during the probe trial after the operation. **(E-G)** Distance in the central area (E), number of crossings of the central area (F), and time spent in the central area (G) in the open field after swimming training. **(H)** Movement speed in the Barnes maze after swimming training. **(I)** Spontaneous alternation rate in the Y maze test after swimming training. **(J)** Discrimination index detected 1 hour after the learning stage in the NOR test after swimming training. **(K)** (Top) Representative western blots showing marker proteins of sEVs purified from rat serum. PC12 cell lysate was used as a positive control. Serum sEVs from any randomly selected rat were used as the sample; (Left bottom) Representative electron micrograph demonstrating sEVs isolation from the serum. Scale bar: 100 nm; (Right bottom) Representative results of nanoparticle tracking analysis displaying the size distribution of sEVs isolated from serum. **(L)** Spontaneous alternation rate in the Y maze test after GW4869 treatment. **(M)** Discrimination index detected 1 hour after the learning stage in the NOR test after GW4869 treatment. Data are presented as the mean ± SD. Sham group n=12, Model and Swim groups n=18 (B-H). Sham and Model groups n=6, Swim group n=7 (I and J). n=12 per group (K and L). ^*^*p* < 0.05, ^**^*p* < 0.01, ^***^*p* < 0.001. n.s., not significant. Statistical analysis was performed using two-way ANOVA for repeated measures (B), one-way ANOVA with Bonferroni post-hoc comparisons (C-J), and unpaired two-tailed Student’s *t*-test (K and L). 3D-TOF, Three-dimensional time-of-flight; ANOVA, Analysis of variance; MWM, Morris water maze; NOR, Novel object recognition; OFT, Open field test.


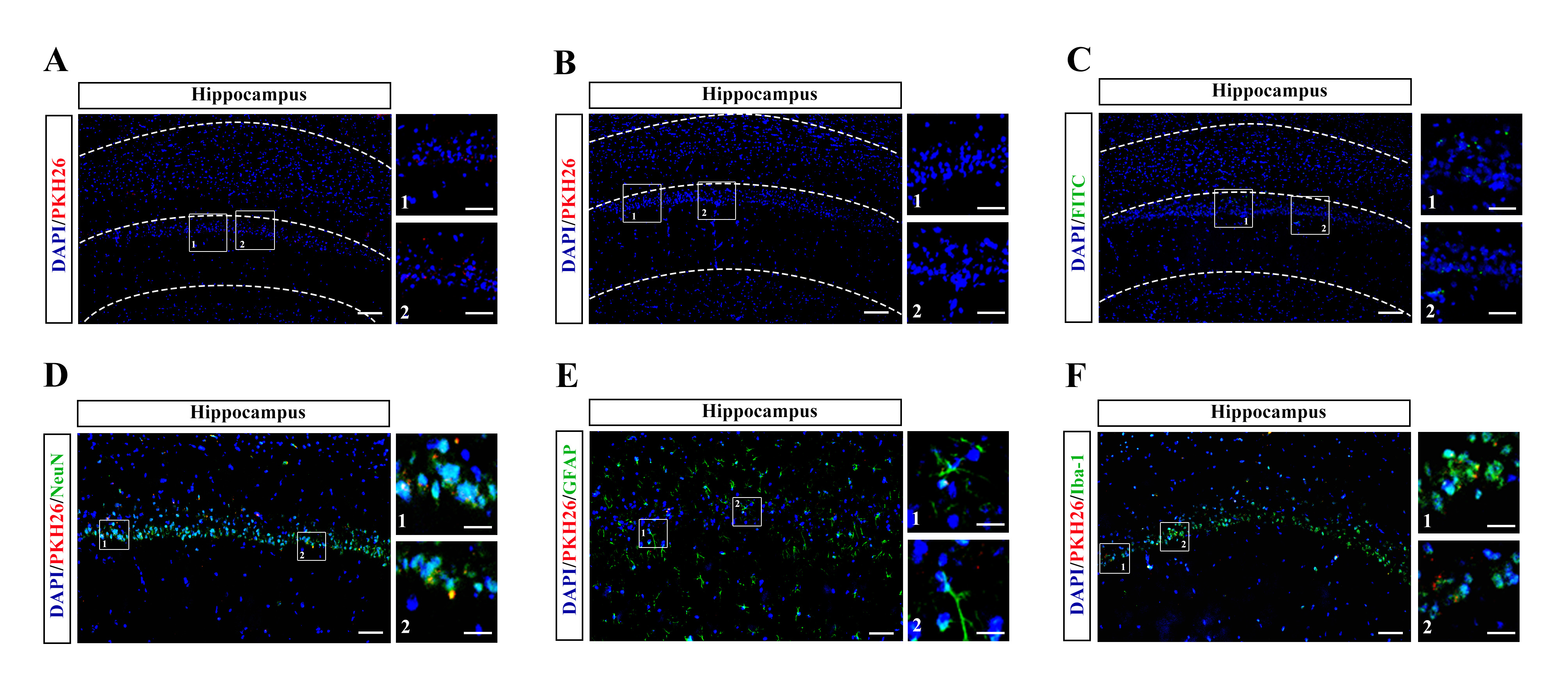


**Figure S2: sEVs can effectively cross the blood-brain barrier and were mostly localized in the neurons.** **(A-C)** Representative fluorescent image of sEVs in rat hippocampus injected with PKH26-labeled sEVs (A), PKH26-labeled PBS (B) and FITC-labeled sEVs (C). Scale bar: 100 μm in left views and 20 μm in right enlarged images. **(D-F)** Representative fluorescent image of the distribution of PKH26-labeled sEVs in neurons (D), astrocytes (E), and microglia (F) after intravenous injection. Scale bar: 50 μm in left views and 10 μm in right enlarged images.


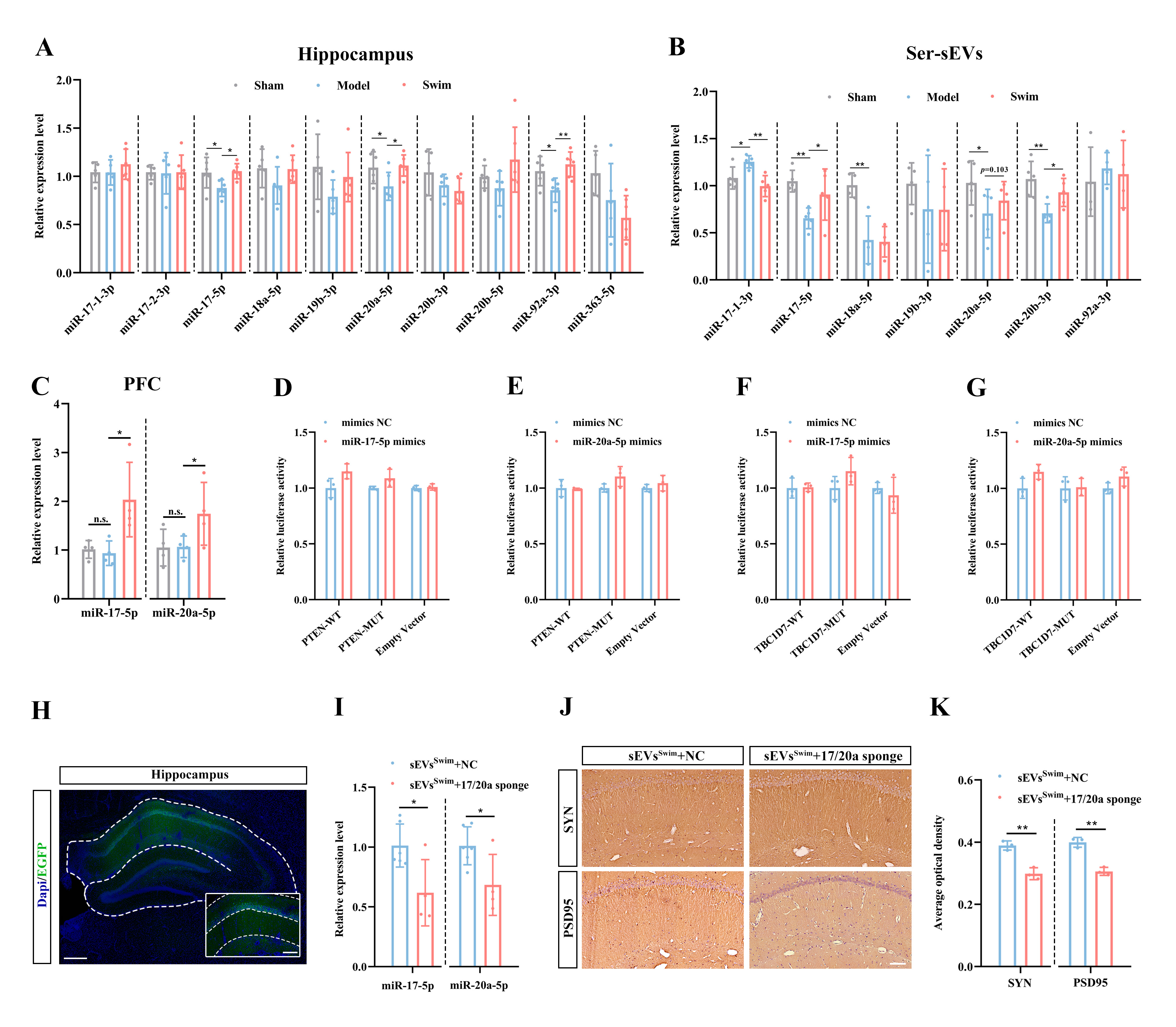


**Figure S3: Swimming-induced upregulation of synaptic proteins in CCH rats is mediated by miR-17/20a-5p.** **(A)** qPCR analysis of differentially expressed miRNAs in the hippocampus of Sham, Model, and Swim groups. **(B)** qPCR analysis of differentially expressed miRNAs in the serum sEVs of Sham, Model, and Swim groups. **(H)** Levels of miR-17/20a-5p in the prefrontal cortex of rats after swimming training. **(D-E)** Luciferase reporter assays for miR-17-5p (D), miR-20a-5p (E), and the PTEN 3'UTR with native or mutant binding sites. **(F-G)** Luciferase reporter assays for miR-17-5p (F), miR-20a-5p (G), and the TBC1D7 3'UTR with native or mutant binding sites. **(H)** Representative immunofluorescent images showing the site of viral transfection. Scale bar, 500 μm in panoramic views and 200 μm in enlarged images. **(I)** Levels of miR-17/20a-5p in the hippocampus after rAAV injection. **(J)** Representative immunohistochemical staining images showing expression levels of SYN and PSD95 in the hippocampus after rAAV injection. **(K)** Quantitative analysis of SYN and PSD95 in the hippocampus. Scale bar, 100 μm. Data are presented as the mean ± SD. n=6 per group (A and B). n=4 per group (C). n=3 per group (D-G and K). sEVs^Swim^+NC group n=6, sEVs^Swim^+17/20a sponge group n=4 (I). ^*^*p* < 0.05, ^**^*p* < 0.01. n.s., not significant. Statistical analysis was performed using one-way ANOVA with Bonferroni post-hoc comparisons (A-C), two-way ANOVA (D-G) and unpaired two-tailed Student’s *t*-test (I and K). 3'UTR, 3’Untranslated region; ANOVA, Analysis of variance; CCH, Chronic cerebral hypoperfusion; PFC, prefrontal cortex; PSD95, Postsynaptic density protein 95; PTEN, Phosphatase and tensin homolog deleted on chromosome ten; qPCR, Quantitative real-time polymerase chain reaction; rAAV, Recombination adeno-associated virus; sEVs, Small extracellular vesicles; SYN, Synapsin; TBC1D7, Tre2-Bub2-Cdc16-1 domain family member 7.


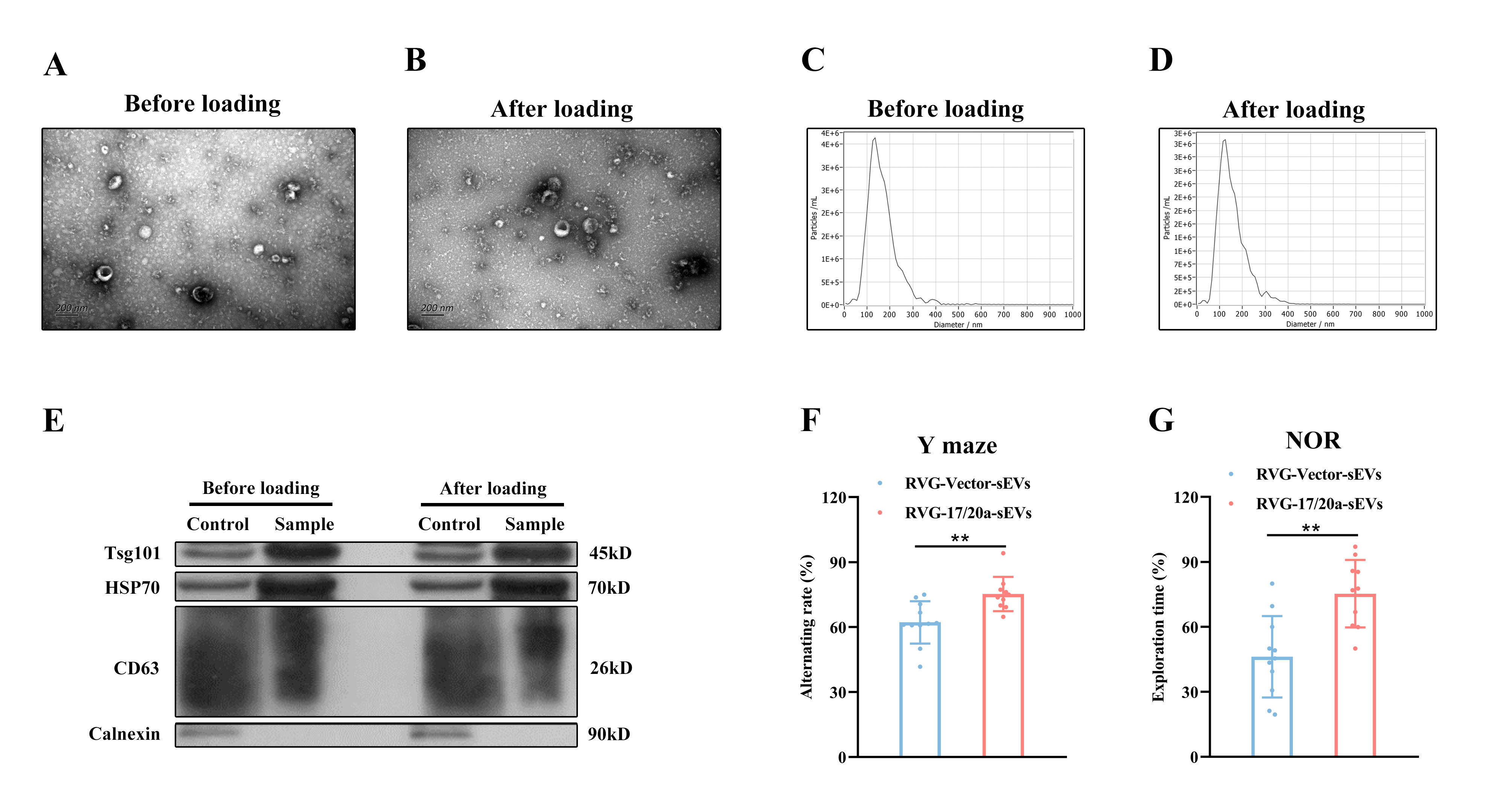


**Figure S4: Engineering transformation of EVs does not affect the morphology, size, and marker protein expression of sEVs. (A-B)** Representative electron micrographs of sEVs before (A) and after loading (B). Scale bar: 100 nm. **(C-D)** Representative results of nanoparticle tracking analysis demonstrating the size distribution of sEVs before (C) and after loading (D). **(E)** Representative western blots of EV marker proteins in sEVs before and after loading. **(F)** Spontaneous alternation rate in the Y maze test after administration of engineered RVG-sEVs. **(G)** Discrimination index detected 1 hour after the learning stage in the NOR test after administration of engineered RVG-sEVs. Data are presented as the mean ± SD. RVG-Vector-sEVs group n=11, RVG-17/20a-sEVs group n=10 (F and G). ^**^*p* < 0.01. Statistical analysis was performed using unpaired two-tailed Student’s *t*-test (F and G). NOR, Novel object recognition; RVG-sEVs, Rabies virus glycoprotein- Small extracellular vesicles; SD, Standard deviation; sEVs, Small extracellular vesicles.


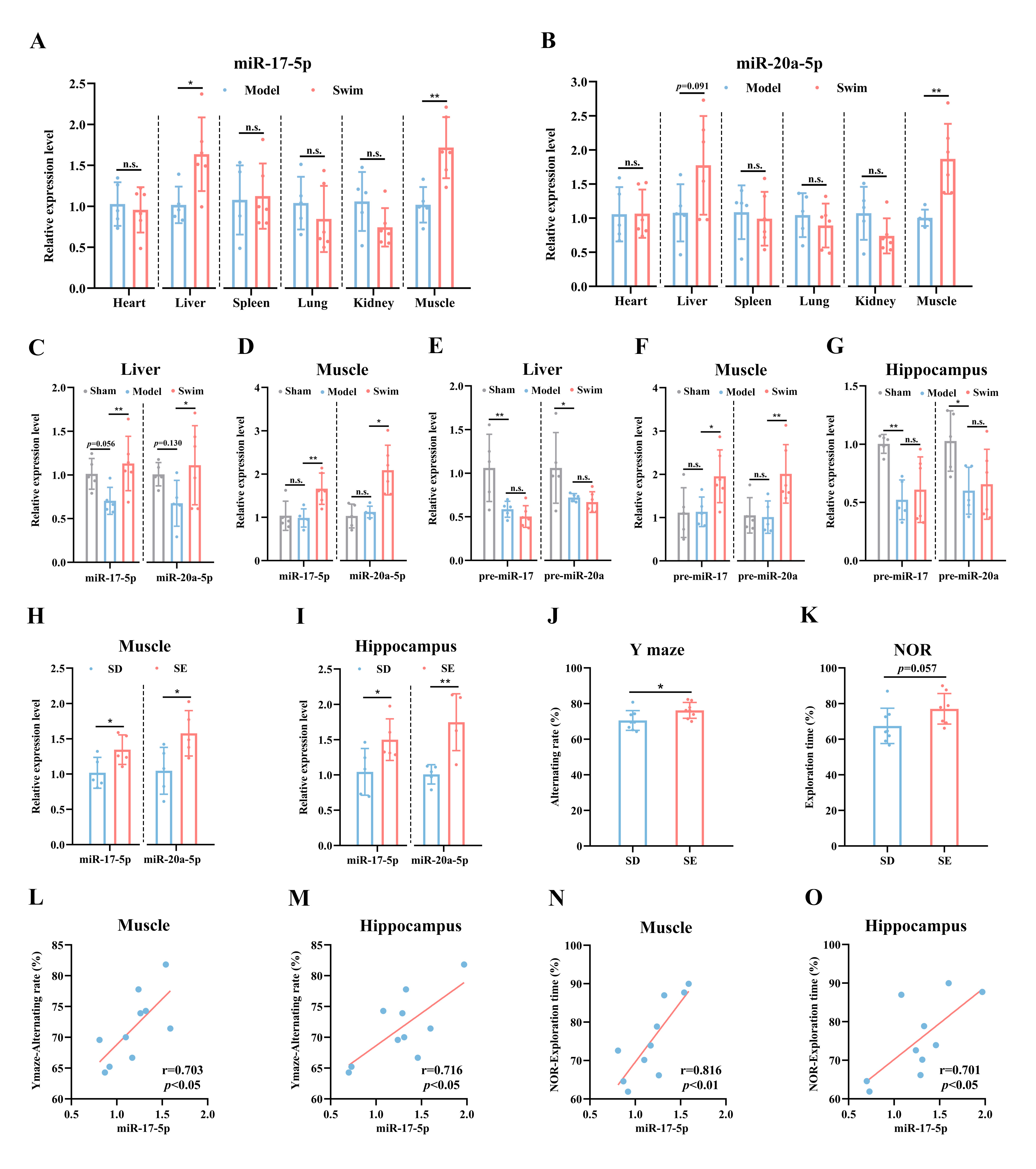


**Figure S5: Exercise-induced upregulation of miR-17/20a-5p in the muscle is an adaptive change independent of CCH. (A-B)** Expression profiles of miR-17-5p (A) and miR-20a-5p (B) in the heart, liver, spleen, lung, kidney, and muscle of CCH rats in response to swimming. **(C-D)** Levels of miR-17/20a-5p in the liver (C) and muscle (D) of CCH rats receiving swimming training. **(E-G)** Levels of pre-miR-17/20a-5p in the liver (E), muscle (F), and hippocampus (G) of CCH rats receiving swimming training. **(H-I)** Levels of miR-17/20a-5p in the muscle (H) and hippocampus (I) of SD rats receiving swimming training. **(J)** Spontaneous alternation rate in the Y maze test of SD rats receiving swimming training. **(K)** Discrimination index detected 1 hour after the learning stage in the NOR test of SD rats receiving swimming training. **(L-M)** Correlation analysis of the miR-17-5p concentration in the muscle (L) and hippocampus (M) with the alternation rate of the Y maze. **(N-O)** Correlation analysis of the miR-17-5p concentration in the muscle (N) and hippocampus (O) with the discrimination index of NOR. Data are presented as the mean ± SD. Sham and Model group n=5, Swim group n=6 (A-G). n=5 per group (H, I and L-O). n=10 per group (J and K). ^*^*p* < 0.05, ^**^*p* < 0.01, ^***^*p* < 0.001. n.s., not significant. Statistical analysis was performed using unpaired two-tailed Student’s *t*-test (A, B and H-K), one-way ANOVA with Bonferroni post-hoc comparisons (C-G) and Pearson’s correlation analysis (L-O). ANOVA, Analysis of variance; CCH, chronic cerebral ischemia; NOR, Novel object recognition; SD, Standard deviation.


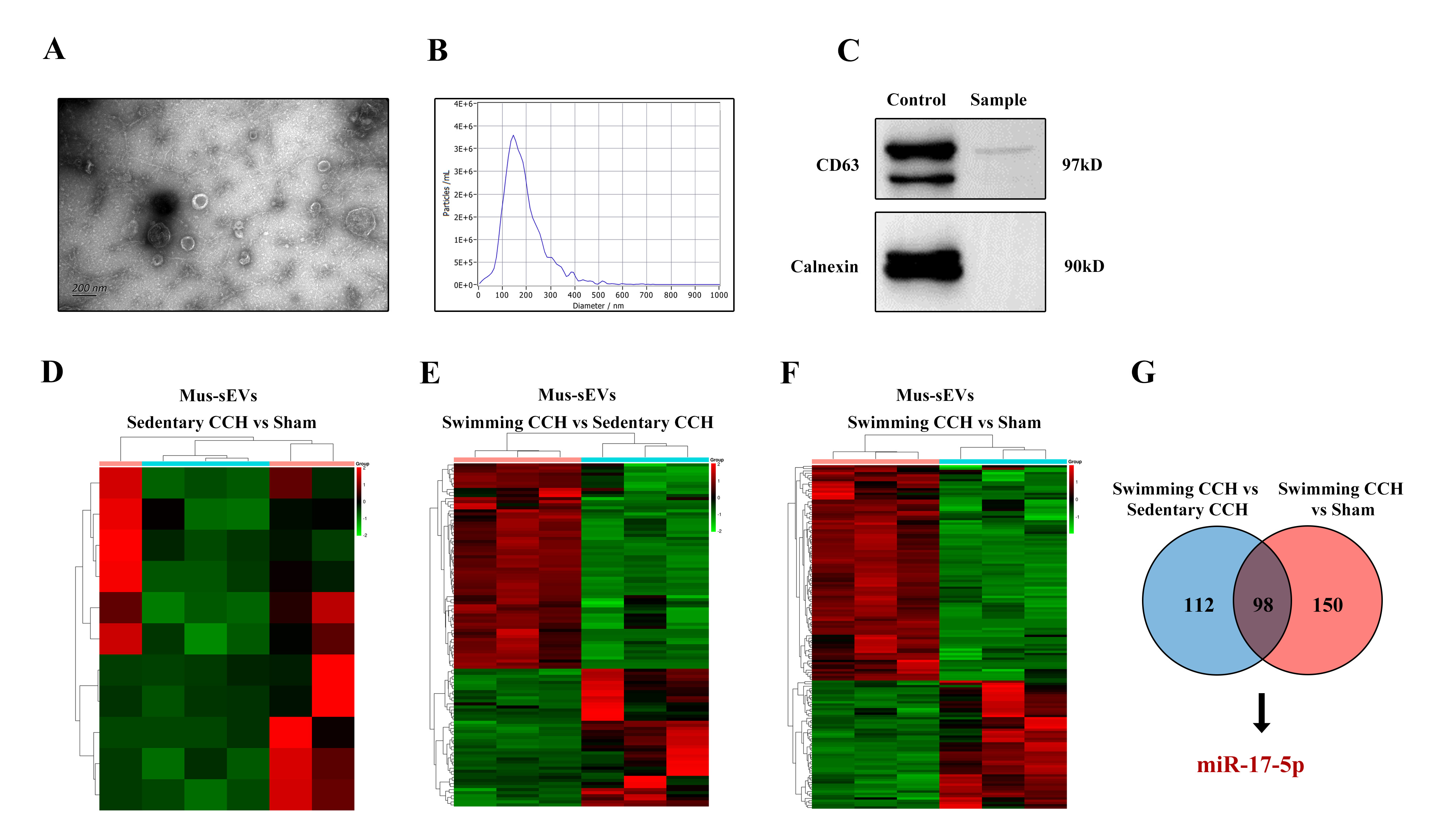


**Figure S6: Exercise markedly alters the miRNA expression profile in CCH rats compared with sham and sedentary CCH rats. (A)** Representative electron micrographs of sEVs isolated from muscle. Scale bar: 200 nm. **(B)** Representative results of nanoparticle tracking analysis demonstrating the size distribution of sEVs isolated from the muscle. **(C)** Representative western blots of EV marker proteins in sEVs isolated from the muscle. PC12 cell lysate was used as a positive control and the muscle sEVs from any randomly selected rat was used as the sample). **(D-F)** Heatmap of the differentially expressed miRNAs in muscle-derived sEVs between sedentary CCH and the sham group (D), between swimming CCH and sedentary CCH groups (E), and between swimming CCH and sham groups (F), respectively. No significant difference was observed between sham and sedentary rats. **(G)** Venn diagram showing the unique and overlapping differentially expressed miRNAs in muscle-derived sEVs between the swimming CCH and sedentary CCH groups, and between the swimming CCH and sham groups. Data are presented as the mean ± SD. n=3 per group (D-F). CCH, Chronic cerebral ischemia; Mus-sEVs, Small extracellular vesicle isolated from muscle; SD, Standard deviation.


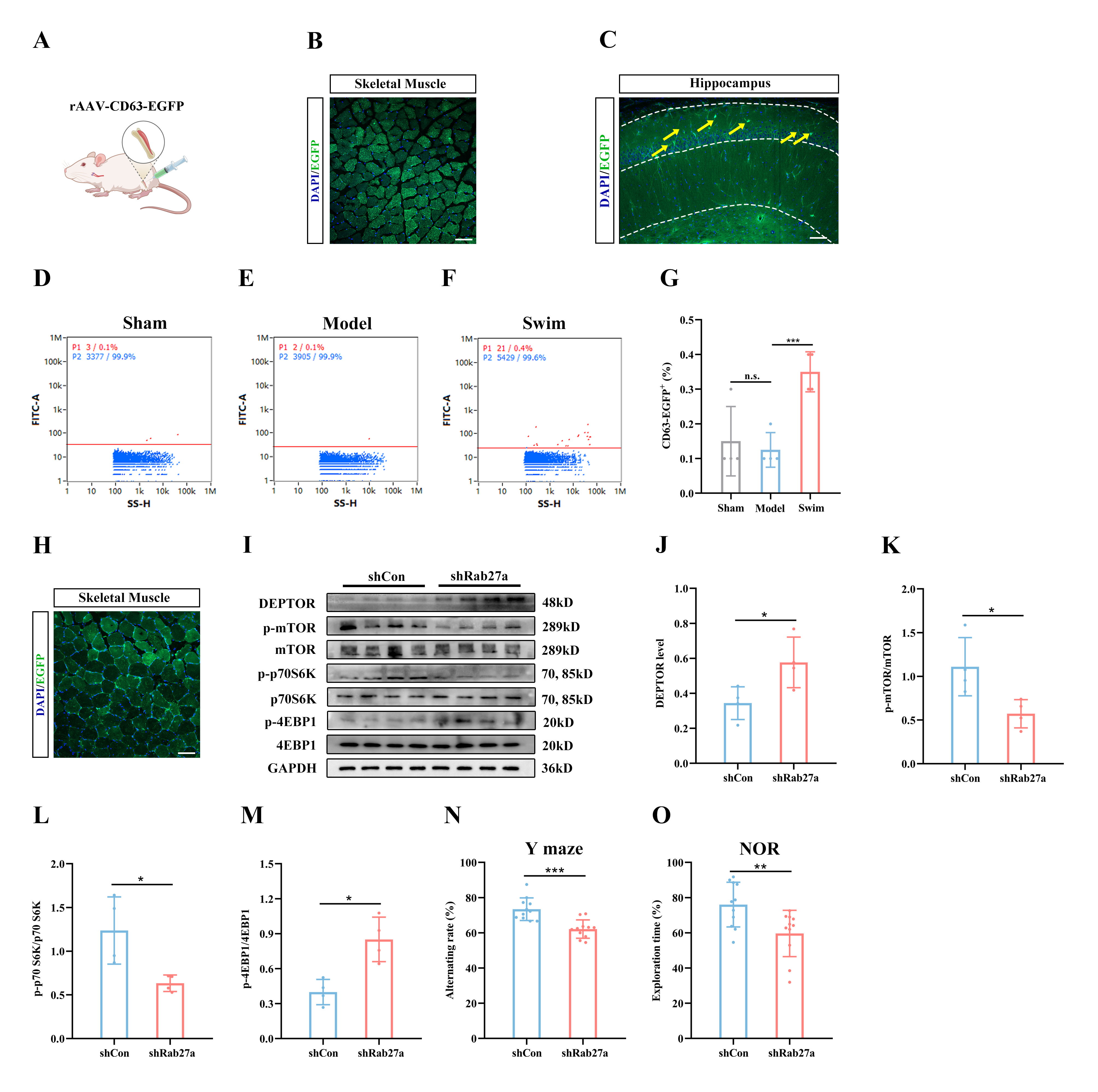


**Figure S7:** **Muscle-derived sEVs can enter the brain *in vivo* and mediate the exercise-induced activation of mTOR pathway.** **(A)** Schematic diagram illustrating rats receiving *in situ* intramuscular injection of rAAV-CD63-EGFP. **(B)** Representative immunofluorescent images showing the site of viral transfection in muscle injected with rAAV-CD63-EGFP. Scale bar: 100 μm. **(C)** Representative fluorescent image of sEVs in the hippocampus of rats injected with rAAV-CD63-EGFP. Scale bar: 100 μm. **(D-F)** Representative results of EGFP^+^ sEVs in serum sEVs of sham (D), model (E), and swim (F) groups. **(G)** Quantitative analysis of EGFP^+^ sEVs contents in serum sEVs. **(H)** Representative immunofluorescent images showing the site of viral transfection in muscle injected with rAAV-Rab27a-shRNA. Scale bar: 100 μm. **(I)** Representative western blots showing the DEPTOR and the downstream mTOR pathway in the hippocampus after rAAV injection in the muscle. **(J-M)** Quantitative analysis of DEPTOR (J), p-mTOR/mTOR (K), p-p70S6K/p70S6K (L), and p-4EBP1/4EBP1 (M) in the hippocampus. **(N)** Spontaneous alternation rate in the Y maze test after rAAV injection in the muscle. **(O)** Discrimination index detected 1 hour after the learning stage in the NOR test after rAAV injection in the muscle. Data are presented as the mean ± SD. n=4 per group (G and J-M). n=11 per group (N and O). ^*^*p* < 0.05, ^**^*p* < 0.01, ^***^*p* < 0.001. n.s., not significant. Statistical analysis was performed using one-way ANOVA with Bonferroni post-hoc comparisons (G) and unpaired two-tailed Student’s *t*-test (J-O). ANOVA, Analysis of variance; fEPSP, Field excitatory postsynaptic potential; HFS, High frequency stimulation; mTOR, Mechanistic target of rapamycin; NOR, Novel object recognition; rAAV, Recombination adeno-associated virus; SD, Standard deviation; sEVs, Small extracellular vesicles.


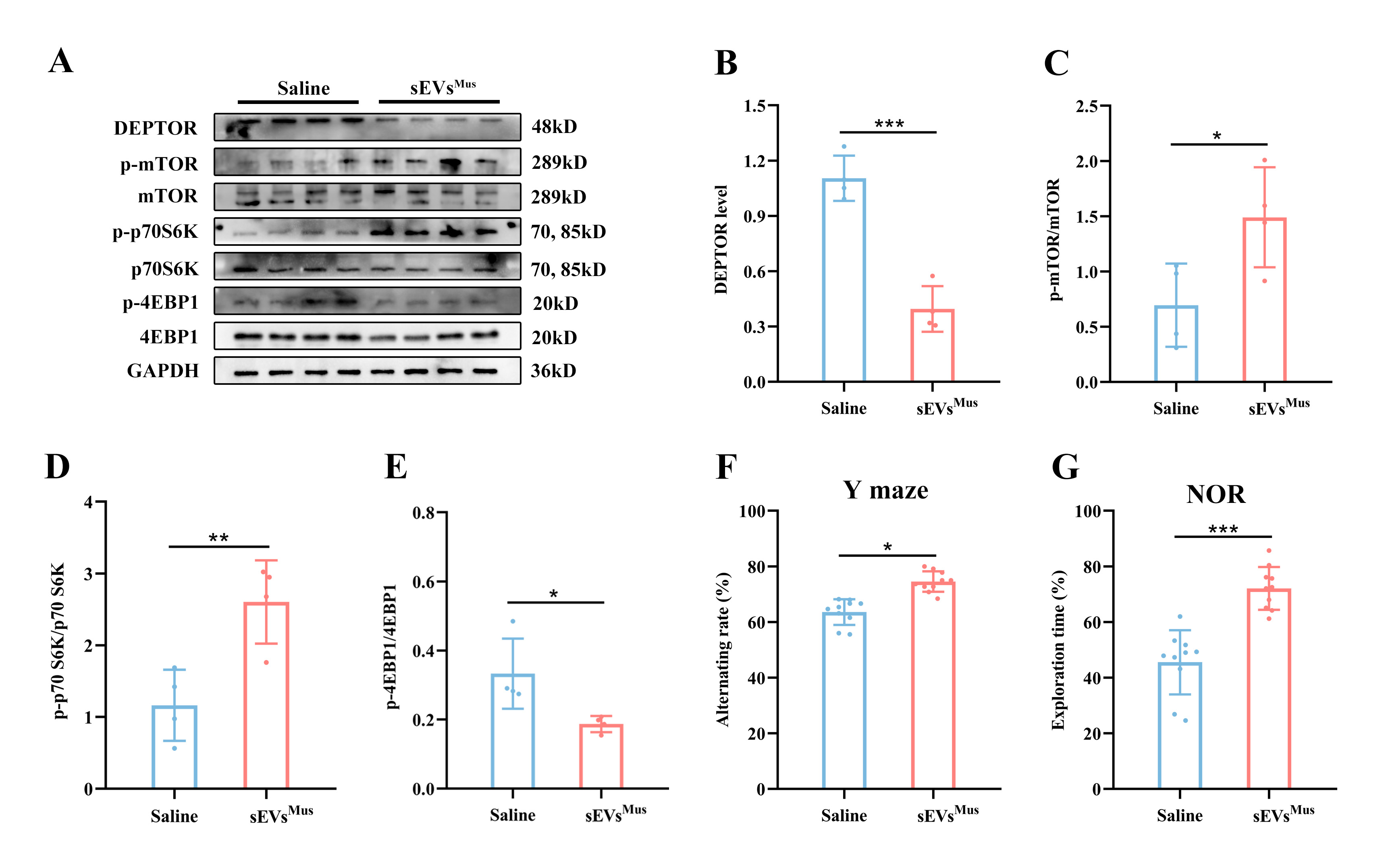


**Figure S8: Treatment of swimming-derived muscle sEVs activates mTOR pathway in CCH rats.** **(A)** Representative western blots showing the DEPTOR and the downstream mTOR pathway in the hippocampus of rats treated with muscle-derived sEVs. **(B-E)** Quantitative analysis of DEPTOR (B), p-mTOR/mTOR (C), p-p70S6K/p70S6K (D), and p-4EBP1/4EBP1 (E) in the hippocampus. **(F)** Spontaneous alternation rate in the Y maze test after delivery of muscle-derived sEVs. **(G)** Discrimination index detected 1 hour after the learning stage in the NOR test after delivery of muscle-derived sEVs. Data are presented as the mean ± SD. n=4 per group (B-E). n=10 per group (F and G). ^*^*p* < 0.05, ^**^*p* < 0.01, ^***^*p* < 0.001. Statistical analysis was performed using unpaired two-tailed Student’s *t*-test (B-G). CCH, Chronica cerebral ischemia; NOR, Novel object recognition; sEVs, Small extracellular vesicles.


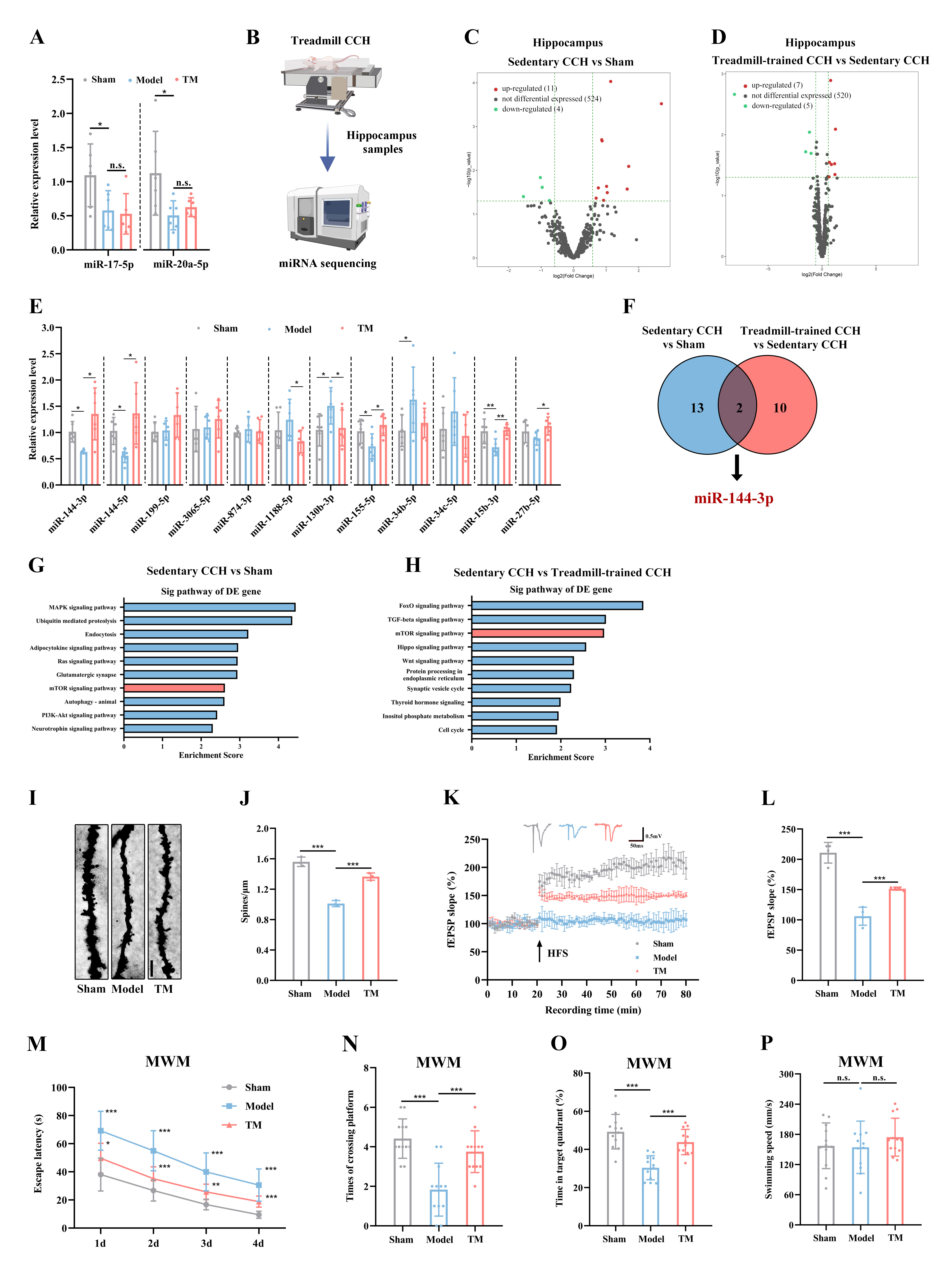


**Figure S9: Treadmill training modulates expression profiles of miRNAs and enhances synaptic plasticity in CCH rats. (A)** Levels of miR-17/20a-5p in the hippocampus of rats after treadmill training. **(B)** Schematic diagram illustrating miRNA sequencing in the hippocampus after treadmill training. **(C-D)** Volcano map presentation of relative miRNA differences between the sedentary CCH and sham groups (C), and between the treadmill-trained CCH and sedentary CCH groups (D) in the hippocampus. **(E)** qPCR analysis of differentially expressed miRNAs in the hippocampus of three groups. **(F)** Venn diagram showing unique and overlap differentially expressed miRNAs between the sedentary CCH and sham groups, and between the treadmill-trained CCH and sedentary CCH groups. **(G)** Signaling pathway enrichment analysis of differentially expressed miRNAs in the hippocampus between the sedentary CCH and sham groups. **(H)** Signaling pathway enrichment analysis of differentially expressed miRNAs in the hippocampus between the treadmill-trained CCH and sedentary CCH groups. **(I)** Representative images of Golgi staining after treadmill training. Scale bar: 2 μm. **(J)** Quantitative analysis of dendritic spine density determined by Golgi staining. **(K)** Time-plot of normalized fEPSP slopes recorded from hippocampal slices after treadmill training. Representative traces of fEPSPs in the Sham, Model, and TM groups. **(L)** Histogram representing the average normalized fEPSP slopes during the last 10 minutes of recording following HFS, represented as a percentage of the baselines for each group. **(M)** Latency to reach the escape platform in the learning stage of the MWM after treadmill training. **(N-O)** Number of crossings of the escape platform (N) and duration percentage in the target quadrant (O) during the probe trial after treadmill training. **(P)** Swimming speed in the MWM after treadmill training. Data are presented as the mean ± SD. n=6 per group (A and E). n=3 per group (C, D and J). n=4 per group (K and L). n=12 per group (M-P). ^*^*p* < 0.05, ^**^*p* < 0.01, ^***^*p* < 0.001. n.s., not significant. Statistical analysis was performed using one-way ANOVA with Bonferroni post-hoc comparisons (A, E, J, L, and N-P) and two-way ANOVA for repeated measures (M). 3'UTR, 3’Untranslated region; ANOVA, Analysis of variance; CCH, Chronic cerebral hypoperfusion; fEPSP, Field excitatory postsynaptic potential; HFS, High frequency stimulation; MWM, Morris water maze


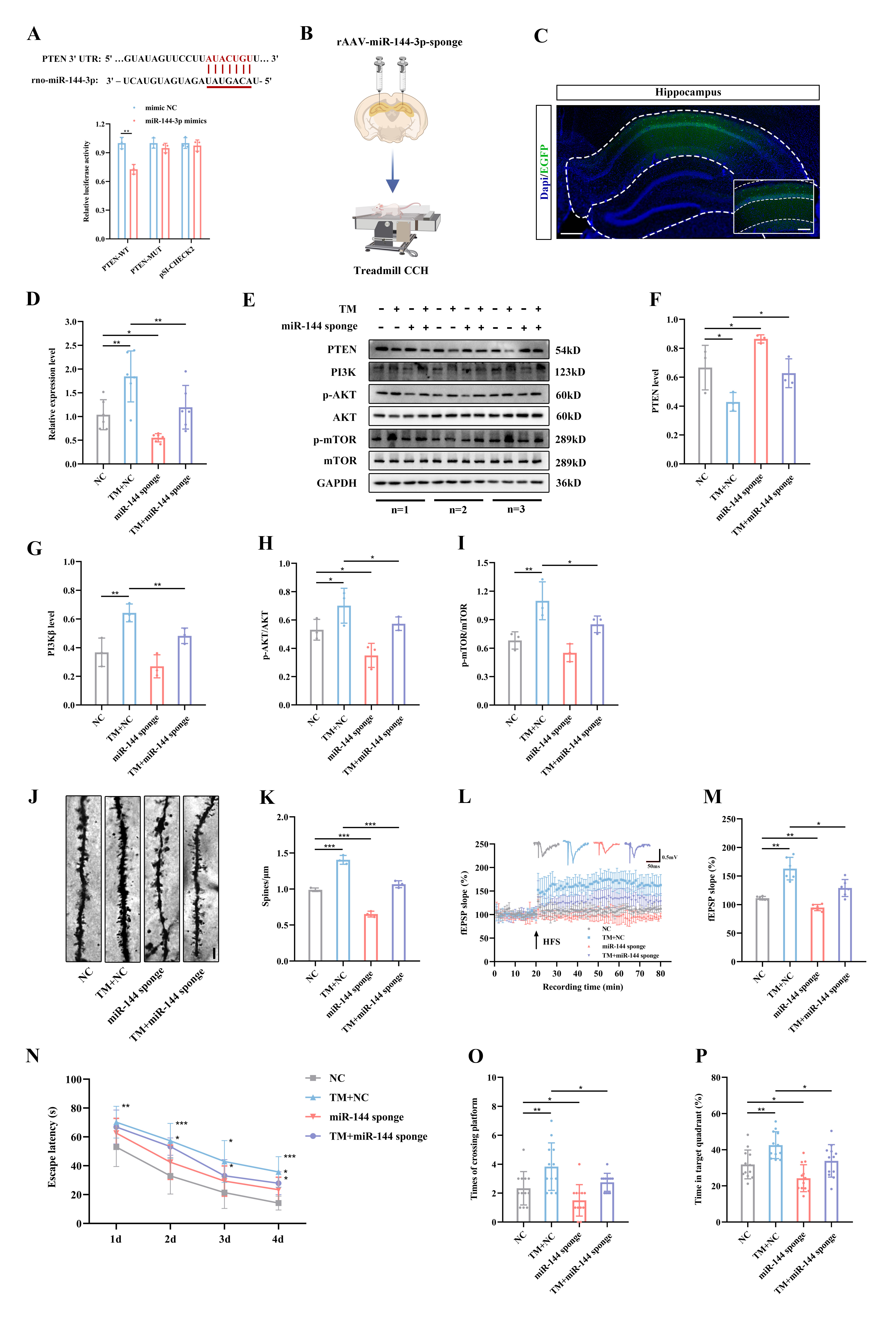


**Figure S10: miR-144-3p is crucial for cognitive protection induced by treadmill training in CCH rats. (A)** Predicted binding sequences of miR-144-3p in the 3'UTR of PTEN. Luciferase reporter assays for miR-144-3p and the PTEN 3'UTR with native or mutant binding sites. **(B)** Schematic diagram depicting rats receiving rAAV injection in the hippocampus followed by treadmill training. **(C)** Representative immunofluorescent images showing the site of viral transfection. Scale bar: 500 μm in panoramic views and 200 μm in enlarged images. **(D)** Levels of miR-144-3p in the hippocampus after rAAV injection. **(E)** Representative western blots showing putative target genes and the downstream PI3K/AKT/mTOR pathway in the hippocampus treated with rAAV-miR-144-3p-sponge. **(F-I)** Quantitative analysis of PTEN (F), PI3K (G), p-AKT/AKT (H), and p-mTOR/mTOR (I) in the hippocampus. **(J)** Representative images of Golgi staining after rAAV infection in the hippocampus. Scale bar: 2 μm. **(K)** Quantitative analysis of dendritic spine density determined by Golgi staining. **(L)** Time-plot of normalized fEPSP slopes recorded from hippocampal slices after rAAV infection in the hippocampus. Representative traces of fEPSPs in the NC, TM+NC, miR-144 sponge, and TM+miR-144-sponge groups. **(M)** Histogram representing the average normalized fEPSP slopes during the last 10 minutes of recording following HFS, represented as a percentage of the baselines for each group. **(N)** Latency to reach the escape platform in the learning stage of the MWM after rAAV infection in the hippocampus. **(O-P)** Number of crossings of the escape platform (O) and duration percentage in the target quadrant (P) during the probe trial after rAAV infection in the hippocampus. Data are presented as the mean ± SD. n=3 per group (A, E-I, and K). n=6 per group (D, L and M). n=12 per group (N-P). ^*^*p* < 0.05, ^**^*p* < 0.01, ^***^*p* < 0.001. Statistical analysis was performed using two-way ANOVA (A), one-way ANOVA with Bonferroni post-hoc comparisons (D, F-I, K, M, O, and P), and two-way ANOVA for repeated measures (N). 3'UTR, 3’Untranslated region; ANOVA, Analysis of variance; AKT, protein kinase B; CCH, Chronic cerebral hypoperfusion; fEPSP, Field excitatory postsynaptic potential; HFS, High frequency stimulation; MWM, Morris water maze; mTOR, Mechanistic target of rapamycin; PI3K, phosphatidylinositol-3 kinase; PTEN, Phosphatase and tensin homolog deleted on chromosome ten; rAAV, Recombination adeno-associated virus.


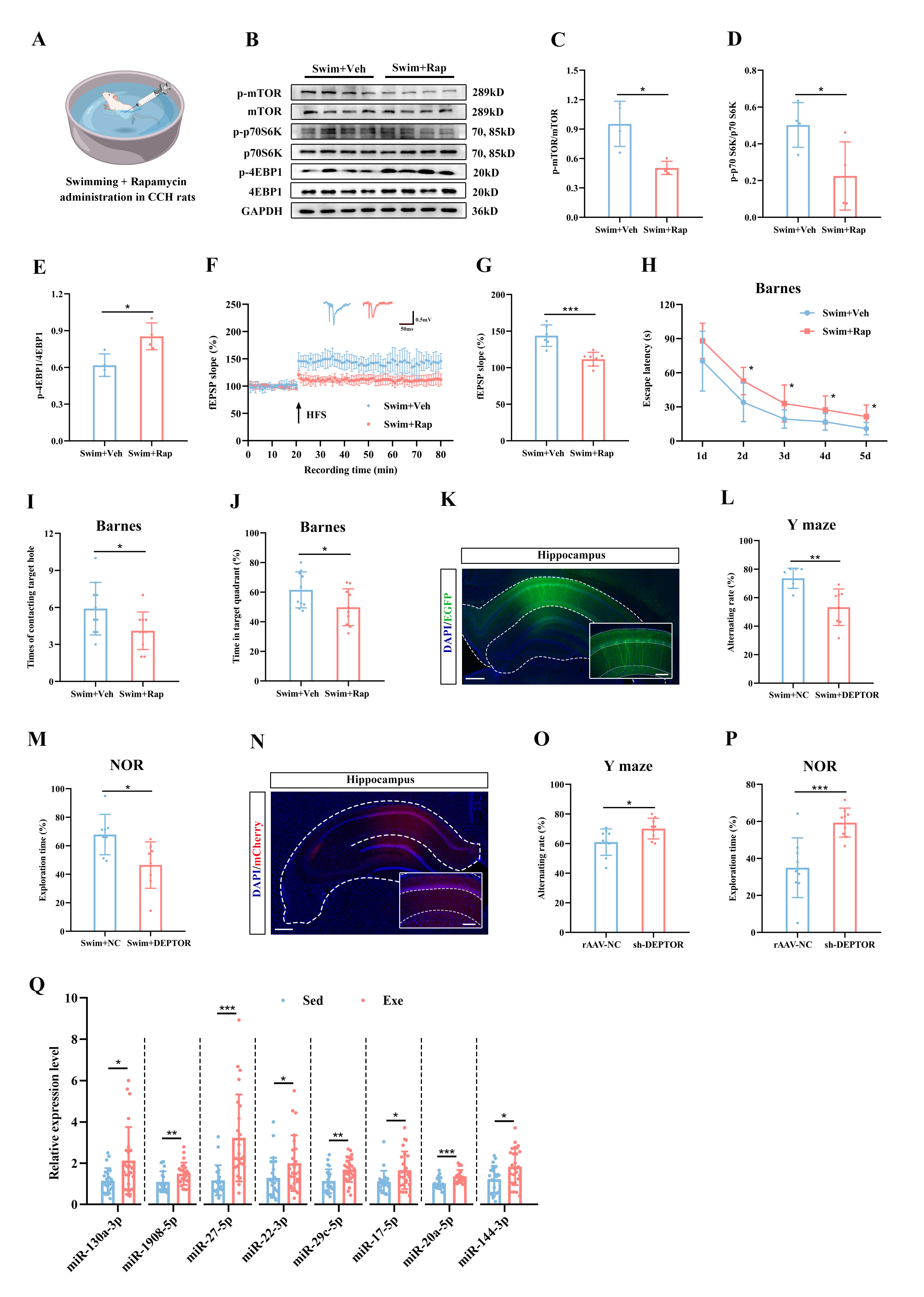


**Figure S11: mTOR activation contributes to exercise-induced cognitive improvement. (A)** Schematic diagram illustrating rats receiving swimming training and rapamycin administration (2.0 mg/kg, 5 times each week for 4 weeks). **(B)** Representative western blots of mTOR and the downstream p70S6K/4EBP1 in the hippocampus after rapamycin treatment. **(C-E)** Quantitative analysis of p-mTOR/mTOR (C), p-p70S6K/p70S6K (D), and p-4EBP1/4EBP1 (E) in the hippocampus. **(F)** Time-plot of normalized fEPSP slopes recorded from hippocampal slices after rapamycin treatment. Representative traces of fEPSPs in the Swim+Veh and Swim+Rap groups. **(G)** Histogram representing the average normalized fEPSPs slope during the last 10 minutes of recording following HFS, represented as a percentage of the baselines for each group. **(H)** Latency to reach the target hole in the learning stage of the Barnes maze after rapamycin treatment. **(I-J)** Number of contacts with the target hole (I) and duration percentage in the target quadrant (J) during the probe trial after rapamycin treatment. **(K)** Representative immunofluorescent images showing the site of viral transfection of rAAV-DEPTOR-overexpression. Scale bar: 500 μm in panoramic views and 200 μm in enlarged images. **(L)** Spontaneous alternation rate in the Y maze test after rAAV injection in the hippocampus. **(M)** Discrimination index detected 1 hour after the learning stage in the NOR test after rAAV injection in the hippocampus. **(N)** Representative immunofluorescent images showing the site of viral transfection of rAAV-DEPTOR-shRNA. Scale bar: 500 μm in panoramic views and 200 μm in enlarged images. **(O)** Spontaneous alternation rate in the Y maze test after rAAV injection in the hippocampus. **(P)** Discrimination index detected 1 hour after the learning stage in the NOR test after rAAV injection in the hippocampus. **(Q)** qPCR analysis of differentially expressed miRNAs in sEVs of sedentary and long-term exercise individuals. Data are presented as the mean ± SD. n=4 per group (B-E). Swim+Veh group n=6, Swim+Rap group n=7 (F and G). n=10 per group (H-J). n=8 per group (L and M). n=9 per group (O and P). Sed group n=22, Exe group n=24 (D). ^*^*p* < 0.05, ^**^*p* < 0.01, ^***^*p* < 0.001. Statistical analysis was performed using unpaired two-tailed Student’s *t*-test (C-E, G, I, J, L, M, and O-Q) and two-way ANOVA for repeated measures (H). mTOR, Mechanistic target of rapamycin; NOR, Novel object recognition; qPCR, Quantitative real-time polymerase chain reaction; rAAV, Recombination adeno-associated virus; sEVs, Small extracellular vesicles.

**Table S1. Antibodies used in this study.**

| **REAGENT or RESOURCE** | **SOURCE** | **IDENTIFIER** |
| --- | --- | --- |
| **Anti-Synaptophysin antibody [YE269]** | Abcam | Cat# ab32127, RRID: AB_2286949 |
| **PSD95 (D74D3) XP** | Cell Signaling Technology | Cat# 3409, RRID: AB_1264242 |
| **Anti-Neurogranin** | Sigma-Aldrich | Cat# AB5620, RRID: AB_91937 |
| **Anti-phospho-Neurogranin (Ser36)/Neuromodulin (Ser41) Antibody** | Sigma-Aldrich | Cat# 07-430, RRID: AB_310609 |
| **Anti-Calmodulin 1/2/3 antibody [EP799Y] - C-terminal** | Abcam | Cat# ab45689, RRID: AB_725815 |
| **Anti-CaMKII antibody [EP1829Y]** | Abcam | Cat# ab52476, RRID: AB_868641 |
| **Phospho-CaMKII (Thr286) (D21E4)** | Cell Signaling Technology | Cat# 12716, RRID: AB_2713889 |
| **Anti-PTEN antibody [Y184]** | Abcam | Cat# ab32199, RRID: AB_777535 |
| **PI3 Kinase p110α Antibody** | Cell Signaling Technology | Cat# 611398, RRID: AB_398920 |
| **Anti-PI3 Kinase p110 beta antibody [EPR5515(2)]** | Abcam | Cat# 151549, RRID: AB_151549 |
| **Akt (pan) (C67E7) Rabbit mAb** | Cell Signaling Technology | Cat# 4691, RRID: AB_915783 |
| **Phospho-Akt (Ser473) (D9E) XP® Rabbit mAb** | Cell Signaling Technology | Cat# 4060, RRID: AB_2315049 |
| **DEPTOR/DEPDC6 (D9F5)** | Cell Signaling Technology | Cat# 11816, RRID: AB_2750575 |
| **mTOR (7C10) Rabbit mAb** | Cell Signaling Technology | Cat# 2983, RRID: AB_2105622 |
| **Phospho-mTOR (Ser2448) Antibody** | Cell Signaling Technology | Cat# 2971, RRID: AB_330970 |
| **p70 S6 Kinase** | Cell Signaling Technology | Cat# 9202, RRID: AB_331676 |
| **Phospho-p70 S6 Kinase (Thr389) Antibody** | Cell Signaling Technology | Cat# 9205, RRID: AB_330944 |
| **Phospho-p70 S6 Kinase (Thr389) (108D2)** | Cell Signaling Technology | Cat# 9234, RRID: AB_2269803 |
| **4E-BP1 (53H11)** | Cell Signaling Technology | Cat# 9644, RRID: AB_2097841 |
| **Phospho-4E-BP1 (Thr37/46) (236B4)** | Cell Signaling Technology | Cat# 2855, RRID: AB_560835 |
| **Anti-TSG101 [EPR7130(B)]** | Abcam | Cat# ab125011, RRID: AB_10974262 |
| **Anti-Hsp70 [EPR16892]** | Abcam | Cat# ab181606, RRID: AB_2910093 |
| **Anti-CD63 [EPR5702]** | Abcam | Cat# ab134045, RRID: AB_2800495 |
| **Calnexin Polyclonal antibody** | Proteintech | Cat#10427-2-AP, RRID: AB_2069033 |
| **Anti-ALIX [EPR15314]** | Abcam | Cat# ab186429, RRID: AB_2754981 |
| **CD81 Polyclonal antibody** | Proteintech | Cat# 27855-1-AP, RRID: AB_2880995 |
| **CD9 Monoclonal antibody** | Proteintech | Cat# 60232-1-Ig, RRID: AB_11232215 |
| **GAPDH Monoclonal antibody** | Proteintech | Cat# 60004-1-Ig, RRID: AB_2107436 |
| **HRP-conjugated Affinipure Goat Anti-Rabbit IgG(H+L)** | Proteintech | Cat#SA00001-2, RRID: AB_2722564 |
| **HRP-conjugated Affinipure Goat Anti-Mouse IgG(H+L)** | Proteintech | Cat#SA00001-1, RRID: AB_2722565 |
| **Anti-NeuN antibody** **[EPR12763]** | Abcam | Cat# ab177487, RRID: AB_2532109 |
| **Anti-GFAP antibody** **[EPR1034Y]** | Abcam | Cat# ab68428, RRID: AB_1209224 |
| **Anti-Iba1 antibody** **[EPR16589]** | Abcam | Cat# ab178847, RRID: AB_2832244 |
| **Goat Anti-Rabbit IgG H&L (Alexa Fluor® 488)** | Abcam | Cat# ab150077, RRID: AB_2630356 |

**Table S2. Bacterial and virus strains used in this study.**

| **REAGENT or RESOURCE** | **SOURCE** | **IDENTIFIER** |
| --- | --- | --- |
| **rAAV2/9-CMV-EGFP-WPRE-miR-17-5p (sponge)-miR-20a-5p(sponge)-hGH polyA** | BrianVTA | PT-3078 |
| **rAAV2/9-CMV-EGFP-WPRE-pre-rno-miR-17-1-hGH polyA** | BrianVTA | PT-6536 |
| **rAAV2/9-CMV-mCherry-WPRE-pre-rno-miR-20a-hGH polyA** | BrianVTA | PT-6537 |
| **rAAV2/9-CMV-EGFP-miR-144-3p (sponge)-hGH polyA** | BrianVTA | PT-3967 |
| **rAAV2/9-CMV-mCherry-WPRE-hGH polyA** | BrianVTA | PT-1223 |
| **rAAV2/9-CMV-EGFP-WPRE-hGH polyA** | BrianVTA | PT-1316 |
| **rAAV2/9-hSyn-mCherry-5'miR-30a-shRNA3(Deptor)-3'-miR30a-WPREs** | BrianVTA | PT-6694 |
| **rAAV2/9-hSyn-mCherry-5'miR-30a-shRNA(scramble)-3'miR-30a-WPREs** | BrianVTA | PT-2321 |
| **MyoAAV2A-MHCK7-CD63-EGFP-WPREs** | BrianVTA | PT-9991 |
| **rAAV2/9-MHCK7-EGFP-5'miR-30a-shRNA(Rab27a)-3'miR-30a** | Brain Case | BC-2624 |
| **rAAV2/9-MHCK7-EGFP-5'miR-30a-shRNA(scrambie)-3'miR-30a** | Brain Case | BC-2625 |
| **pAAV2/9-CMV-Deptor-3xFLAG-EF1a-EGFP-tWPA** | Obio Technology | H29324 |
| **pAAV2/9-CMV-MCS-EFla-EGFP-tWPA** | Obio Technology | GL3012 |

**Table S3. Sequence of primers used in this study.**

| **GENE SEQUENCE** | **SOURCE** | **IDENTIFIER** |
| --- | --- | --- |
| **rno-miR-17-1-3p primers: Forward: ACTGCAGTGAAGGCACTTGTGG; Reverse: AGTGCAGGGTCCGAGGTATT** | Integrated DNA Technologies | N/A |
| **rno-miR-17-2-3p primers: Forward: CGACTGCACTGCAAGCACTTCTTAC; Reverse: AGTGCAGGGTCCGAGGTATT** | Integrated DNA Technologies | N/A |
| **rno-miR-17-5p primers: Forward: GCCGCAAAGTGCTTACAGTGC; Reverse: AGTGCAGGGTCCGAGGTATT** | Integrated DNA Technologies | N/A |
| **rno-miR-18a-5p primers: Forward: CCGCTAAGGTGCATCTAGTGCAGATAG; Reverse: AGTGCAGGGTCCGAGGTATT** | Integrated DNA Technologies | N/A |
| **rno-miR-19b-3p primers: Forward: CCGTGTGCAAATCCATGCAAAACTGA; Reverse: AGTGCAGGGTCCGAGGTATT** | Integrated DNA Technologies | N/A |
| **rno-miR-20a-5p primers: Forward: CCGCGCGTAAAGTGCTTATAGTGC; Reverse: AGTGCAGGGTCCGAGGTATT** | Integrated DNA Technologies | N/A |
| **rno-miR-20b-3p primers: Forward: CACTGCAGTGTGAGCACTTCTGG; Reverse: AGTGCAGGGTCCGAGGTATT** | Integrated DNA Technologies | N/A |
| **rno-miR-20b-5p primers: Forward: CGCAAAGTGCTCATAGTGCAGGTAG; Reverse: AGTGCAGGGTCCGAGGTATT** | Integrated DNA Technologies | N/A |
| **rno-miR-363-5p primers: Forward: CCGGGTGGATCACGATGCAATTT; Reverse: AGTGCAGGGTCCGAGGTATT** | Integrated DNA Technologies | N/A |
| **rno-miR-92a-3p primers: Forward: TATTGCACTTGTCCCGGCCTG; Reverse: AGTGCAGGGTCCGAGGTATT** | Integrated DNA Technologies | N/A |
| **rno-U6 primers: Forward: CTCGCTTCGGCAGCACATATACT; Reverse: ACGCTTCACGAATTTGCGTGTC** | Integrated DNA Technologies | N/A |
| **rno-miR-144-3ps: Forward: GCCGCGCCGTACAGTATAGATGA; Reverse: AGTGCAGGGTCCGAGGTATT** | Integrated DNA Technologies | N/A |
| **rno-miR-144-5p primers: Forward: CGCGCGGGATATCATCATATACT; Reverse: AGTGCAGGGTCCGAGGTATT** | Integrated DNA Technologies | N/A |
| **rno-miR-199-5p primers: Forward: GCCCAGTGTTCAGACTACCTGTTC; Reverse: AGTGCAGGGTCCGAGGTATT** | Integrated DNA Technologies | N/A |
| **rno-miR-3065-5p primers: Forward: CCGCGTCAACAAAATCACTGATGCT; Reverse: AGTGCAGGGTCCGAGGTATT** | Integrated DNA Technologies | N/A |
| **rno-miR-874-3p primers: Forward: ATAATATATACTGCCCTGGCCCGAGGGA; Reverse: AGTGCAGGGTCCGAGGTATT** | Integrated DNA Technologies | N/A |
| **rno-miR-1188-5p primers: Forward: TGGTGTGAGGTTGGGCCAG; Reverse: AGTGCAGGGTCCGAGGTATT** | Integrated DNA Technologies | N/A |
| **rno-miR-130b-3p primers: Forward: CGCAGTGCAATGATGAAAGGGCAT; Reverse: AGTGCAGGGTCCGAGGTATT** | Integrated DNA Technologies | N/A |
| **rno-miR-155-5p primers: Forward: CCGCGCGTTAATGCTAATTGTGAT; Reverse: AGTGCAGGGTCCGAGGTATT** | Integrated DNA Technologies | N/A |
| **rno-miR-34b-5p primers: Forward: CGCAGGCAGTGTAATTAGCTGATTGT; Reverse: AGTGCAGGGTCCGAGGTATT** | Integrated DNA Technologies | N/A |
| **rno-miR-34c-5p primers: Forward: CCGCAGGCAGTGTAGTTAGCT; Reverse: AGTGCAGGGTCCGAGGTATT** | Integrated DNA Technologies | N/A |
| **rno-miR-15b-3p primers: Forward: CCGCCGAATCATTATTTGCTGCTCTA; Reverse: AGTGCAGGGTCCGAGGTATT** | Integrated DNA Technologies | N/A |
| **rno-miR-27b-5p primers: Forward: GCCGCTTCACAGTGGCTAAG; Reverse: AGTGCAGGGTCCGAGGTATT** | Integrated DNA Technologies | N/A |
| **hsa-miR-130a-3p primers: Forward: GCCGCGCAGTGCAATGTTAAA; Reverse: AGTGCAGGGTCCGAGGTATT** | Integrated DNA Technologies | N/A |
| **hsa-miR-1908-5p primers: Forward: TAATATATCGGCGGGGACGGCGA; Reverse: AGTGCAGGGTCCGAGGTATT** | Integrated DNA Technologies | N/A |
| **hsa-miR-27-5p primers: Forward: TGCCGTAGGGCTTAGCTGCTTG; Reverse: AGTGCAGGGTCCGAGGTATT** | Integrated DNA Technologies | N/A |
| **hsa-miR-22-3p primers: Forward: TCCGCAAGCTGCCAGTTGAAG; Reverse: AGTGCAGGGTCCGAGGTATT** | Integrated DNA Technologies | N/A |
| **hsa-miR-29c-5p primers: Forward: TCCGCTGACCGATTTCTCCTG; Reverse: AGTGCAGGGTCCGAGGTATT** | Integrated DNA Technologies | N/A |
| **hsa-miR-17-5p primers: Forward: CCGCGCAAAGTGCTTACAGTGC; Reverse: AGTGCAGGGTCCGAGGTATT** | Integrated DNA Technologies | N/A |
| **hsa-miR-20a-5p primers: Forward: GCCGCGCTAAAGTGCTTATAGTGC; Reverse: AGTGCAGGGTCCGAGGTATT** | Integrated DNA Technologies | N/A |
| **hsa-miR-144-3p primers: Forward: GCCGCGCGGTACAGTATAGATGA; Reverse: AGTGCAGGGTCCGAGGTATT** | Integrated DNA Technologies | N/A |

**Table S4. Demographic information of the long-term exercised and sedentary people cohort, used for sequencing.**

|  | **Sedentary (n=25)** | **Exercise (n=25)** | ***P*** |
| --- | --- | --- | --- |
| **Gender^a^ (male/female)** | 25/0 | 25/0 | >0.05 |
| **Age^b^ (years) (mean±SD)** | 37.4±5.07 | 36.72±5.34 | >0.05 |
| **Height^b^ (cm) (mean±SD)** | 171.9±7.42 | 172.92±4.84 | >0.05 |
| **Weight^b^ (kg) (mean±SD)** | 70.7±9.05 | 70.29±7.01 | >0.05 |
| **Body Mass Index^b^ (BMI) (mean±SD)** | 23.9±2.53 | 23.48±1.98 | >0.05 |
| **Systolic BP^b^ (mmHg) (mean±SD)** | 122.44±12.81 | 121.24±14.85 | >0.05 |
| **Diastolic BP^b^ (mmHg) (mean±SD)** | 75.56±10.22 | 74.96±10.08 | >0.05 |
| **Smoking^a^ (yes/no)** | 10/15 | 9/16 | >0.05 |
| **Drinking alcohol^a^ (yes/no)** | 15/10 | 13/12 | >0.05 |
| **Drinking tea^a^ (yes/no)** | 9/16 | 11/14 | >0.05 |
| **Drinking coffee^a^ (yes/no)** | 7/18 | 13/12 | >0.05 |
| **Exercise frequency^b^**  **(times/week) (mean±SD)** | 0±0 | 4.12±0.65 | <0.001 |
| **Exercise duration^b^**  **(months) (mean±SD)** | 0±0 | 83.56±16.78 | <0.001 |

**^a^*p*-values are calculated with a chi-square test.**

**^b^*p*-values are calculated with unpaired two-tailed Student’s *t*-test.**

**Table S5. Demographic information of the long-term exercised and sedentary people cohort, used for validation.**

|  | **Sedentary (n=22)** | **Exercise (n=24)** | ***P*** |
| --- | --- | --- | --- |
| **Gender^a^ (male/female)** | 25/0 | 25/0 | >0.05 |
| **Age^b^ (years) (mean±SD)** | 35.91±6.01 | 35.75±6.42 | >0.05 |
| **Height^b^ (cm) (mean±SD)** | 172.69±5.04 | 172.46±5.33 | >0.05 |
| **Weight^b^ (kg) (mean±SD)** | 73.76±9.33 | 72.99±9.57 | >0.05 |
| **Body Mass Index^b^ (BMI) (mean±SD)** | 24.71±2.75 | 24.48±2.54 | >0.05 |
| **Systolic BP^b^ (mmHg) (mean±SD)** | 123.36±16.99 | 118.83±11.32 | >0.05 |
| **Diastolic BP^b^ (mmHg) (mean±SD)** | 72.85±6.96 | 71.83±8.32 | >0.05 |
| **Smoking^a^ (yes/no)** | 0/25 | 0/25 | >0.05 |
| **Drinking alcohol^a^ (yes/no)** | 0/25 | 0/25 | >0.05 |
| **Drinking tea^a^ (yes/no)** | 0/25 | 0/25 | >0.05 |
| **Drinking coffee^a^ (yes/no)** | 0/25 | 0/25 | >0.05 |
| **Exercise frequency^b^**  **(times/week) (mean±SD)** | 0±0 | 3.79±1.5 | <0.001 |
| **Exercise duration^b^**  **(months) (mean±SD)** | 0±0 | 92.38±24.65 | <0.001 |

**^a^*p*-values are calculated with a chi-square test.**

**^b^*p*-values are calculated with unpaired two-tailed Student’s *t*-test.**

**Table S6. Key reagents, software, and sources used in this study.**

| **REAGENT or RESOURCE** | **SOURCE** | **IDENTIFIER** |
| --- | --- | --- |
| **Chemicals, peptides, and recombinant proteins** | | |
| TRIzol | Thermo Scientific | Cat# 15596-018 |
| DAPI | Thermo Scientific | Cat# D1306 |
| PKH26 Red Fluorescent Cell Linker | Sigma-Aldrich | Cat# MIDI26 |
| DiD fluorescent probe | Beyotime | Cat# C1039 |
| ExoCoupl^TM^ exosomes surface modification bioorthogonal coupling assay kit | Echo Biotech | Cat# EC-01-02 |
| GW4869 | MCE | Cat# HY-19363 |
| Rapamycin | MCE | Cat# HY-10219 |
| Total RNA Extraction Regent | Vazyme | Cat# R401-01-AA |
| RIPA buffer (10x) | Sigma-Aldrich | Cat# R0278 |
| Protease Inhibitor Cocktail | MCE | Cat# HY-K0010 |
| Phosphatase Inhibitor Cocktail I | MCE | Cat# HY-K0021 |
| Phosphatase Inhibitor Cocktail II | MCE | Cat# HY-K0022 |
| PEG300 | MCE | Cat# HY-Y0873 |
| DMSO | Solarbio | Cat# D8371 |
| Tween-80 | Solarbio | Cat# T8360 |
| Lipofectamine 3000 | Thermo Scientific | Cat# L3000-015 |
| **Critical commercial assays** | | |
| Mir-X miRNA First-Strand Synthesis Kit | Takara | Cat# 638315 |
| Mir-X miRNA qRT-PCR TB Green® Kit | Takara | Cat# 638316 |
| TB Green® Premix Ex Taq™ II | Takara | Cat# RR820B |
| miRNeasy Serum/Plasma Kit | Qiagen | Cat# 217184 |
| miRCURY LNA RT Kit | Qiagen | Cat# 339340 |
| miRCURY LNA SYBR Green PCR Kit | Qiagen | Cat# 339345 |
| UniSp6 | Qiagen | Cat# 339306-YP00203954 |
| Super ECL Plus | US Everbright | Cat# S6009M |
| S-100 Protein Detection KIT | MX Biotechnologies | Cat# KIT-9720 |
| DAB Detection Kit | MX Biotechnologies | Cat# DAB-0031 |
| **Software and algorithms** | | |
| ImageJ | NIH | https://www.graphpad.com/ |
| Image Lab (Version 6.1) | Bio-Rad | https://www.bio-rad.com/ |
| Graphpad Prism 8.0 | Graphpad | https://www.graphpad.com/ |
| SuperMaze | Xinruan | https:www.softmaze.com/ |
| Clamfit 11.0.3 | Axon | https://www.MolecularDevices.com/ |
| ChemiDoc MP Imaging System | Bio-Rad | https://www.bio-rad.com/ |
| TopSpin 6.0 | Bruker | https://topspin.en.softonic.com/ |
| ImageScope | Leica | https://www.leica-microsystems.com/ |
| SnapGene | GSL Biotech | https://www.snapgene.cn/ |
